# Cell-Free DNA Sequencing of Intraocular Fluid as Liquid Biopsy in the Diagnosis of Vitreoretinal Lymphoma

**DOI:** 10.3389/fonc.2022.932674

**Published:** 2022-07-19

**Authors:** Junxiang Gu, Tingting Jiang, Shixue Liu, Bo Ping, Ruiwen Li, Wenwen Chen, Ling Wang, Xin Huang, Gezhi Xu, Qing Chang

**Affiliations:** ^1^ Department of Ophthalmology, Eye and ENT Hospital of Fudan University, Fudan University, Shanghai, China; ^2^ Shanghai Key Laboratory of Visual Impairment and Restoration, Eye and ENT Hospital of Fudan University, Fudan University, Shanghai, China; ^3^ National Health Commission (NHC) Key Laboratory of Myopia (Fudan University), Key Laboratory of Myopia, Chinese Academy of Medical Science, Shanghai, China; ^4^ Department of Pathology, Fudan University Shanghai Cancer Center, Fudan University, Shanghai, China; ^5^ Department of Nursing, Eye and ENT Hospital of Fudan University, Fudan University, Shanghai, China

**Keywords:** vitreoretinal lymphoma, cell-free DNA sequencing, diagnostic vitrectomy, liquid biopsy, diagnosis

## Abstract

**Purpose:**

To seek novel diagnostic approaches, we improved the workflow of cell-free DNA (cfDNA) sequencing and evaluated its feasibility in vitreoretinal lymphoma (VRL) specimens; the profile of mutations was preliminarily analyzed for potential diagnostic value.

**Methods:**

The study was a diagnostic trial. 23 eyes of 23 patients with VRL and 25 eyes of 25 patients with inflammatory eye diseases were enrolled. Approximate 500μl undiluted vitreous humor and 10ml diluted vitreous fluid was obtained through diagnostic vitrectomy and sent for cytopathological examinations. 500μl of the diluted vitreous fluid was spared for cfDNA sequencing. For cfDNA sequencing, DNA fragmentation procedure was added to the workflow to improve the extraction efficiency; mutations detected were analyzed for potential diagnostic model. The sensitivity and specificity of the cytopathology and cfDNA sequencing were compared. The clinical manifestations were preliminarily analyzed for potential correlations with the genotypes.

**Results:**

CfDNA sequencing was accomplished in 23 eyes with VRL and 20 eyes with inflammatory eye diseases. VRL-related mutated genes included *MYD88* (18 eyes, 78%), *ETV6* (11 eyes, 48%), *PIM1* (11 eyes,48%), *BTG2* (7 eyes, 30%), *IRF4* (7 eyes, 30%), *CD79B* (6 eyes, 26%), *LRP1B* (6 eyes, 26%), etc. Logistic regression based on the mutations of *MYD88* and *ETV6* was of the potential for the diagnosis of VRL (P<0.001, adjusted R2 = 0.789, sensitivity 0.913, specificity 0.950); by comparison, the sensitivity and specificity of the vitreous cytopathology were 0.826 and 1.000, respectively. Further analysis of the mutation profile showed that patients carrying *CD79B* mutation tended to have higher intraocular interleukin-10 level (P=0.030), that CARD11 mutation was correlated with younger age at ocular onset (P=0.039), and that patients with intracranial involvement carried more multiple-site mutations in the *BTG2* gene (P=0.013).

**Conclusions:**

The improved workflow of CfDNA sequencing is of sound feasibility in a limited amount of vitreous humor. The logistic model based on the mutations could help to provide reliable clues for the diagnosis of VRL.

## Introduction

Primary vitreoretinal lymphoma (primary VRL, PVRL) is a rare type of ocular malignancies, the majority of which is aggressive B-cell lymphoma, usually diffuse large B-cell lymphoma (DLBCL) (1, 2). The diagnosis of VRL has long been challenging because the clinical manifestations of VRL extensively mimic uveitis, which may include vitreous cell, retinal infiltration and sub-retinal pigment epithelium (RPE) lesion (3). Efforts have been made to differentiate VRL from uveitis. Interleukin detection of intraocular fluid combined with Interleukin Score for Intraocular Lymphoma Diagnosis (ISOLD) scores greatly facilitates the differential diagnosis of VRL. Despite such efforts, for malignancies, the gold standard of the confirmed diagnosis remains cytopathologic analysis (4, 5). However, the detection rate of cytopathology in VRL is still unsatisfactory. Firstly, for the maintenance of safe intraocular pressure during surgery, intraocular fluid specimens are limited in volume and sometimes diluted. Besides, corticosteroids administration, cell damage from the vitreous cutter and rapid degeneration of lymphoma cells also make it hard to obtain qualified cellular specimens for cytopathologic analysis (6).

High-throughput gene detection provides new solutions for the diagnosis of VRL. Detection of *MYD88* ^L265P^ mutation through polymerase chain reaction (PCR) based techniques is of good feasibility (7–9) but might be invalid in the cases without *MYD88* mutation. Whole exon sequencing (WES) and single-cell sequencing can provide detailed information about the mutation and expression of the genes (10, 11), but these techniques require well-treated samples and might lead to additional consumption of the limited cellular specimen predestined for cytopathologic examinations.

Circulating tumor DNA (ctDNA), or cell-free DNA (cfDNA) sequencing, is another sequencing technique detecting free DNA fragments released in body fluid (12), which has already been applied to the early diagnosis of different kinds of solid tumors (13–15). By contrast with other high-throughput sequencing techniques, the sample for cfDNA sequencing is easier to collect and bears dilution, which might be a feasible solution for the diagnosis of VRL.

In this study, we evaluated the feasibility and reliability of cfDNA sequencing in the diagnosis of VRL, and preliminarily analyzed the profile of gene mutations and its relation with clinical characteristics.

## Materials and Methods

This was a single-center diagnostic trial. The study was approved by the Institutional Review Board (IRB) and the ethics committee of the Eye and ENT Hospital of Fudan University. Informed consent was obtained from the participants. All the examinations were performed according to the Declaration of Helsinki; the samples and genetic information were managed in accordance with the regulations of the Human Genetic Resource Administration of China (HGRAC).

Consecutive patients who were clinically suspected of VRL and underwent diagnostic vitrectomy at Eye and ENT Hospital of Fudan University between January 2015 to September 2020 were reviewed. The patients were included in case of (1) positive vitreous biopsy outcomes, (2) typical ocular manifestations with positive cerebral biopsy outcomes and (3) typical ocular manifestations which were glucocorticoid-resistant but methotrexate-sensitive in combination with elevated interleukin-10 (IL-10) level (>50 pg/ml) and interleukin-10/interleukin-6 (IL-10/IL-6) ratio (>1) in the intraocular fluids. The exclusion criteria included (1) insufficient vitreous sample for cytopathologic examinations, and (2) previous ocular treatment (e.g. vitrectomy, intravitreal or systemic chemotherapy) which might affect the initial ocular manifestations. In order to evaluate the diagnostic and differential value of cfDNA sequencing, patients with infectious or non-infectious uveitis were enrolled as the control group if (1) the vitrectomy and vitreous biopsy were accomplished, and (2) the etiological diagnoses for the intraocular inflammation were confirmed.

Glucocorticoid, if administrated systemically, was withdrawn at least two weeks before the diagnostic vitrectomy. Diagnostic vitrectomy was performed through the standard 25 G 3-port pars plana vitrectomy system. Approximate 500μl of undiluted vitreous sample was aspirated using the vitrectomy cutter at 600 cuts per minute before the intraocular infusion was turned on; then 10ml of diluted vitreous sample was obtained at the same cutting rate. After sampling, 500μl of the diluted vitreous was spared for cfDNA sequencing. Then, 300μl of the undiluted sample and all the remaining diluted sample were sent as soon as possible to the department of pathology of Fudan University Shanghai Cancer Center for the routine cytopathologic workup which included (1) microscopy of the smears and cell pellet sections, (2) immunohistochemistry, (3) gene rearrangement test, and (4) Sanger sequencing of *MYD88* gene. The remaining undiluted vitreous was stored at -80°C for subsequent potential assays.

CfDNA sequencing was performed to the spared diluted vitreous samples. Cells and debris were removed by the centrifugation at 16,000g for 10 min at 4°C. CfDNA was extracted by the QIAamp Circulating Nucleic Acid Kit (Qiagen, Venlo, The Netherlands). The quality control for the amount of cfDNA was then performed by the Agilent 4150 or 4200 TapeStation system (Agilent Technologies, Santa Clara, CA, USA). The sample was considered qualified if the cfDNA ranging from 100bp to 200bp reached 10ng. Qualified samples were then processed with QIAseq Ultralow input Library Kit (Qiagen, Venlo, The Netherlands) for library preparation. For the samples with the total DNA exceeding 20ng but insufficient cfDNA within the optimal range, DNA fragmentation was performed by KAPA HyperPlus Kit (Roche Sequencing, Pleasanton, CA, USA) before library preparation. Samples containing total DNA less than 20ng were considered not qualified for sequencing. The targeted regions of the selected 646 genes (detailed in **Supplementary Table 1**) were then captured using the customized xGen Lockdown Probe Pools (Integrated DNA Technologies, Coralville, IA, USA) and the sequencing was performed using the NovaSeq 6000 system (Illumina, San Diego, CA, USA). The data were considered qualified when more than 90% of the targeted regions reached at least 20% of the average sequencing depth. Somatic mutations and indels were analyzed using VarScan (version 2.3.9). Single nucleotide variants (SNVs) were evaluated according to the databases including ClinVar and InterVar; the SNVs annotated as benign were eliminated. Additional cfDNA sequencing was applied to a paired aqueous humor sample of one subject in order to test the consistency between the aqueous humor and vitreous humor.

Baseline clinical data including age at onset, gender and history of ocular or systemic diseases were reviewed. The fundus was evaluated using slit-lamp biomicroscopy, optical coherence tomography (OCT), widefield swept source OCT, ultra-widefield scanning laser ophthalmoscopy and widefield autofluorescence. For VRL patients, the preceding examinations were performed at baseline and then monthly; brain magnetic resonance imaging (MRI) with contrast-enhancement, or positron emission tomography/computed tomography (PET/CT) if necessary, was performed at baseline and every 6 months to detect central nervous system (CNS) progression.

Treatment was given in accordance with the recommendation of the International Primary Central Nervous System Lymphoma Collaborative Group (2). For VRL patients with bilateral involvement or CNS involvement, systemic high-dose MTX based chemotherapy followed by intravitreal MTX chemotherapy was suggested; while for unilaterally involved patients without CNS conditions, initial intravitreal MTX chemotherapy was recommended. Intravitreal injections were performed twice weekly for 2 weeks, then once weekly for 4 weeks, and afterwards once monthly for 6 months. Progression-free survival (PFS) was defined as the interval from the diagnosis of VRL to the first recurrence of the disease or the date of the last follow-up in patients without relapse.

The statistics were conducted using SPSS version 22.0 (SPSS Inc., Chicago, IL, USA) and SAS version 9.4 (SAS Institute Inc., Cary, NC, USA). The normality was tested using Kolmogorov-Smirnov test. Normally-distributed continuous variables were represented in the form of mean ± standard deviation, while other variables were represented as median [interquartile range]. The 95% confidential intervals (95%CI) of the statistical estimates were calculated. T-test was used to compare the age between the VRL group and the control group. The number of mutations was compared between the two groups through Wilcoxon rank-sum test. In the analysis of the diagnostic trial, a) Fisher’s exact test was used to evaluate the difference in the incidence of each mutation and the false discovery rate (FDR) was controlled using the Benjamini-Hochberg method; b) logistic regression model was preformed using Firth’s penalized maximum likelihood estimation (PMLE) to evaluate the diagnostic value of the mutations detected; c) the sensitivity, specificity, Youden index and the area under the receiver operating characteristic curve (AUC of ROC curve) of different diagnostic approaches were then calculated. Kappa test was used to evaluate the consistency of Sanger sequencing and cfDNA sequencing. In the phenotype analysis, the clinical characteristics were compared between the mutant type (MT) and wild type (WT) of different genes using Fisher’s exact test and Wilcoxon rank-sum test. To further investigate the potential impact of the genes with multiple-site mutations, the correlation between the number of mutated sites and clinical manifestations was analyzed by Wilcoxon rank-sum test and Spearman’s correlation. In the prognosis analysis, the PFS of major mutant types was described using Kaplan-Meier method. The *P* values < 0.05 were considered statistically significant. The *post-hoc* power (1-β) was calculated for reference in case of *P* < 0.05 using the POWER procedure of SAS version 9.4 (SAS Institute Inc., Cary, NC, USA) due to the limited sample size.

## Results

A total of 23 eyes of 23 VRL patients were included as the VRL group and 25 eyes of 25 patients with infectious or non-infectious uveitis were included as the control group, of which the mean age was 56 ± 12 years and 49 ± 14 years respectively. The difference in age between the two groups was of no statistical significance (T-test, *P*=0.066).

In the VRL group, six patients (26%) were diagnosed with PCNSL before the ocular involvement. Seventeen patients were diagnosed with PVRL, of whom 5 patients (22%) developed CNS involvement in 11 ± 7 months (range 3-21 months) while the rest (12 patients, 52%) did not. The median length of the follow-up periods was 20 ± 14 months (range: 6-53 months). At baseline, vitreous cell, retinal infiltration and sub-RPE lesion were observed in 23 eyes (100%), 5 eyes (22%) and 10 eyes (43%) respectively (**Table 1**).

**Table 1:** Demographic features, cytopathologic outcomes and fundus findings of the included patients.

Patient	Gender	Age(y)	CNS involvement*	Follow-up periods	Time to CNS involvement	IG H clonality	IG K clonality	Cytology	Sanger-seq for *MYD88*	Fundus findings		
										Vitreous cell	Retinal infiltration	Sub-RPE lesion
VRL1	Female	59	PCNSL+VRL	21 m	/	+	+	+	+	+	–	–
VRL2	Female	59	PVRL+CNSL	53 m	+11 m	–	–	+	–	+	–	–
VRL3	Male	59	PCNSL+VRL	11 m	/	+	+	+	–	+	–	–
VRL4	Female	78	PVRL	6 m	/	+	+	+	+	+	+	+
VRL5	Female	48	PCNSL+VRL	30 m	/	+	+	+	+	+	+	–
VRL6	Female	55	PVRL+CNSL	12 m	+15 m	+	+	+	+	+	–	–
VRL7	Male	60	PVRL+CNSL	33 m	+21 m	+	–	+	+	+	–	+
VRL8	Female	35	PVRL+CNSL	6 m	+5 m	+	+	–	–	+	–	+
VRL9	Female	32	PCNSL+VRL	47 m	/	+	+	+	+	+	–	–
VRL10	Female	55	PVRL	40 m	/	–	–	+	+	+	–	–
VRL11	Female	65	PCNSL+VRL	20 m	/	+	+	+	+	+	–	–
VRL12	Female	52	PVRL	41 m	/	+	+	+	+	+	–	–
VRL13	Male	73	PVRL	13 m	/	–	–	+	–	+	–	–
VRL14	Female	57	PVRL	9 m	/	+	+	+	+	+	–	–
VRL15	Female	67	PVRL	24 m	/	+	+	+	+	+	–	+
VRL16	Female	48	PVRL+CNSL	13 m	+3 m	–	+	–	+	+	+	+
VRL17	Female	32	PVRL	24 m	/	+	+	–	+	+	+	–
VRL18	Female	50	PCNSL+VRL	16 m	/	–	+	–	–	+	+	–
VRL19	Female	60	PVRL	17 m	/	–	+	–	–	+	–	+
VRL20	Male	57	PVRL	9 m	/	–	–	+	+	+	–	+
VRL21	Female	50	PVRL	7 m	/	–	–	–	–	+	–	+
VRL22	Female	58	PVRL	9 m	/	–	–	–	–	+	–	+
VRL23	Male	73	PVRL	7 m	/	–	–	–	–	+	–	+

*PVRL referred to patients with primary VRL without CNS involvement till the latest visit; PVRL+CNSL referred to patients with primary VRL and subsequent CNS progression; PCNSL+VRL referred to patients with primary CNSL and subsequent ocular progression.

CNS, central nervous system; CNSL, central nervous system lymphoma; VRL, vitreoretinal lymphoma; Sanger-seq, Sanger sequencing of cell pellet; cfDNA-seq, cell-free DNA sequencing; RPE, retinal pigment epithelium.

In the control group, the etiology included endophthalmitis (8 eyes, 32%), acute retinal necrosis (ARN, 8 eyes, 32%), ocular tuberculosis (5 eyes, 20%), toxoplasmosis (1 eye, 4%), syphilis (1 eye, 4%), intermediate uveitis (1 eye, 4%) and toxocariasis (1 eye, 4%).

### Cytopathologic Outcomes

In the VRL group, malignant lymphocytes were directly observed in the cytopathologic examination of 15 eyes (65%); another 4 eyes (17%) were confirmed by adequate atypical lymphocytes in combination with immunohistochemistry, gene rearrangement and Sanger sequencing of *MYD88*; the remaining 4 eyes (17%) were negative for the whole workup of cytopathology. Specifically, gene rearrangements of immunoglobulin heavy chain (IGH) and kappa light chain (IGK) were detected in 13 (57%) eyes and 15 (68%) eyes respectively; *MYD88* ^L265P^ mutation was detected in 14 eyes by Sanger sequencing (**Table 1**). The diagnosis of the 4 eyes negative for cytopathologic workup were retrospectively confirmed by the glucocorticoid-resistant but methotrexate-sensitive ocular manifestations in combination with elevated IL-10 level or IL-10/IL-6 ratio in the intraocular fluids. In the control group, no vitreous sample was positive for malignant or atypical lymphocytes.

### Comparison of cfDNA Sequencing in Paired Aqueous Humor and Vitreous Samples

The paired aqueous humor and vitreous samples of one patient (VRL1) were sent for cfDNA sequencing, which showed exactly the same mutated genes and corresponding sites of the mutations (**Table 2**).

**Table 2:** Comparison of the sequencing outcomes between the paired aqueous humor and vitreous humor.

Aqueous humor				Vitreous humor			
Gene	Exon	HGVSc	VAF	Gene	Exon	Mutation	VAF
***AMER1***	exon2	c.1699C>A	44.97%	***AMER1***	exon2	c.1699C>A	46.51%
***CREBBP***	exon26	c.4337G>A	55.60%	***CREBBP***	exon26	c.4337G>A	50.55%
***ETV6***	exon6	c.1060T>C	35.79%	***ETV6***	exon6	c.1060T>C	49.46%
***INHA***	exon2	c.658C>T	45.21%	***INHA***	exon2	c.658C>T	51.58%
***IRF4***	exon2	c.208C>G	58.20%	***IRF4***	exon2	c.208C>G	40.52%
***MYD88***	exon5	c.794T>C	32.64%	***MYD88***	exon5	c.794T>C	31.11%
***PIM1***	exon4	c.367C>T	41.29%	***PIM1***	exon4	c.367C>T	43.14%
	exon4	c.385C>G	55.89%		exon4	c.385C>G	50.98%
	exon4	c.496C>T	35.36%		exon4	c.496C>T	46.61%
	exon4	c.550C>T	44.80%		exon4	c.550C>T	47.35%

HGVS, Human Genome Variation Society; VAF, variant allele frequency.

### Overview of cfDNA Sequencing

CfDNA sequencing was accomplished in 43 eyes (VRL: 23, control: 20), among which 11 samples (VRL: 1, control: 10) underwent enzymatic DNA fragmentation before sequencing; while the remaining 5 eyes from the control group didn’t contain sufficient amount of nucleic acid required for the preparation of libraries. After the elimination of documented benign mutations, 403 mutations of 186 genes and 166 mutations of 125 genes were discovered respectively in the VRL group and the control group; the mean frequency of mutation was 18 ± 8 and 12 ± 7 mutations per eye respectively between the two group, the difference in which was of no statistical significance (T-test, *P*=0.058). The differentially distributed mutations were further aligned with GeneCards database and published literatures (see **Supplementary Figure 1**).

For the VRL group, frequently (n≥3) observed genes with mutations included *MYD88* (18 eyes), *ETV6* (11 eyes), *PIM1* (11 eyes), *BTG2* (7 eyes), *IRF4* (7 eyes), *CD79B* (6 eyes), *LRP1B* (6 eyes), *ANKRD11* (5 eyes), *BTG1* (5 eyes), *CARD11* (5 eyes), *MYC* (5 eyes), *KMT2D* (4 eyes), *AXL* (3 eyes), *BCOR* (3 eyes), *CREBBP* (3 eyes), *KMT2A* (3 eyes), *MCL1* (3 eyes), *PRDM1* (3 eyes) and *STAT3* (3 eyes), (**Table 3**) among which all except *ETV6*, *AXL* and *CREBBP* were specific to the VRL group. *MYD88* ^L265P^ was the only mutated site of *MYD88* gene. For the control group, the genes with mutations detected more than twice included *EP300* (3 eyes), *INPP4A* (3 eyes), *IRS1* (3 eyes), *PTCH1* (3 eyes) and *SLIT2* (3 eyes). Overlapping mutated genes with the VRL group included *ETV6* (1 eye), *AXL* (1 eye) and *CREBBP* (1 eye). All the mutations were detailed in **Supplementary Table 2**.

**Table 3:** Mutations of the hematological malignancy-related genes detected in the VRL group.

Gene	Eye (n = 23)	Percentage	Frequency	Gene	Eye(n = 23)	Percentage	Frequency
***MYD88***	18	78%	18	***BCOR***	3	13%	3
***ETV6***	11	48%	12	***KMT2A***	3	13%	3
***PIM1***	11	48%	59	***PRDM1***	3	13%	3
***BTG2***	7	30%	15	***STAT3***	3	13%	3
***IRF4***	7	30%	11	***SOCS1***	2	9%	3
***CD79B***	6	26%	6	***CCND3***	2	9%	2
***LRP1B***	6	26%	6	***FOXO1***	2	9%	2
***BTG1***	5	22%	7	***GNA13***	2	9%	2
***MYC***	5	22%	7	***NFKBIA***	2	9%	2
***ANKRD11***	5	22%	5	***TP53***	2	9%	2
***CARD11***	5	22%	5	***CD79A***	1	4%	1
***KMT2D***	4	17%	5	***CXCR4***	1	4%	1
***CREBBP***	3	13%	4	***DMD***	1	4%	1
***MCL1***	3	13%	4	***MEF2B***	1	4%	1
***AXL***	3	13%	3	***NOTCH1***	1	4%	1

### Diagnostic Trial

Fisher’s exact test was performed to compare the incidences of different mutations between the two groups. Genes with higher incidences of mutation in VRL patients included *MYD88* (P<0.001; FDR-adjusted P<0.001) and *PIM1* (P<0.001; FDR-adjusted P<0.001). The AUC of ROC curves for *MYD88* and *PIM1* were 0.891 (95%CI: 0.786 to 0.997) and 0.739 (95%CI: 0.589 to 0.889), respectively. Other mutations possibly related to VRL included *ETV6*, *BTG2*, *IRF4*, *CD79B* and *LRP1B*, of which the P values were respectively 0.002, 0.010, 0.010, 0.023 and 0.023 but turned insignificant after FDR control (FDR-adjusted P=0.111, 0.332, 0.332, 0.545 and 0.545, respectively).

**Figure 1:**
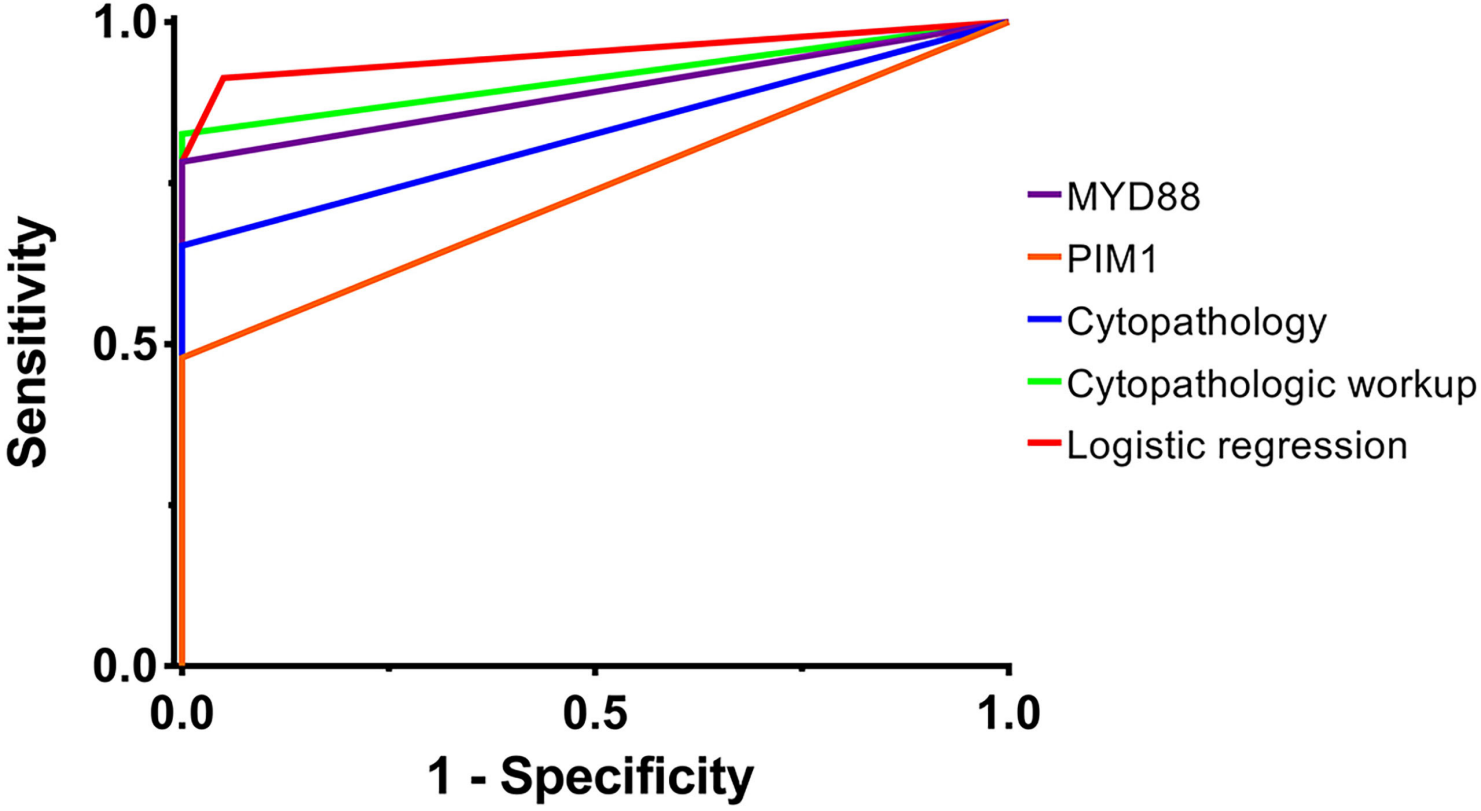
The ROC curves of different diagnostic methods. The ROC curves of different diagnostic methods are plotted, including *MYD88* mutation, *PIM1* mutation, cytopathology, cytopathologic workup and logistic regression. The AUCs of the ROC curves were 0.891 (95%CI: 0.786 to 0.997), 0.739 (95%CI: 0.589 to 0.889), 0.826 (95%CI: 0.697 to 0.955), 0.913 (95%CI: 0.818 to 1.000) and 0.951 (95%CI: 0.880 to 1.000), respectively. AUC, area under the curve; ROC curve, receiver operator characteristic curve. purple, *MYD88* mutation; orange, *PIM1* mutation; blue, cytopathology; green, cytopathologic workup; red, logistic regression.

Logistic regression was performed through Firth’s PMLE due to the quasi-complete separation of several mutations. *MYD88*, *PIM1*, *ETV6*, *BTG2*, *IRF4*, *CD79B* and *LRP1B* (mutations with Fisher’s exact test P<0.05) were introduced to the regression model in a forward procedure. Mutations correlated with the diagnosis of VRL included *MYD88* and *ETV6* (P<0.001, adjusted R^2 =^ 0.789); the odds ratio estimates were 163.880 (95%CI: 14.577 to >999.999) and 17.870 (95%CI: 2.002 to 260.751), respectively. The regression coefficients of *MYD88*, *ETV6* and the intercept were 5.099 (95%CI: 2.680 to 10.071), 2.883 (95%CI: 0.694 to 5.564) and -2.048 (95%CI: -3.654 to -0.906), respectively. The *post-hoc* power (1-β) was >0.999. The sensitivity and specificity were respectively 0.913 and 0.950 when the Youden index reached 0.863. The AUC of ROC curve was 0.951 (95%CI: 0.880 to 1.000). (see **Figure 1**)

To verify the reliability of cfDNA sequencing, we further compared the results of *MYD88* ^L265P^ mutation which were respectively detected by cfDNA sequencing from the vitreous and Sanger sequencing from the cell pellets by means of Kappa test (**Table 4**). The *P* value was 0.002 and the Kappa coefficient was 0.603, which indicated good consistency between the two methods applied respectively in different types of specimens. Moreover, all the cases positive for *MYD88* ^L265P^ mutation in Sanger sequencing were also positive in cfDNA sequencing without any false positive mutation detected at other sites of *MYD88*. Besides, cfDNA sequencing detected another 4 cases with *MYD88* ^L265P^ mutation of which the Sanger sequencing outcomes were negative.

**Table 4:** Comparison of *MYD88* ^L265P^ detection between cfDNA sequencing and Sanger sequencing.

		Cell block Sanger sequencing		Total
		*MYD88* ^L265P^ Positive	*MYD88* ^L265P^ Negative	
**cfDNA sequencing**	*MYD88* ^L265P^ Positive	14	4	18
	*MYD88* ^L265P^ Negative	0	5	5
Total		14	9	23

### Phenotype Analysis

The correlations between the mutated genes and clinical manifestations were analyzed using the Fisher’s exact test (binary variables) and Wilcoxon rank-sum test (continuous variables). The *P* values were listed in **Table 5**. *BTG2* mutation was correlated with CNS involvement (*P*=0.027, 1-β=0.533); *CD79B* mutation was correlated with higher initial intraocular IL-10 level (MT vs. WT, 4520.55[2804.04-7616.72] pg/ml vs. 856.60[15.70-1500.00] pg/ml, *P*=0.030, 1-β=0.740); *CARD11* mutation was correlated with earlier ocular onset (MT vs. WT, 65.6 ± 7.3y vs. 53.0 ± 11.6y, *P*=0.039, 1-β=0.714), and so was *AXL* mutation (MT vs. WT, 50.0[41.0-50.0]y vs. 57.5 ± 11.3y, *P*=0.046, 1-β=0.524). In consideration of the sample size, the *post-hoc* statistical power (1-β) of *CD79B* and *CARD11* was above 0.7, which indicated that their correlations with corresponding phenotypes were noteworthy.

**Table 5:** The correlation of the mutations with CNS involvement, age at onset, clinical findings and interleukin levels.

Gene	*MYD88* ^L265P^ mutation*	CNS involvement*	Age^#^	Keratic precipitates*	Fundus findings		Baseline IL-10^#^	Baseline IL-10/6 ratio^#^
					Retinal infiltration*	Sub-RPE lesion*		
***MYD88***	N/A	0.640	0.971	1.000	1.000	1.000	0.325	0.325
***PIM1***	0.317	0.684	0.833	0.680	1.000	0.680	0.880	0.976
***ETV6***	0.640	1.000	0.928	0.680	0.317	1.000	0.235	0.880
***BTG2***	0.621	**0.027 (0.533)**	0.720	1.000	1.000	0.405	0.922	0.820
***IRF4***	1.000	0.193	0.413	0.089	0.142	0.089	0.760	0.720
***ANKRD11***	0.291	0.640	0.491	1.000	1.000	1.000	0.067	0.491
***CD79B***	0.272	1.000	0.973	0.179	0.576	1.000	**0.030 (0.740)**	0.319
***MYC***	0.291	0.155	0.745	1.000	0.291	1.000	0.801	0.745
***BTG1***	0.545	0.640	0.080	0.339	0.291	1.000	0.363	0.491
***CARD11***	1.000	0.317	**0.019(0.732)**	1.000	0.545	0.618	0.638	0.325
***KMT2D***	0.539	0.317	0.907	0.604	0.194	1.000	0.969	0.667
***LRP1B***	0.272	1.000	0.708	0.341	0.576	1.000	0.973	0.101
***CREBBP***	1.000	0.590	0.698	0.229	0.539	0.229	0.404	0.930
***PRDM1***	1.000	0.217	1.000	0.560	0.107	0.560	0.230	0.830
***AXL***	0.539	1.000	**0.046(0.524)**	0.560	0.107	1.000	0.830	0.404
***BCOR***	0.107	0.930	0.309	1.000	0.539	1.000	0.966	0.514
***KMT2A***	0.539	0.590	0.355	1.000	0.539	0.068	0.404	0.698
***MCL1***	0.107	0.590	0.309	1.000	0.107	1.000	0.898	0.139
***STAT3***	0.539	0.093	0.404	0.229	0.107	0.229	0.514	0.698

*P-value of Fisher’s exact test.

^#^P-value of Wilcoxon rank-sum test.

The statistical power (1-β) was calculated and listed in the brackets in case of P<0.05.

The bold values represent the P values which are less than 0.05 (a).

**Figure 2:**
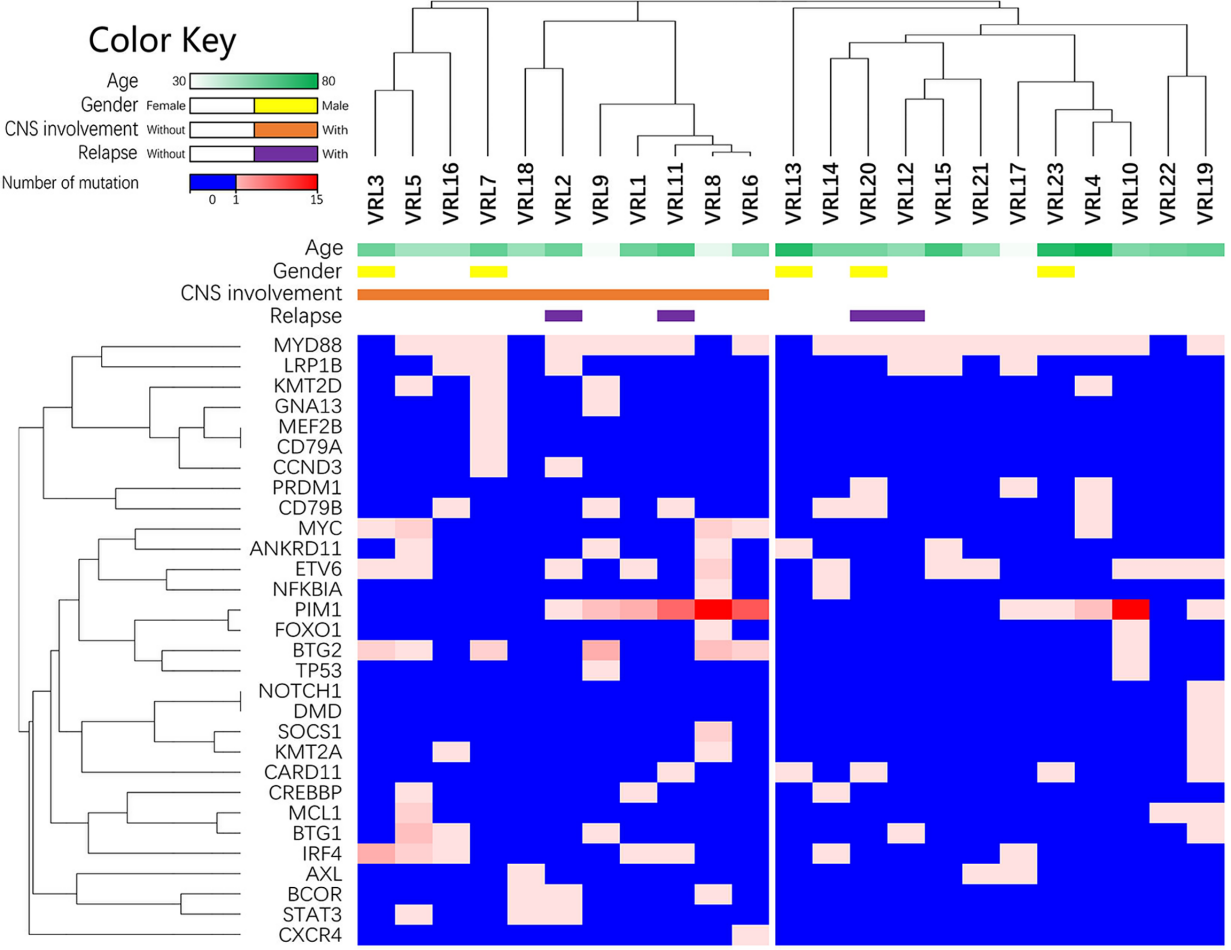
The list of lymphoma-related mutations and the numbers of mutated sites within each gene. This heatmap is the list of lymphoma-related mutations and the numbers of mutated sites in each eye. The blue cells in the heatmap refer to no detected mutation; the spectrum of light red to dark red corresponds to the numbers of mutated sites within each gene.

Since the multiple-site mutation of one single gene was more likely to cause the loss-of-function of its encoding protein, the number of the mutation sites of each single gene was plotted (**Figure 2**) and analyzed for its correlation with the clinical manifestations through the Wilcoxon rank sum test (binary variables) and Spearman’s correlation (continuous variables) (**Table 6**). More mutated sites of *BTG2* gene were detected in patients with CNS involvement than in those without (1 [IQR: 0-2, range: 0-4] vs. 0 [IQR: 0-0, range: 0-1] mutations per eye, *P*=0.013, 1-β=0.772).

**Table 6:** The correlation of genes undergoing multiple-site mutations with CNS involvement, age of onset and clinical findings.

Gene	*MYD88* ^L265P^ mutation*	CNS involvement*	Age^#^	Keratic precipitates*	Fundus findings		Baseline IL-10^#^	Baseline IL-10/6 ratio^#^
					Retinal infiltration*	Sub-RPE lesion*		
***PIM1***	0.353	0.317	0.912	0.568	0.493	0.420	0.930	0.814
***BTG2***	0.492	**0.013 (0.772)**	0.092	0.909	0.436	0.446	0.366	0.516
***IRF4***	0.782	0.110	0.411	0.060	0.117	0.060	0.103	0.649
***BTG1***	0.195	0.493	0.066	0.227	0.213	0.796	0.378	0.563
***MCL1***	0.656	0.674	0.864	0.672	0.524	0.458	0.942	0.143
***MYC***	0.255	0.096	0.558	0.897	0.255	0.897	0.867	0.877
***CREBBP***	0.339	0.563	0.668	0.112	0.656	0.112	0.376	0.836
***KMT2D***	0.258	0.225	0.798	0.397	0.175	0.851	0.876	0.703
***ETV6***	0.352	1.000	0.737	0.699	0.163	0.699	0.179	0.780

*P-value of Wilcoxon rank-sum test.

^#^P-value of Spearman’s correlation.

The statistical power (1-β) was calculated and listed in the brackets in case of P<0.05.

The bold values represent the P values which are less than 0.05 (a).

### Prognosis Analysis

For the VRL group, the mean length of the follow-up was 20 ± 14 months, and the mean PFS was 18 ± 12 months. Three patients had CNS relapse, 2 patients had ocular relapse and 1 patient had both; the intervals from the beginning of follow-up to the progression were 11 ± 7 months for CNS and 27 ± 23 months for the eye.

**Figure 3:**
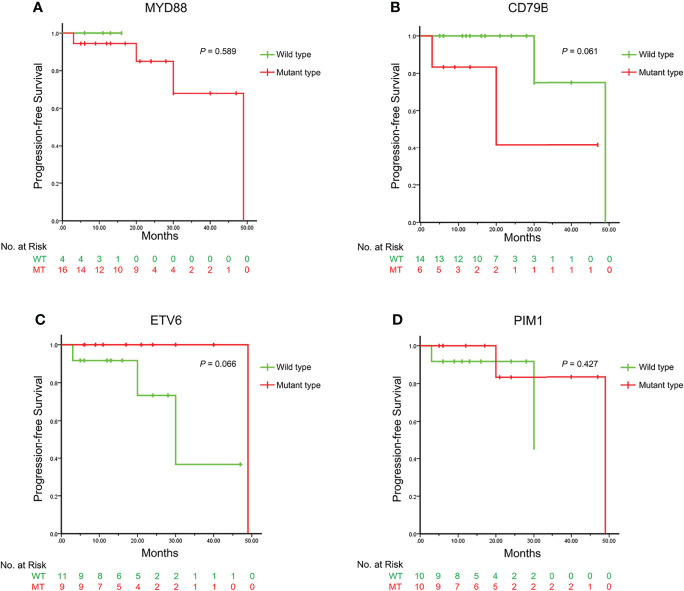
The Kaplan-Meier survival curves illustrating the progression-free survival between different genotypes of the VRL-related genes. The Kaplan-Meier survival curves are plotted according to the genotypes of *MYD88*, *CD79B*, *ETV6* and *PIM1* as **(A–D)** respectively. The curves in green represent the wild types and those in red represent the mutant types. The P values of the Log-rank test are displayed in the upper right, indicating that no single mutant type is directly correlated with the progression-free survival. VRL, vitreoretinal lymphoma.

Kaplan-Meier survival curves were plotted according to different genotypes which were mainly divided by *MYD88*, *CD79B*, *ETV6* and *PIM1* (**Figure 3**). Log rank tests indicated that there was no single mutant type which was definitely correlated with PFS in this cohort (*P*=0.589, *P*=0.061, *P*=0.066 and *P*=0.427, respectively).

## Discussion

In our study, we preliminarily evaluated the feasibility of cfDNA sequencing as liquid biopsy in the diagnosis of VRL. CfDNA sequencing could be performed to a small amount of intraocular fluid and yield reliable and informative profile of the mutations, thus facilitating the diagnosis of VRL.

CfDNA sequencing is a technique detecting the free DNA fragments released in body fluids by tumor cells (12). Also, previous studies showed the feasibility of high-throughput sequencing techniques in vitreous samples (10, 16, 17). In our study, we improved the workflow of cfDNA sequencing for better utilization of DNA fragments in limited amount of vitreous humor. Then we applied the procedure to a clinical cohort of VRL patients and obtained as reliable outcomes as the Sanger sequencing which was considered as the gold standard for sequencing; cfDNA sequencing reported no false positive mutation and further detected 4 more cases with *MYD88* ^L265P^ mutation than Sanger sequencing. Moreover, the consistent sequencing outcomes of the paired aqueous humor and vitreous humor also to some extent verified the repeatability of cfDNA sequencing. Though not as informative as WES or single-cell sequencing, targeted cfDNA sequencing does not require intact cell components and could spare more cellular specimen for routine cytologic examinations. Therefore, cfDNA sequencing is a promising technique which can provide additional sources of genetic information to assist the diagnosis of VRL.

The difficulty in the diagnosis of VRL lies in its masquerading as uveitis (18). In our study, 25 cases with infectious or non-infectious uveitis were matched as the control group. As a result, cfDNA was accomplished in the vitreous humor of 20 eyes, while the remaining 5 samples didn’t contain sufficient amount of nucleic acid required for the sequencing libraries. The cases failing in cfDNA sequencing included ocular tuberculosis (2 eyes), endophthalmitis (1 eyes), syphilis (1 eye), and toxocariasis (1 eye). The possible reason for the failure in sequencing was that patients with uveitis underwent vitrectomy mainly for the complications (e.g. epiretinal membrane) rather than uveitis itself. The inflammation tended to be mild and the level of the free DNA fragments released in the vitreous is lower. In the 20 eyes undergoing cfDNA sequencing, the distribution profile was different from that of the VRL cases according to the clustering (**Supplementary Figure 1**), which made possible subsequent analysis for the diagnostic role of cfDNA sequencing.

Despite the numerous studies on the application of high-throughput sequencing in VRL, there is still a lack of the diagnostic trial data in real-world cohorts. Previous literatures reported several specific mutants in non-Hodgkin lymphoma patients (10, 17, 19, 20). In our study, mutations of *MYD88* (78%), *ETV6* (48%), *PIM1* (48%), *BTG2* (30%), *IRF4* (30%), *CD79B* (26%), *LRP1B* (26%), *ANKRD11* (22%), *BTG1* (22%), *CARD11* (22%), *MYC* (22%) and *KMT2D* (17%) were observed, which was consistent with the published studies. We further enrolled logistic regression to attempt a diagnostic model based on the mutation analysis of multiple genes; mutations of *MYD88* and *ETV6* were included in the logistic model of which the sensitivity, specificity and AUC of ROC curve were 0.913, 0.950 and 0.951, respectively; by comparison, those of routine cytopathology were 0.826, 1.000 and 0.913 respectively. Possible advantage of cfDNA sequencing in detection rate lay in that the intraocular fluid for cfDNA sequencing contained all the free DNA fragments released by all the cells alive or lytic, while cytopathology required intact malignant cells which might be already lytic before fixation or even not contained in the specimens obtained. However, overlapping mutation of ETV6 was also observed in the control group, for which the possible explanation might lie in that the changes in amino acid sequence might not necessarily lead to the complete dysfunction of the protein if they were not located on the key conserved domains. Therefore, on one hand, mutation profiling based on cfDNA sequencing could provide comprehensive diagnostic information when the cytopathology was unavailable; on the other hand, overlapping mutation should be fully considered so as to prevent false positive diagnosis.

The correlation of the mutations with clinical manifestations and prognosis was briefly analyzed in this study. In previous studies, *CD79B* mutation was considered as a risk factor for CNS progression (19, 21). While in our cohort, *CD79B* was correlated with higher IL-10 level in the vitreous (*P*=0.030, 1-β=0.740), which indicated higher tumor load in the vitreous cavity. Besides, patients carrying *CARD11* mutations tended to have earlier onset, which implied that the genotypes and corresponding clinical features could be different between age groups (22, 23). Meanwhile, we discovered that the patients with CNS involvement tended to carry multiple-site mutations in *BTG2* gene than those without (*P*=0.013, 1-β=0.772). *BTG2* (B-cell transition gene 2) is an anti-proliferation gene participating in the regulation of the cell-cycle progression, apoptosis and differentiation (24), and has been found to be mutated in B-cell malignancies (10, 20) and correlated to poor survival along with *BTG1* (25). Thus, *BTG2* mutation can be another potential biomarker for prognosis and suggest close cranial follow-up in these VRL patients.

One of the limitations was the sample size and it was inappropriate to reach the conclusion simply based on the *P* values, especially when certain mutant types were less frequently observed. Therefore, the *post-hoc* power (1-β) was calculated from the α level, frequency and distribution characteristics in order to facilitate the interpretation of the statistical outcomes. Also, the logistic model had a false positive rate of 5%. Therefore, further expansion of the cohort and corresponding follow-up length is underway to yield more confidential outcomes. Besides, the existing extraction technique of cfDNA was designed for large volumes of body fluid (e.g. blood, pleural fluid, ascites and urine). The extraction efficiency in limited amount of intraocular fluid has been improved by adding DNA fragmentation procedure to our new workflow, but the compatibility between the different kits in the workflow remains to be further verified. Another limitation was that the pathological subtypes and gene expression pattern were not further analyzed due to the lack of RNA sequencing. Thus, the capture technique of free nucleic acid combined with transcriptome sequencing remains to be further explored.

In conclusion, cfDNA sequencing of intraocular fluid can serve as liquid biopsy to facilitate the diagnosis of VRL, and can provide considerable detection rate which is no lower than that of cytopathology. High-throughput sequencing can help to distinguish different mutant types with various clinical manifestations and is of sound potential in the prognosis evaluation and individualized management of VRL.

## Data Availability Statement

All the original outcomes of cfDNA sequencing are included in the **Supplementary Material**, further inquiries can be directed to the corresponding author.

## Ethics Statement

The studies involving human participants were reviewed and approved by Institutional Review Board (IRB) and ethics committee of the Eye and ENT Hospital of Fudan University. The patients/participants provided their written informed consent to participate in this study.

## Author Contributions

QC and GX contributed equally to the research; QC, GX and JG designed and performed the research. The manuscript was drafted by JG and critically reviewed by QC and GX; the protocol of cfDNA sequencing was worked out by JG; the data were analyzed by JG and TJ; the clinical data and specimens were collected by TJ, RL, WC and SL; Cytopathologic diagnosis was performed by BP; Clinical diagnosis, treatment and enrollment were conducted by QC, GX, LW, XH and TJ; Diagnostic vitrectomy was performed by QC. All authors contributed to the article and approved the submitted version.

## Funding

National Natural Science Foundation of China (Grants no. 82171078, 81870670, 82000907). Shanghai Committee of Science and Technology (Grants no. 18411965100). Shanghai Sailing Program (Grants no. 20YF1404900). Shanghai Hospital Development Center (Grants no. SHDC2020CR2041B & SHDC2020CR5014-003). China Primary Health Care Foundation (Role of CfDNA Sequencing in Diagnosis of VRL).

## Conflict of Interest

The authors declare that the research was conducted in the absence of any commercial or financial relationships that could be construed as a potential conflict of interest.

## Publisher’s Note

All claims expressed in this article are solely those of the authors and do not necessarily represent those of their affiliated organizations, or those of the publisher, the editors and the reviewers. Any product that may be evaluated in this article, or claim that may be made by its manufacturer, is not guaranteed or endorsed by the publisher.
