# Supplementary material for: Cell-Free DNA Sequencing of Intraocular Fluid as Liquid Biopsy in the Diagnosis of Vitreoretinal Lymphoma
Source: Front Oncol. 2022 Jul 19;12:932674. doi: 10.3389/fonc.2022.932674 (PMC9343589; doi:10.3389/fonc.2022.932674)
Supplement: Supplementary file 1 [file DataSheet_1.pdf]

Supplementary table 1 List of the selected genes for cfDNA sequencing

ABCB1	BARD1	CD274	CXCR4	EPHA3	FGF12	GRM3	HLA-DQA1	JAK1	MAPK3	NBN	PARP3	PPP2R1A	RBM10	SESN2	SS18	TP53BP1
ABL1	BBC3	CD276	CYLD	EPHA5	FGF14	GSK3B	HLA-DQA2	JAK2	MAPKAP1	NCOA3	PAX3	PPP2R2A	RECQL	SESN3	SSBP1	TP63
ABRAXAS1	BCL10	CD70	CYP17A1	EPHA7	FGF19	GSTM1	HLA-DQB1	JAK3	MAX	NCOR1	PAX5	PPP4R2	RECQL4	SETBP1	STAG2	TPMT
ACVR1	BCL2	CD74	CYP19A1	EPHB1	FGF23	GSTM1	HLA-DQB2	JUN	MCL1	NEGR1	PAX8	PPP6C	REL	SETD2	STAT3	TRAF2
ACVR1B	BCL2L1	CD79A	CYP2C8	EPHB4	FGF3	GSTP1	HLA-DRA	KDM5A	MDC1	NF1	PBRM1	PRDM1	RET	SF3B1	STAT5A	TRAF7
ACYP2	BCL2L11	CD79B	CYP2D6	ERBB2	FGF4	GSTT1	HLA-DRB1	KDM5C	MDM2	NF2	PDCD1	PRDM14	RFWD2	SGK1	STAT5B	TSC1
AGO2	BCL2L2	CDA	CYSLTR2	ERBB3	FGF6	GSTT1	HLA-DRB5	KDM5D	MDM4	NFE2L2	PDCD1LG2	PREX2	RHEB	SH2B3	STK11	TSC2
AKT1	BCL6	CDC42	DAXX	ERBB4	FGFR1	H3F3A	HLA-DRB6	KDM6A	MED12	NFKBIA	PDGFRA	PRKAR1A	RHOA	SH2D1A	STK19	TSHR
AKT2	BCOR	CDC73	DAZ1	ERCC1	FGFR2	H3F3B	HLA-DRB9	KDR	MEF2B	NKX2-1	PDGFRB	PRKCI	RICTOR	SHOC2	STK40	TSPY4
AKT3	BCORL1	CDH1	DCUN1D1	ERCC1	FGFR3	H3F3C	HNF1A	KEAP1	MEN1	NKX3-1	PDK1	PRKD1	RIT1	SHQ1	SUFU	TTTY23
ALK	BCR	CDK12	DDR1	ERCC2	FGFR4	HDAC1	HOXB13	KEL	MERTK	NOTCH1	PDPK1	PRKN	RNF43	SLC28A3	SUZ12	TWIST1
ALOX12B	BIRC3	CDK4	DDR2	ERCC2	FH	HDAC2	HRAS	KIT	MET	NOTCH2	PGR	PRKY	ROS1	SLC34A2	SYCP3	TYMS
AMELY	BLM	CDK6	DICER1	ERCC3	FLCN	HGF	HSD3B1	KLC3	MGA	NOTCH3	PHF6	PTCH1	RPA1	SLCO1B1	SYK	TYRO3
AMER1	BMPR1A	CDK8	DIS3	ERCC4	FLT1	HIST1H1C	ICOSLG	KLF4	MITF	NOTCH4	PHOX2B	PTEN	RPA2	SLFN11	TAP1	U2AF1
ANKRD11	BRAF	CDKN1A	DLL3	ERCC5	FLT3	HIST1H2BD	ID3	KLHL6	MKNK1	NPM1	PIK3C2B	PTP4A1	RPA3	SLX4	TAP2	UGT1A1
ANXA2	BRCA1	CDKN1B	DNAJB1	ERF	FLT4	HIST1H3A	IDH1	KMT2A	MLH1	NQO1	PIK3C2G	PTPN11	RPA4	SMAD2	TBX3	UIMC1
APC	BRCA2	CDKN2A	DNMT1	ERG	FOXA1	HIST1H3B	IDH2	KMT2B	MLH3	NR4A3	PIK3C3	PTPRD	RPS4Y2	SMAD3	TCF3	UMPS
AR	BRCC3	CDKN2B	DNMT3A	ERRFI1	FOXL2	HIST1H3C	IFNGR1	KMT2C	MPL	NRAS	PIK3CA	PTPRO	RPS6KA4	SMAD4	TCF7L2	UPF1
ARAF	BRD4	CDKN2C	DNMT3B	ESR1	FOXO1	HIST1H3D	IFNGR2	KMT2D	MRE11	NRG1	PIK3CB	PTPRS	RPS6KB2	SMARCA4	TEK	USP9Y
ARFRP1	BRIP1	CEBPA	DOT1L	ESR1	FOXP1	HIST1H3E	IGF1	KMT5A	MSH2	NSD1	PIK3CD	PTPRT	RPTOR	SMARCB1	TEKT4	VEGFA
ARID1A	BTG1	CENPA	DPYD	ETV1	FUBP1	HIST1H3F	IGF1R	KNSTRN	MSH3	NSD2	PIK3CG	QKI	RRAGC	SMARCD1	TEKT4	VHL
ARID1B	BTG2	CEP72	DROSHA	ETV4	FUS	HIST1H3G	IGF2	KRAS	MSH6	NSD3	PIK3R1	RAB35	RRAS	SMC3	TERC	VTCN1
ARID2	BTK	CFTR	DUSP4	ETV5	FYN	HIST1H3H	IKBKE	LATS1	MSI1	NT5C2	PIK3R2	RAC1	RRAS2	SMO	TERT	WT1
ARID5B	C8orf34	CHEK1	DYNC2H1	ETV6	GABRA6	HIST1H3I	IKZF1	LATS2	MSI2	NT5C2	PIK3R3	RAC2	RRM1	SMYD3	TET1	WWTR1
ASXL1	CALR	CHEK2	E2F3	EWSR1	GATA1	HIST1H3J	IL10	LIFR	MST1	NTHL1	PIM1	RAD21	RSPO2	SNCAIP	TET2	XIAP
ASXL2	CARD11	CIC	EED	EZH1	GATA2	HIST2H3C	IL7R	LMO1	MST1R	NTRK1	PLCG2	RAD50	RTEL1	SOCS1	TFE3	XPC
ATM	CARM1	CREBBP	EGFL7	EZH2	GATA3	HIST2H3D	INHA	LRP1B	MTAP	NTRK2	PLK2	RAD51	RUNX1	SOD2	TGFBR1	XPO1
ATR	CASP8	CRKL	EGFR	EZR	GATA4	HIST3H3	INHBA	LTK	MTHFR	NTRK3	PMAIP1	RAD51B	RXRA	SOS1	TGFBR2	XRCC1
ATRX	CBFB	CRLF2	EIF1AX	FAM46C	GATA6	HLA-A	INPP4A	LYN	MTOR	NUDT15	PMS1	RAD51C	RYBP	SOX17	TIPARP	XRCC2
AURKA	CBL	CSDE1	EIF4A2	FANCA	GID4	HLA-B	INPP4B	MAF	MTRR	NUF2	PMS2	RAD51D	SDC4	SOX2	TMEM127	XRCC3
AURKB	CBLC	CSF1R	EIF4E	FANCC	GLI1	HLA-C	INPPL1	MALT1	MUS81	NUP93	PNRC1	RAD52	SDHA	SOX9	TMPRSS2	YAP1
AXIN1	CBR3	CSF3R	ELF3	FANCG	GNA11	HLA-DMA	INSR	MAP2K1	MUTYH	NUTM1	POLD1	RAD54B	SDHAF2	SPANXA2	TNFAIP3	YES1
AXIN2	CCND1	CTCF	ELOC	FANCL	GNA13	HLA-DMB	IRF1	MAP2K2	MYB	P2RY8	POLD2	RAD54L	SDHB	SPEN	TNFRSF14	ZFHX3
AXL	CCND2	CTLA4	EME1	FAS	GNAQ	HLA-DOA	IRF2	MAP2K4	MYC	PAK1	POLD3	RAF1	SDHC	SPOP	TOP1	ZFY
B2M	CCND3	CTNNA1	EMSY	FAT1	GNAS	HLA-DOB	IRF4	MAP3K1	MYCL	PAK5	POLD4	RARA	SDHD	SPRED1	TOP3A	ZNF217
BABAM1	CCNE1	CTNNB1	EP300	FBP1	GPS2	HLA-DPA1	IRS1	MAP3K13	MYCN	PALB2	POLE	RASA1	SEM1	SRC	TOP3B	ZNF423
BABAM2	CCNQ	CUL3	EPAS1	FBXW7	GREM1	HLA-DPB1	IRS2	MAP3K14	MYD88	PARP1	PPARG	RB1	SEMA3C	SRSF2	TOPBP1	ZNF703
BAP1	CD22	CUL4A	EPCAM	FGF10	GRIN2A	HLA-DPB2	ITPA	MAPK1	MYOD1	PARP2	PPM1D	RBBP8	SESN1	SRY	TP53	ZRSR2
