# Supplementary material for: Cell-Free DNA Sequencing of Intraocular Fluid as Liquid Biopsy in the Diagnosis of Vitreoretinal Lymphoma
Source: Front Oncol. 2022 Jul 19;12:932674. doi: 10.3389/fonc.2022.932674 (PMC9343589; doi:10.3389/fonc.2022.932674)
Supplement: Supplementary file 2 [file DataSheet_2.pdf]

Supplementary table 2 Overview of the mutations detected by cfDNA sequencing

Patient ID	Gene	NM	HGVSc	HGVSp	VAF
VRL1	ETV6	NM_001987.4	c.1060T>C	p.Y354H	0.495
VRL1	CREBBP	NM_004380.2	c.4337G>A	p.R1446H	0.506
VRL1	INHA	NM_002191.3	c.658C>T	p.R220W	0.516
VRL1	MYD88	NM_002468.4	c.794T>C	p.L265P	0.311
VRL1	IRF4	NM_002460.3	c.208C>G	p.L70V	0.405
VRL1	PIM1	NM_002648.3	c.367C>T	p.P123S	0.431
VRL1	PIM1	NM_002648.3	c.385C>G	p.L129V	0.510
VRL1	PIM1	NM_002648.3	c.496C>T	p.R166C	0.466
VRL1	PIM1	NM_002648.3	c.550C>T	p.L184F	0.474
VRL1	AMER1	NM_152424.3	c.1699C>A	p.Q567K	0.465
VRL1	ID3	NM_002167.4	c.313A>G	p.T105A	1.000
VRL1	MYCL	NM_001033082.2	c.1175C>G	p.T392S	1.000
VRL1	HSD3B1	NM_000862.2	c.1100C>A	p.T367N	1.000
VRL1	NOTCH2	NM_024408.3	c.57C>G	p.C19W	0.500
VRL1	PARP1	NM_001618.3	c.2866C>T	p.P956S	0.500
VRL1	TET1	NM_030625.2	c.485A>G	p.D162G	0.500
VRL1	TET1	NM_030625.2	c.1460C>T	p.S487L	0.500
VRL1	TET1	NM_030625.2	c.3053A>G	p.N1018S	0.500
VRL1	TET1	NM_030625.2	c.3369A>G	p.I1123M	0.500
VRL1	BMPR1A	NM_004329.2	c.4C>A	p.P2T	1.000
VRL1	RPS6KA4	NM_003942.2	c.2272T>G	p.S758A	1.000
VRL1	MEN1	NM_000244.3	c.1636A>G	p.T546A	1.000
VRL1	INPPL1	NM_001567.3	c.909G>C	p.K303N	0.500
VRL1	ATM	NM_000051.3	c.5948A>G	p.N1983S	1.000
VRL1	KDM5A	NM_001042603.2	c.2594T>C	p.M865T	1.000
VRL1	FGF23	NM_020638.2	c.716C>T	p.T239M	0.500
VRL1	PIK3C2G	NM_001288772.1	c.385_387del	p.P129del	1.000
VRL1	PIK3C2G	NM_001288772.1	c.437C>T	p.P146L	1.000
VRL1	SH2B3	NM_005475.2	c.784T>C	p.W262R	1.000
VRL1	HNF1A	NM_000545.6	c.1720A>G	p.S574G	1.000
VRL1	LATS2	NM_014572.2	c.1431_1436dup	p.P479_A480dup	0.500
VRL1	LATS2	NM_014572.2	c.1087G>A	p.G363S	1.000
VRL1	LATS2	NM_014572.2	c.971C>T	p.A324V	0.500
VRL1	FLT3	NM_004119.2	c.680C>T	p.T227M	1.000
VRL1	BRCA2	NM_000059.3	c.1114A>C	p.N372H	0.500
VRL1	BRCA2	NM_000059.3	c.7397T>C	p.V2466A	1.000
VRL1	DIS3	NM_014953.4	c.806A>G	p.N269S	0.500
VRL1	CUL4A	NM_001008895.2	c.1931A>G	p.K644R	0.500
VRL1	BCL2L2	NM_004050.4	c.398A>G	p.Q133R	1.000
VRL1	FOXA1	NM_004496.3	c.247G>A	p.A83T	0.500
VRL1	MLH3	NM_001040108.1	c.2476A>G	p.N826D	1.000
VRL1	TSHR	NM_000369.2	c.2181G>C	p.E727D	1.000
VRL1	TP53BP1	NM_001141980.1	c.3421A>C	p.K1141Q	1.000
VRL1	TP53BP1	NM_001141980.1	c.1249G>A	p.G417S	1.000
VRL1	TP53BP1	NM_001141980.1	c.1074C>G	p.D358E	1.000
VRL1	AXIN1	NM_003502.3	c.800T>G	p.L267R	0.500
VRL1	SLX4	NM_032444.2	c.3812C>T	p.S1271F	0.500
VRL1	CREBBP	NM_004380.2	c.1651C>A	p.L551I	0.500
VRL1	ZFHX3	NM_006885.3	c.1378G>C	p.E460Q	0.500
VRL1	ZFHX3	NM_006885.3	c.1282A>C	p.T428P	0.500
VRL1	ZFHX3	NM_006885.3	c.214T>G	p.S72A	0.500
VRL1	ANKRD11	NM_013275.5	c.6176C>A	p.P2059H	0.500
VRL1	ANKRD11	NM_013275.5	c.4912C>G	p.P1638A	0.500
VRL1	ZNF276	NM_001113525.1	c.1815G>C	p.E605D	0.500

VRL1	FANCA	NM_000135.2	c.3982A>G	p.T1328A	0.500
VRL1	FANCA	NM_000135.2	c.2426G>A	p.G809D	1.000
VRL1	FANCA	NM_000135.2	c.1927C>G	p.P643A	0.500
VRL1	FANCA	NM_000135.2	c.1501G>A	p.G501S	1.000
VRL1	FANCA	NM_000135.2	c.1235C>T	p.A412V	0.500
VRL1	FANCA	NM_000135.2	c.796A>G	p.T266A	1.000
VRL1	TP53	NM_000546.5	c.215C>G	p.P72R	1.000
VRL1	AURKB	NM_001284526.1	c.896T>C	p.M299T	1.000
VRL1	RAD51D	NM_002878.3	c.494G>A	p.R165Q	0.500
VRL1	CDK12	NM_016507.3	c.4249G>A	p.G1417R	0.500
VRL1	ERBB2	NM_004448.3	c.3508C>G	p.P1170A	0.500
VRL1	BRCA1	NM_007294.3	c.4837A>G	p.S1613G	0.500
VRL1	BRCA1	NM_007294.3	c.3548A>G	p.K1183R	0.500
VRL1	BRCA1	NM_007294.3	c.3113A>G	p.E1038G	0.500
VRL1	BRCA1	NM_007294.3	c.2612C>T	p.P871L	0.500
VRL1	RNF43	NM_017763.5	c.1252C>A	p.L418M	1.000
VRL1	RNF43	NM_017763.5	c.139A>G	p.I47V	1.000
VRL1	BRIP1	NM_032043.2	c.2755T>C	p.S919P	1.000
VRL1	AXIN2	NM_004655.3	c.148C>T	p.P50S	1.000
VRL1	TCF3	NM_001136139.2	c.1291G>A	p.G431S	1.000
VRL1	PTPRS	NM_002850.3	c.4369T>C	p.C1457R	1.000
VRL1	NOTCH3	NM_000435.2	c.6668C>T	p.A2223V	0.500
VRL1	PIK3R2	NM_005027.3	c.700A>C	p.S234R	1.000
VRL1	PIK3R2	NM_005027.3	c.937T>C	p.S313P	1.000
VRL1	KMT2B	NM_014727.2	c.3059dup	p.R1021Pfs*14	1.000
VRL1	KMT2B	NM_014727.2	c.7091A>G	p.D2364G	0.500
VRL1	AXL	NM_021913.4	c.796A>G	p.N266D	1.000
VRL1	AXL	NM_021913.4	c.1343G>T	p.W448L	0.500
VRL1	CD3EAP	NM_001297590.1	c.1516C>A	p.Q506K	0.500
VRL1	POLD1	NM_001308632.1	c.356G>A	p.R119H	0.500
VRL1	ALK	NM_004304.4	c.4587C>G	p.D1529E	0.500
VRL1	ALK	NM_004304.4	c.4472A>G	p.K1491R	0.500
VRL1	ALK	NM_004304.4	c.4381A>G	p.I1461V	1.000
VRL1	EPCAM	NM_002354.2	c.344T>C	p.M115T	1.000
VRL1	LRP1B	NM_018557.2	c.143A>G	p.Q48R	0.500
VRL1	CTLA4	NM_005214.4	c.49A>G	p.T17A	1.000
VRL1	BARD1	NM_000465.3	c.1134G>C	p.R378S	1.000
VRL1	BARD1	NM_000465.3	c.70C>T	p.P24S	1.000
VRL1	CUL3	NM_001257198.1	c.1717G>A	p.V573I	0.500
VRL1	PDCD1	NM_005018.2	c.644C>T	p.A215V	1.000
VRL1	ASXL1	NM_015338.5	c.2444T>C	p.L815P	1.000
VRL1	PTPRT	NM_133170.3	c.85G>C	p.A29P	1.000
VRL1	AURKA	NM_003600.3	c.169A>G	p.I57V	0.500
VRL1	AURKA	NM_003600.3	c.91T>A	p.F31I	0.500
VRL1	RTEL1	NM_001283009.1	c.3126A>C	p.Q1042H	0.500
VRL1	TMPRSS2	NM_005656.3	c.478G>A	p.V160M	0.500
VRL1	ICOSLG	NM_001283050.1	c.382G>A	p.V128I	1.000
VRL1	EP300	NM_001429.3	c.2989A>G	p.I997V	0.500
VRL1	MLH1	NM_000249.3	c.1151T>A	p.V384D	0.500
VRL1	SETD2	NM_014159.6	c.5885C>T	p.P1962L	0.500
VRL1	EPHA3	NM_005233.5	c.2741G>A	p.R914H	0.500
VRL1	EPHA3	NM_005233.5	c.2770T>C	p.W924R	0.500
VRL1	GATA2	NM_032638.4	c.490G>A	p.A164T	0.500
VRL1	ATR	NM_001184.3	c.632T>C	p.M211T	1.000
VRL1	EPHA5	NM_001281765.2	c.2879A>G	p.H960R	0.500
VRL1	ABRAXAS1	NM_139076.2	c.1042G>A	p.A348T	0.500

VRL1	TET2	NM_001127208.2	c.652G>A	p.V218M	0.500
VRL1	FAT1	NM_005245.3	c.12177G>C	p.K4059N	1.000
VRL1	FAT1	NM_005245.3	c.11216C>T	p.A3739V	0.500
VRL1	FAT1	NM_005245.3	c.10660T>G	p.S3554A	0.500
VRL1	FAT1	NM_005245.3	c.10001T>C	p.V3334A	0.500
VRL1	FAT1	NM_005245.3	c.8798A>C	p.Q2933P	0.500
VRL1	FAT1	NM_005245.3	c.4985A>G	p.N1662S	0.500
VRL1	FAT1	NM_005245.3	c.3818A>G	p.H1273R	0.500
VRL1	FAT1	NM_005245.3	c.3190A>G	p.R1064G	0.500
VRL1	FAT1	NM_005245.3	c.2584G>C	p.V862L	0.500
VRL1	FAT1	NM_005245.3	c.1842C>G	p.F614L	0.500
VRL1	FAT1	NM_005245.3	c.1444G>A	p.V482I	0.500
VRL1	FAT1	NM_005245.3	c.1212T>G	p.S404R	0.500
VRL1	DROSHA	NM_013235.4	c.962C>T	p.S321L	0.500
VRL1	RICTOR	NM_001285439.1	c.2510C>T	p.S837F	0.500
VRL1	MAP3K1	NM_005921.1	c.2716G>A	p.V906I	1.000
VRL1	MAP3K1	NM_005921.1	c.2845_2847del	p.T949del	0.500
VRL1	MSH3	NM_002439.4	c.199_207del	p.P67_P69del	0.500
VRL1	MSH3	NM_002439.4	c.235A>G	p.I79V	1.000
VRL1	MSH3	NM_002439.4	c.2846A>G	p.Q949R	1.000
VRL1	MSH3	NM_002439.4	c.3133G>A	p.A1045T	1.000
VRL1	APC	NM_000038.5	c.5465T>A	p.V1822D	1.000
VRL1	CSF1R	NM_005211.3	c.1085A>G	p.H362R	1.000
VRL1	FGFR4	NM_002011.4	c.28G>A	p.V10I	1.000
VRL1	FGFR4	NM_002011.4	c.407C>T	p.P136L	1.000
VRL1	NSD1	NM_022455.4	c.1840G>T	p.V614L	0.500
VRL1	NSD1	NM_022455.4	c.2176T>C	p.S726P	0.500
VRL1	FLT4	NM_182925.4	c.2670C>G	p.H890Q	0.500
VRL1	HIST1H1C	NM_005319.3	c.53C>T	p.A18V	1.000
VRL1	NOTCH4	NM_004557.3	c.958A>G	p.T320A	1.000
VRL1	NOTCH4	NM_004557.3	c.36_47del	p.L13_L16del	1.000
VRL1	CDKN1A	NM_001291549.1	c.195C>A	p.S65R	0.500
VRL1	CCND3	NM_001760.4	c.775T>G	p.S259A	0.500
VRL1	EPHA7	NM_004440.3	c.832C>T	p.P278S	0.500
VRL1	EPHA7	NM_004440.3	c.412A>G	p.I138V	0.500
VRL1	PRKN	NM_004562.2	c.1138G>C	p.V380L	0.500
VRL1	PMS2	NM_000535.6	c.1621A>G	p.K541E	1.000
VRL1	PMS2	NM_000535.6	c.1408C>T	p.P470S	0.500
VRL1	ETV1	NM_004956.4	c.298A>G	p.S100G	0.500
VRL1	EGFR	NM_005228.3	c.1562G>A	p.R521K	0.500
VRL1	MET	NM_000245.3	c.1124A>G	p.N375S	0.500
VRL1	KMT2C	NM_170606.2	c.2512G>A	p.G838S	0.500
VRL1	KMT2C	NM_170606.2	c.2447dup	p.Y816*	0.500
VRL1	SOX17	NM_022454.3	c.595A>T	p.M199L	0.500
VRL1	PREX2	NM_024870.3	c.4463C>T	p.S1488L	0.500
VRL1	NBN	NM_002485.4	c.553G>C	p.E185Q	0.500
VRL1	RECQL4	NM_004260.3	c.3014G>A	p.R1005Q	0.500
VRL1	RECQL4	NM_004260.3	c.801G>C	p.E267D	0.500
VRL1	RECQL4	NM_004260.3	c.274T>C	p.S92P	1.000
VRL1	PDCD1LG2	NM_025239.3	c.686T>C	p.F229S	1.000
VRL1	PTCH1	NM_000264.3	c.3944C>T	p.P1315L	1.000
VRL1	TSC1	NM_000368.4	c.965T>C	p.M322T	0.500
VRL1	AR	NM_000044.3	c.234_239dup	p.Q79_Q80dup	0.500
VRL1	BCORL1	NM_001184772.2	c.331T>C	p.F111L	1.000
VRL1	CCNQ	NM_152274.4	c.16dup	p.G7Rfs*51	1.000
VRL2	ATM	NM_000051.3	c.171G>A	p.W57*	0.463

VRL2	MYD88	NM_002468.5	c.794T>C	p.L265P	0.404
VRL2	ETV6	NM_001987.4	c.1196G>A	p.R399H	0.361
VRL2	MLH1	NM_000249.3	c.1171C>T	p.Q391*	0.057
VRL2	MSH2	NM_000251.2	c.2302G>A	p.E768K	0.046
VRL2	NOTCH3	NM_000435.2	c.4039G>C	p.G1347R	0.104
VRL2	IGF1R	NM_000875.4	c.2524C>T	p.P842S	0.439
VRL2	VEGFA	NM_001025366.2	c.131G>A	p.G44E	0.407
VRL2	MGA	NM_001080541.2	c.1547A>G	p.N516S	0.554
VRL2	BCOR	NM_001123385.1	c.599C>T	p.T200M	0.467
VRL2	TSHR	NM_001142626.2	c.812G>A	p.S271N	0.487
VRL2	FGFR2	NM_001144919.1	c.747T>G	p.Y249*	0.512
VRL2	PIM1	NM_001243186	c.760G>A	p.V254M	0.523
VRL2	KLF4	NM_001314052	c.1292C>T	p.T431I	0.518
VRL2	CCND3	NM_001760.4	c.128C>T	p.S43F	0.447
VRL2	H3F3A	NM_002107.4	c.394C>T	p.R132C	0.133
VRL2	KDR	NM_002253.3	c.2593G>A	p.V865I	0.390
VRL2	MYD88	NM_002468.5	c.814G>C	p.A272P	0.410
VRL2	PTPRD	NM_002839.3	c.991G>A	p.V331M	0.396
VRL2	TOP1	NM_003286.3	c.337C>T	p.P113S	0.500
VRL2	TP73	NM_005427.3	c.166G>A	p.G56S	0.510
VRL2	LNPEP	NM_005575.2	c.82G>A	p.V28M	0.448
VRL2	TP53BP1	NM_005657.3	c.3740G>A	p.R1247H	0.034
VRL2	RANBP2	NM_006267.4	c.5300T>C	p.I1767T	0.060
VRL2	LZTR1	NM_006767.3	c.1762C>T	p.R588W	0.518
VRL2	LZTR1	NM_006767.3	c.2368C>T	p.R790W	0.417
VRL2	PTPRT	NM_007050.5	c.1054C>T	p.P352S	0.325
VRL2	ASXL1	NM_015338.5	c.3710C>T	p.S1237F	0.432
VRL2	LRP1B	NM_018557.2	c.10337C>T	p.T3446M	0.407
VRL2	STAT3	NM_139276.2	c.1679C>T	p.S560F	0.025
VRL2	ARID2	NM_152641.3	c.5206C>T	p.H1736Y	0.033
VRL2	PIK3R1	NM_181523.2	c.346C>T	p.P116S	0.451
VRL2	NCOA3	NM_181659.2	c.3937C>T	p.P1313S	0.790
VRL2	FGFR4	NM_213647.2	c.1209_1212delCGCC	p.A404fs	0.075
VRL2	APC	NM_000038	c.5465T>A	p.V1822D	0.512
VRL2	ATM	NM_000051	c.5948A>G	p.N1983S	1.000
VRL2	ATR	NM_001184	c.632T>C	p.M211T	0.458
VRL2	AXIN2	NM_004655	c.148C>T	p.P50S	0.358
VRL2	BARD1	NM_000465	c.70C>T	p.P24S	0.487
VRL2	BARD1	NM_000465	c.1519G>A	p.V507M	0.486
VRL2	BARD1	NM_000465	c.1134G>C	p.R378S	0.485
VRL2	BLM	NM_000057	c.893C>T	p.T298M	0.467
VRL2	BMPR1A	NM_004329	c.4C>A	p.P2T	0.402
VRL2	BRCA1	NM_007294	c.3113A>G	p.E1038G	0.483
VRL2	BRCA1	NM_007294	c.2612C>T	p.P871L	0.496
VRL2	BRCA1	NM_007294	c.3548A>G	p.K1183R	0.460
VRL2	BRCA1	NM_007294	c.2083G>A	p.D695N	0.489
VRL2	BRCA1	NM_007294	c.4837A>G	p.S1613G	0.442
VRL2	BRCA2	NM_000059	c.7397T>C	p.V2466A	1.000
VRL2	BRIP1	NM_032043	c.2755T>C	p.S919P	0.517
VRL2	CHEK1	NM_001114121	c.1411A>G	p.I471V	1.000
VRL2	EPCAM	NM_002354	c.344T>C	p.M115T	1.000
VRL2	HNF1A	NM_000545	c.1720A>G	p.S574G	1.000
VRL2	HNF1A	NM_000545	c.1460G>A	p.S487N	1.000
VRL2	MUTYH	NM_012222	c.1005G>C	p.Q335H	1.000
VRL2	NBN	NM_002485	c.553G>C	p.E185Q	1.000
VRL2	PALB2	NM_024675	c.1676A>G	p.Q559R	0.548

VRL2	PMS2	NM_000535	c.2570G>C	p.G857A	0.936
VRL2	PMS2	NM_000535	c.1621A>G	p.K541E	1.000
VRL2	PMS2	NM_000535	c.1408C>T	p.P470S	1.000
VRL2	POLD1	NM_002691	c.356G>A	p.R119H	0.394
VRL2	TP53	NM_000546	c.215C>G	p.P72R	0.560
VRL3	BTG2	NM_006763.2	c.102G>C	p.R34S	0.307
VRL3	BTG2	NM_006763.2	c.202A>T	p.I68F	0.320
VRL3	ETV6	NM_001987.4	c.26G>A	p.S9N	0.302
VRL3	PIK3CB	NM_006219.2	c.1095T>G	p.C365W	0.074
VRL3	IRF4	NM_002460.3	c.70del	p.L24Sfs*5	0.148
VRL3	IRF4	NM_002460.3	c.81G>A	p.W27*	0.007
VRL3	IRF4	NM_002460.3	c.162G>A	p.W54*	0.016
VRL3	IRF4	NM_002460.3	c.178C>T	p.Q60*	0.045
VRL3	QKI	NM_006775.2	c.477T>G	p.D159E	0.020
VRL3	MYC	NM_002467.4	c.357G>C	p.E119D	0.142
VRL3	SPEN	NM_015001.2	c.2909C>T	p.A970V	1.000
VRL3	SPEN	NM_015001.2	c.3272T>C	p.L1091P	1.000
VRL3	SPEN	NM_015001.2	c.7078A>G	p.N2360D	1.000
VRL3	ID3	NM_002167.4	c.313A>G	p.T105A	1.000
VRL3	MYCL	NM_001033082.2	c.1175C>G	p.T392S	1.000
VRL3	MUTYH	NM_001128425.1	c.1014G>C	p.Q338H	0.500
VRL3	MUTYH	NM_001128425.1	c.874C>G	p.P292A	0.500
VRL3	BCL10	NM_003921.4	c.13G>T	p.A5S	0.500
VRL3	HSD3B1	NM_000862.2	c.1100C>A	p.T367N	1.000
VRL3	NOTCH2	NM_024408.3	c.57C>G	p.C19W	0.500
VRL3	PARP1	NM_001618.3	c.2819A>G	p.K940R	1.000
VRL3	RET	NM_020975.4	c.2071G>A	p.G691S	0.500
VRL3	RET	NM_020975.4	c.2488G>A	p.G830R	0.500
VRL3	TET1	NM_030625.2	c.485A>G	p.D162G	0.500
VRL3	TET1	NM_030625.2	c.577T>A	p.S193T	0.500
VRL3	TET1	NM_030625.2	c.767C>T	p.A256V	0.500
VRL3	TET1	NM_030625.2	c.3369A>G	p.I1123M	1.000
VRL3	BMPR1A	NM_004329.2	c.4C>A	p.P2T	1.000
VRL3	MEN1	NM_000244.3	c.1636A>G	p.T546A	0.500
VRL3	BIRC3	NM_001165.4	c.779A>G	p.K260R	0.500
VRL3	ATM	NM_000051.3	c.5948A>G	p.N1983S	1.000
VRL3	KDM5A	NM_001042603.2	c.2594T>C	p.M865T	1.000
VRL3	GLI1	NM_005269.2	c.1595G>A	p.R532H	0.500
VRL3	GLI1	NM_005269.2	c.2798G>A	p.G933D	0.500
VRL3	GLI1	NM_005269.2	c.3298G>C	p.E1100Q	0.500
VRL3	SH2B3	NM_005475.2	c.784T>C	p.W262R	1.000
VRL3	HNF1A	NM_000545.6	c.1460G>A	p.S487N	0.500
VRL3	HNF1A	NM_000545.6	c.1720A>G	p.S574G	1.000
VRL3	LATS2	NM_014572.2	c.1431_1436dup	p.P479_A480dup	0.500
VRL3	LATS2	NM_014572.2	c.1087G>A	p.G363S	1.000
VRL3	LATS2	NM_014572.2	c.971C>T	p.A324V	1.000
VRL3	FLT3	NM_004119.2	c.20A>G	p.D7G	1.000
VRL3	BRCA2	NM_000059.3	c.865A>C	p.N289H	0.500
VRL3	BRCA2	NM_000059.3	c.2971A>G	p.N991D	0.500
VRL3	BRCA2	NM_000059.3	c.7397T>C	p.V2466A	1.000
VRL3	CYSLTR2	NM_020377.3	c.338T>C	p.I113T	0.500
VRL3	DIS3	NM_014953.4	c.806A>G	p.N269S	0.500
VRL3	BCL2L2	NM_004050.4	c.398A>G	p.Q133R	1.000
VRL3	FOXA1	NM_004496.3	c.247G>A	p.A83T	0.500
VRL3	MLH3	NM_001040108.1	c.2825C>T	p.T942I	0.500
VRL3	MLH3	NM_001040108.1	c.2476A>G	p.N826D	1.000

VRL3	TSHR	NM_000369.2	c.2181G>C	p.E727D	1.000
VRL3	TP53BP1	NM_001141980.1	c.3421A>C	p.K1141Q	1.000
VRL3	TP53BP1	NM_001141980.1	c.1249G>A	p.G417S	1.000
VRL3	TP53BP1	NM_001141980.1	c.1074C>G	p.D358E	1.000
VRL3	SLX4	NM_032444.2	c.3365C>T	p.P1122L	0.500
VRL3	ZFHX3	NM_006885.3	c.1378G>C	p.E460Q	0.500
VRL3	ZFHX3	NM_006885.3	c.214T>G	p.S72A	0.500
VRL3	ANKRD11	NM_013275.5	c.6176C>A	p.P2059H	1.000
VRL3	ANKRD11	NM_013275.5	c.4912C>G	p.P1638A	1.000
VRL3	FANCA	NM_000135.2	c.2426G>A	p.G809D	1.000
VRL3	FANCA	NM_000135.2	c.1501G>A	p.G501S	0.500
VRL3	FANCA	NM_000135.2	c.796A>G	p.T266A	1.000
VRL3	TP53	NM_000546.5	c.215C>G	p.P72R	0.500
VRL3	AURKB	NM_001284526.1	c.896T>C	p.M299T	1.000
VRL3	ERBB2	NM_004448.3	c.3508C>G	p.P1170A	0.500
VRL3	BRCA1	NM_007294.3	c.4837A>G	p.S1613G	1.000
VRL3	BRCA1	NM_007294.3	c.3548A>G	p.K1183R	1.000
VRL3	BRCA1	NM_007294.3	c.3113A>G	p.E1038G	1.000
VRL3	BRCA1	NM_007294.3	c.2612C>T	p.P871L	1.000
VRL3	RNF43	NM_017763.5	c.1252C>A	p.L418M	0.500
VRL3	RNF43	NM_017763.5	c.662G>A	p.R221Q	0.500
VRL3	RNF43	NM_017763.5	c.350G>A	p.R117H	0.500
VRL3	RNF43	NM_017763.5	c.139A>G	p.I47V	0.500
VRL3	BRIP1	NM_032043.2	c.2755T>C	p.S919P	1.000
VRL3	AXIN2	NM_004655.3	c.148C>T	p.P50S	1.000
VRL3	TCF3	NM_001136139.2	c.1475C>T	p.A492V	1.000
VRL3	DOT1L	NM_032482.2	c.4156G>A	p.G1386S	0.500
VRL3	PTPRS	NM_002850.3	c.4369T>C	p.C1457R	1.000
VRL3	DNMT1	NM_001130823.2	c.979A>G	p.I327V	1.000
VRL3	DNMT1	NM_001130823.2	c.358G>C	p.V120L	0.500
VRL3	DNMT1	NM_001130823.2	c.290A>G	p.H97R	0.500
VRL3	NOTCH3	NM_000435.2	c.6668C>T	p.A2223V	0.500
VRL3	PIK3R2	NM_005027.3	c.700A>C	p.S234R	1.000
VRL3	PIK3R2	NM_005027.3	c.937T>C	p.S313P	1.000
VRL3	CEBPA	NM_004364.4	c.584_589dup	p.H195_P196dup	0.500
VRL3	CD22	NM_001771.3	c.2234G>A	p.G745D	0.500
VRL3	KMT2B	NM_014727.2	c.3059dup	p.R1021Pfs*14	1.000
VRL3	KMT2B	NM_014727.2	c.5486C>T	p.P1829L	0.500
VRL3	KMT2B	NM_014727.2	c.7091A>G	p.D2364G	0.500
VRL3	AXL	NM_021913.4	c.796A>G	p.N266D	1.000
VRL3	CD3EAP	NM_001297590.1	c.1516C>A	p.Q506K	0.500
VRL3	ERCC1	NM_202001.2	c.226T>C	p.C76R	0.500
VRL3	ALK	NM_004304.4	c.4587C>G	p.D1529E	1.000
VRL3	ALK	NM_004304.4	c.4472A>G	p.K1491R	1.000
VRL3	ALK	NM_004304.4	c.4381A>G	p.I1461V	1.000
VRL3	EPCAM	NM_002354.2	c.344T>C	p.M115T	1.000
VRL3	MSH6	NM_000179.2	c.116G>A	p.G39E	1.000
VRL3	MERTK	NM_006343.2	c.353G>A	p.S118N	0.500
VRL3	MERTK	NM_006343.2	c.1397G>A	p.R466K	0.500
VRL3	MERTK	NM_006343.2	c.1552A>G	p.I518V	0.500
VRL3	LRP1B	NM_018557.2	c.143A>G	p.Q48R	0.500
VRL3	CTLA4	NM_005214.4	c.49A>G	p.T17A	1.000
VRL3	IDH1	NM_005896.3	c.1078G>C	p.E360Q	0.500
VRL3	BARD1	NM_000465.3	c.1134G>C	p.R378S	0.500
VRL3	BARD1	NM_000465.3	c.70C>T	p.P24S	0.500
VRL3	IRS1	NM_005544.2	c.3409G>A	p.D1137N	0.500

VRL3	ASXL1	NM_015338.5	c.2444T>C	p.L815P	1.000
VRL3	PTPRT	NM_133170.3	c.85G>C	p.A29P	0.500
VRL3	AURKA	NM_003600.3	c.169A>G	p.I57V	1.000
VRL3	AURKA	NM_003600.3	c.91T>A	p.F31I	0.500
VRL3	RTEL1	NM_001283009.1	c.3126A>C	p.Q1042H	0.500
VRL3	TMPRSS2	NM_005656.3	c.478G>A	p.V160M	0.500
VRL3	MLH1	NM_000249.3	c.1348G>T	p.D450Y	0.500
VRL3	SETD2	NM_014159.6	c.5885C>T	p.P1962L	0.500
VRL3	GATA2	NM_032638.4	c.490G>A	p.A164T	0.500
VRL3	ATR	NM_001184.3	c.632T>C	p.M211T	1.000
VRL3	ATR	NM_001184.3	c.325C>T	p.R109W	1.000
VRL3	FGFR3	NM_000142.4	c.193G>A	p.G65R	0.500
VRL3	KDR	NM_002253.2	c.1416A>T	p.Q472H	0.500
VRL3	KDR	NM_002253.2	c.364A>G	p.R122G	0.500
VRL3	TET2	NM_001127208.2	c.86C>G	p.P29R	1.000
VRL3	FAT1	NM_005245.3	c.13652C>G	p.A4551G	0.500
VRL3	FAT1	NM_005245.3	c.12177G>C	p.K4059N	1.000
VRL3	FAT1	NM_005245.3	c.11177T>A	p.I3726N	0.500
VRL3	FAT1	NM_005245.3	c.10660T>G	p.S3554A	0.500
VRL3	FAT1	NM_005245.3	c.8798A>C	p.Q2933P	0.500
VRL3	FAT1	NM_005245.3	c.7105A>C	p.T2369P	0.500
VRL3	FAT1	NM_005245.3	c.6822C>G	p.D2274E	0.500
VRL3	FAT1	NM_005245.3	c.4690G>A	p.A1564T	0.500
VRL3	FAT1	NM_005245.3	c.3818A>G	p.H1273R	0.500
VRL3	FAT1	NM_005245.3	c.3190A>G	p.R1064G	1.000
VRL3	FAT1	NM_005245.3	c.2584G>C	p.V862L	1.000
VRL3	FAT1	NM_005245.3	c.1842C>G	p.F614L	1.000
VRL3	FAT1	NM_005245.3	c.1327A>G	p.R443G	0.500
VRL3	FAT1	NM_005245.3	c.392C>T	p.A131V	1.000
VRL3	RICTOR	NM_001285439.1	c.2510C>T	p.S837F	1.000
VRL3	MSH3	NM_002439.4	c.199_207del	p.P67_P69del	0.500
VRL3	MSH3	NM_002439.4	c.235A>G	p.I79V	1.000
VRL3	MSH3	NM_002439.4	c.2846A>G	p.Q949R	1.000
VRL3	MSH3	NM_002439.4	c.3133G>A	p.A1045T	1.000
VRL3	APC	NM_000038.5	c.5465T>A	p.V1822D	1.000
VRL3	CSF1R	NM_005211.3	c.1085A>G	p.H362R	1.000
VRL3	PDGFRB	NM_002609.3	c.85A>T	p.I29F	0.500
VRL3	FGFR4	NM_002011.4	c.28G>A	p.V10I	0.500
VRL3	FGFR4	NM_002011.4	c.407C>T	p.P136L	1.000
VRL3	FGFR4	NM_002011.4	c.1162G>A	p.G388R	0.500
VRL3	NSD1	NM_022455.4	c.6750G>A	p.M2250I	0.500
VRL3	NSD1	NM_022455.4	c.6782T>C	p.M2261T	0.500
VRL3	NSD1	NM_022455.4	c.7636G>A	p.A2546T	0.500
VRL3	FLT4	NM_182925.4	c.2670C>G	p.H890Q	1.000
VRL3	IRF4	NM_002460.3	c.208C>G	p.L70V	0.500
VRL3	HIST1H1C	NM_005319.3	c.53C>T	p.A18V	1.000
VRL3	NOTCH4	NM_004557.3	c.349A>C	p.K117Q	1.000
VRL3	NOTCH4	NM_004557.3	c.45_47del	p.L16del	0.500
VRL3	TAP1	NM_000593.5	c.2122C>T	p.R708*	1.000
VRL3	CDKN1A	NM_001291549.1	c.83A>G	p.D28G	1.000
VRL3	CDKN1A	NM_001291549.1	c.195C>A	p.S65R	1.000
VRL3	PIM1	NM_002648.3	c.97C>T	p.P33S	0.500
VRL3	PIM1	NM_002648.3	c.316C>G	p.L106V	0.500
VRL3	CCND3	NM_001760.4	c.775T>G	p.S259A	0.500
VRL3	ROS1	NM_002944.2	c.6686C>G	p.S2229C	0.500
VRL3	ROS1	NM_002944.2	c.6682A>C	p.K2228Q	0.500

VRL3	ROS1	NM_002944.2	c.6637G>A	p.D2213N	0.500
VRL3	ROS1	NM_002944.2	c.1611A>G	p.I537M	0.500
VRL3	ROS1	NM_002944.2	c.433A>C	p.T145P	0.500
VRL3	SGK1	NM_001143676.1	c.143C>T	p.S48F	0.500
VRL3	PRKN	NM_004562.2	c.1138G>C	p.V380L	0.500
VRL3	CARD11	NM_032415.5	c.645G>T	p.K215N	0.500
VRL3	PMS2	NM_000535.6	c.1621A>G	p.K541E	1.000
VRL3	PMS2	NM_000535.6	c.1454C>A	p.T485K	0.500
VRL3	PMS2	NM_000535.6	c.1408C>T	p.P470S	0.500
VRL3	ETV1	NM_004956.4	c.298A>G	p.S100G	0.500
VRL3	EGFR	NM_005228.3	c.1562G>A	p.R521K	0.500
VRL3	EZH2	NM_004456.4	c.553G>C	p.D185H	0.500
VRL3	KMT2C	NM_170606.2	c.2963G>T	p.C988F	0.500
VRL3	KMT2C	NM_170606.2	c.2512G>A	p.G838S	0.500
VRL3	KMT2C	NM_170606.2	c.2447dup	p.Y816*	0.500
VRL3	PREX2	NM_024870.3	c.4180C>T	p.R1394W	0.500
VRL3	NBN	NM_002485.4	c.553G>C	p.E185Q	1.000
VRL3	RECQL4	NM_004260.3	c.3014G>A	p.R1005Q	1.000
VRL3	RECQL4	NM_004260.3	c.274T>C	p.S92P	1.000
VRL3	PDCD1LG2	NM_025239.3	c.686T>C	p.F229S	1.000
VRL3	PDCD1LG2	NM_025239.3	c.722T>C	p.I241T	1.000
VRL3	PAX5	NM_016734.2	c.11A>T	p.E4V	0.500
VRL3	PTCH1	NM_000264.3	c.3944C>T	p.P1315L	1.000
VRL3	PTCH1	NM_000264.3	c.2222C>T	p.A741V	0.500
VRL3	AR	NM_000044.3	c.234_239del	p.Q79_Q80del	0.500
VRL3	BCORL1	NM_001184772.2	c.331T>C	p.F111L	1.000
VRL3	CCNQ	NM_152274.4	c.16dup	p.G7Rfs*51	1.000
VRL4	KMT2D	NM_003482.3	c.12863G>T	p.R4288L	0.303
VRL4	IGF1R	NM_000875.4	c.3251G>A	p.R1084Q	0.458
VRL4	CD79B	NM_001039933.2	c.590A>C	p.Y197S	0.486
VRL4	ALK	NM_004304.4	c.1529G>A	p.R510Q	0.459
VRL4	MYD88	NM_002468.4	c.794T>C	p.L265P	0.496
VRL4	WWTR1	NM_015472.4	c.323A>G	p.Q108R	0.492
VRL4	HIST1H3G	NM_003534.2	c.347A>G	p.K116R	0.424
VRL4	TAP2	NM_000544.3	c.752C>A	p.S251*	0.457
VRL4	PIM1	NM_002648.3	c.68C>G	p.T23S	0.487
VRL4	PIM1	NM_002648.3	c.134G>A	p.G45D	0.464
VRL4	PIM1	NM_002648.3	c.549G>C	p.K183N	0.474
VRL4	PRDM1	NM_001198.3	c.291G>C	p.E97D	0.930
VRL4	PREX2	NM_024870.3	c.1769T>C	p.I590T	0.456
VRL4	MYC	NM_002467.4	c.132G>C	p.E44D	0.940
VRL4	ID3	NM_002167.4	c.313A>G	p.T105A	1.000
VRL4	MYCL	NM_001033082.2	c.1175C>G	p.T392S	1.000
VRL4	FAM46C	NM_017709.3	c.201C>G	p.H67Q	0.500
VRL4	HSD3B1	NM_000862.2	c.1100C>A	p.T367N	1.000
VRL4	NOTCH2	NM_024408.3	c.57C>G	p.C19W	0.500
VRL4	PARP1	NM_001618.3	c.2819A>G	p.K940R	0.500
VRL4	TET1	NM_030625.2	c.485A>G	p.D162G	0.500
VRL4	TET1	NM_030625.2	c.3369A>G	p.I1123M	1.000
VRL4	BMPR1A	NM_004329.2	c.4C>A	p.P2T	0.500
VRL4	TCF7L2	NM_001146274.1	c.1447C>A	p.P483T	0.500
VRL4	MEN1	NM_000244.3	c.1636A>G	p.T546A	0.500
VRL4	ATM	NM_000051.3	c.5948A>G	p.N1983S	1.000
VRL4	SDHD	NM_003002.3	c.354T>G	p.D118E	0.500
VRL4	KMT2A	NM_001197104.1	c.9391G>A	p.G3131S	0.500
VRL4	KDM5A	NM_001042603.2	c.2594T>C	p.M865T	0.500

VRL4	H3F3C	NM_001013699.2	c.116A>C	p.H39P	0.500
VRL4	ARID2	NM_152641.2	c.4300G>T	p.A1434S	0.500
VRL4	KMT2D	NM_003482.3	c.7144C>T	p.P2382S	0.500
VRL4	ERBB3	NM_001982.3	c.1909A>T	p.I637F	0.500
VRL4	GLI1	NM_005269.2	c.3035G>T	p.G1012V	0.500
VRL4	GLI1	NM_005269.2	c.3298G>C	p.E1100Q	0.500
VRL4	SH2B3	NM_005475.2	c.784T>C	p.W262R	1.000
VRL4	HNF1A	NM_000545.6	c.79A>C	p.I27L	1.000
VRL4	HNF1A	NM_000545.6	c.1460G>A	p.S487N	1.000
VRL4	HNF1A	NM_000545.6	c.1720A>G	p.S574G	1.000
VRL4	LATS2	NM_014572.2	c.1431_1436dup	p.P479_A480dup	0.500
VRL4	LATS2	NM_014572.2	c.1087G>A	p.G363S	1.000
VRL4	LATS2	NM_014572.2	c.971C>T	p.A324V	0.500
VRL4	FLT3	NM_004119.2	c.680C>T	p.T227M	0.500
VRL4	FLT3	NM_004119.2	c.20A>G	p.D7G	0.500
VRL4	BRCA2	NM_000059.3	c.7397T>C	p.V2466A	1.000
VRL4	DIS3	NM_014953.4	c.806A>G	p.N269S	0.500
VRL4	IRS2	NM_003749.2	c.3170G>A	p.G1057D	0.500
VRL4	BCL2L2	NM_004050.4	c.398A>G	p.Q133R	1.000
VRL4	FOXA1	NM_004496.3	c.247G>A	p.A83T	0.500
VRL4	MLH3	NM_001040108.1	c.2531C>T	p.P844L	0.500
VRL4	MLH3	NM_001040108.1	c.2476A>G	p.N826D	1.000
VRL4	TSHR	NM_000369.2	c.2181G>C	p.E727D	1.000
VRL4	LTK	NM_002344.5	c.125G>A	p.R42Q	0.500
VRL4	TP53BP1	NM_001141980.1	c.3421A>C	p.K1141Q	0.500
VRL4	TP53BP1	NM_001141980.1	c.1249G>A	p.G417S	0.500
VRL4	TP53BP1	NM_001141980.1	c.1074C>G	p.D358E	0.500
VRL4	SLX4	NM_032444.2	c.3365C>T	p.P1122L	0.500
VRL4	PALB2	NM_024675.3	c.1676A>G	p.Q559R	1.000
VRL4	ZFHX3	NM_006885.3	c.10445G>T	p.S3482I	0.500
VRL4	ZFHX3	NM_006885.3	c.214T>G	p.S72A	0.500
VRL4	ANKRD11	NM_013275.5	c.2912C>T	p.A971V	0.500
VRL4	FANCA	NM_000135.2	c.2426G>A	p.G809D	1.000
VRL4	FANCA	NM_000135.2	c.1501G>A	p.G501S	1.000
VRL4	FANCA	NM_000135.2	c.796A>G	p.T266A	1.000
VRL4	AURKB	NM_001284526.1	c.896T>C	p.M299T	0.500
VRL4	ERBB2	NM_004448.3	c.3508C>G	p.P1170A	0.500
VRL4	RNF43	NM_017763.5	c.1252C>A	p.L418M	0.500
VRL4	RNF43	NM_017763.5	c.1093G>A	p.A365T	0.500
VRL4	RNF43	NM_017763.5	c.578C>T	p.A193V	0.500
VRL4	RNF43	NM_017763.5	c.350G>A	p.R117H	0.500
VRL4	RNF43	NM_017763.5	c.139A>G	p.I47V	0.500
VRL4	BRIP1	NM_032043.2	c.2755T>C	p.S919P	1.000
VRL4	AXIN2	NM_004655.3	c.148C>T	p.P50S	0.500
VRL4	TCF3	NM_001136139.2	c.1291G>A	p.G431S	0.500
VRL4	DOT1L	NM_032482.2	c.4252G>C	p.V1418L	1.000
VRL4	PTPRS	NM_002850.3	c.4369T>C	p.C1457R	1.000
VRL4	DNMT1	NM_001130823.2	c.979A>G	p.I327V	0.500
VRL4	DNMT1	NM_001130823.2	c.290A>G	p.H97R	0.500
VRL4	NOTCH3	NM_000435.2	c.6668C>T	p.A2223V	1.000
VRL4	PIK3R2	NM_005027.3	c.700A>C	p.S234R	1.000
VRL4	PIK3R2	NM_005027.3	c.937T>C	p.S313P	1.000
VRL4	CEBPA	NM_004364.4	c.584_589dup	p.H195_P196dup	0.500
VRL4	KMT2B	NM_014727.2	c.3059dup	p.R1021Pfs*14	1.000
VRL4	KMT2B	NM_014727.2	c.5486C>T	p.P1829L	0.500
VRL4	KMT2B	NM_014727.2	c.7091A>G	p.D2364G	0.500

VRL4	AXL	NM_021913.4	c.796A>G	p.N266D	1.000
VRL4	ALK	NM_004304.4	c.4587C>G	p.D1529E	0.500
VRL4	ALK	NM_004304.4	c.4472A>G	p.K1491R	0.500
VRL4	ALK	NM_004304.4	c.4381A>G	p.I1461V	1.000
VRL4	EPCAM	NM_002354.2	c.344T>C	p.M115T	1.000
VRL4	MSH6	NM_000179.2	c.116G>A	p.G39E	0.500
VRL4	LRP1B	NM_018557.2	c.143A>G	p.Q48R	1.000
VRL4	CTLA4	NM_005214.4	c.49A>G	p.T17A	0.500
VRL4	BARD1	NM_000465.3	c.1134G>C	p.R378S	0.500
VRL4	BARD1	NM_000465.3	c.70C>T	p.P24S	0.500
VRL4	CUL3	NM_001257198.1	c.1717G>A	p.V573I	1.000
VRL4	PDCD1	NM_005018.2	c.644C>T	p.A215V	1.000
VRL4	ASXL1	NM_015338.5	c.1954G>A	p.G652S	0.500
VRL4	ASXL1	NM_015338.5	c.2444T>C	p.L815P	1.000
VRL4	PTPRT	NM_133170.3	c.85G>C	p.A29P	0.500
VRL4	NCOA3	NM_181659.2	c.3789_3791del	p.Q1276del	0.500
VRL4	ZNF217	NM_006526.2	c.1643C>T	p.T548I	0.500
VRL4	AURKA	NM_003600.3	c.169A>G	p.I57V	0.500
VRL4	AURKA	NM_003600.3	c.91T>A	p.F31I	0.500
VRL4	RTEL1	NM_001283009.1	c.3126A>C	p.Q1042H	0.500
VRL4	ICOSLG	NM_001283050.1	c.382G>A	p.V128I	0.500
VRL4	EP300	NM_001429.3	c.2989A>G	p.I997V	0.500
VRL4	SETD2	NM_014159.6	c.5885C>T	p.P1962L	1.000
VRL4	ATR	NM_001184.3	c.632T>C	p.M211T	0.500
VRL4	KDR	NM_002253.2	c.889G>A	p.V297I	0.500
VRL4	FAT1	NM_005245.3	c.12177G>C	p.K4059N	1.000
VRL4	FAT1	NM_005245.3	c.10660T>G	p.S3554A	1.000
VRL4	FAT1	NM_005245.3	c.10001T>C	p.V3334A	0.500
VRL4	FAT1	NM_005245.3	c.8798A>C	p.Q2933P	1.000
VRL4	FAT1	NM_005245.3	c.7105A>C	p.T2369P	0.500
VRL4	FAT1	NM_005245.3	c.6822C>G	p.D2274E	0.500
VRL4	FAT1	NM_005245.3	c.4985A>G	p.N1662S	0.500
VRL4	FAT1	NM_005245.3	c.4690G>A	p.A1564T	0.500
VRL4	FAT1	NM_005245.3	c.3818A>G	p.H1273R	1.000
VRL4	DROSHA	NM_013235.4	c.785A>G	p.D262G	0.500
VRL4	IL7R	NM_002185.3	c.197T>C	p.I66T	0.500
VRL4	IL7R	NM_002185.3	c.412G>A	p.V138I	0.500
VRL4	RICTOR	NM_001285439.1	c.2510C>T	p.S837F	0.500
VRL4	MAP3K1	NM_005921.1	c.2416G>A	p.D806N	0.500
VRL4	MAP3K1	NM_005921.1	c.2716G>A	p.V906I	1.000
VRL4	MAP3K1	NM_005921.1	c.2845_2847del	p.T949del	1.000
VRL4	PIK3R1	NM_181523.2	c.978G>A	p.M326I	0.500
VRL4	MSH3	NM_002439.4	c.235A>G	p.I79V	1.000
VRL4	MSH3	NM_002439.4	c.2846A>G	p.Q949R	1.000
VRL4	MSH3	NM_002439.4	c.3133G>A	p.A1045T	0.500
VRL4	APC	NM_000038.5	c.5465T>A	p.V1822D	0.500
VRL4	FGFR4	NM_002011.4	c.407C>T	p.P136L	1.000
VRL4	FGFR4	NM_002011.4	c.1162G>A	p.G388R	0.500
VRL4	NSD1	NM_022455.4	c.1840G>T	p.V614L	0.500
VRL4	NSD1	NM_022455.4	c.2176T>C	p.S726P	0.500
VRL4	FLT4	NM_182925.4	c.2670C>G	p.H890Q	0.500
VRL4	NOTCH4	NM_004557.3	c.958A>G	p.T320A	1.000
VRL4	NOTCH4	NM_004557.3	c.349A>C	p.K117Q	1.000
VRL4	NOTCH4	NM_004557.3	c.45_47dup	p.L16dup	0.500
VRL4	TAP1	NM_000593.5	c.2090A>G	p.D697G	0.500
VRL4	TAP1	NM_000593.5	c.1177A>G	p.I393V	0.500

VRL4	CDKN1A	NM_001291549.1	c.83A>G	p.D28G	0.500
VRL4	CDKN1A	NM_001291549.1	c.195C>A	p.S65R	1.000
VRL4	PIM1	NM_002648.3	c.403G>A	p.E135K	0.500
VRL4	CCND3	NM_001760.4	c.775T>G	p.S259A	1.000
VRL4	ROS1	NM_002944.2	c.1611A>G	p.I537M	1.000
VRL4	ROS1	NM_002944.2	c.433A>C	p.T145P	1.000
VRL4	PMS2	NM_000535.6	c.1621A>G	p.K541E	1.000
VRL4	PMS2	NM_000535.6	c.1454C>A	p.T485K	0.500
VRL4	EGFR	NM_005228.3	c.1562G>A	p.R521K	0.500
VRL4	KEL	NM_000420.2	c.1224G>A	p.M408I	0.500
VRL4	KMT2C	NM_170606.2	c.2963G>T	p.C988F	0.500
VRL4	KMT2C	NM_170606.2	c.2512G>A	p.G838S	0.500
VRL4	KMT2C	NM_170606.2	c.2447dup	p.Y816*	0.500
VRL4	PREX2	NM_024870.3	c.4463C>T	p.S1488L	0.500
VRL4	NBN	NM_002485.4	c.553G>C	p.E185Q	0.500
VRL4	RECQL4	NM_004260.3	c.274T>C	p.S92P	1.000
VRL4	PDCD1LG2	NM_025239.3	c.686T>C	p.F229S	1.000
VRL4	PTPRD	NM_002839.3	c.1339C>G	p.Q447E	1.000
VRL4	MTAP	NM_002451.3	c.166G>A	p.V56I	0.500
VRL4	PTCH1	NM_000264.3	c.3944C>T	p.P1315L	0.500
VRL4	KDM6A	NM_001291415.1	c.2333C>A	p.T778K	0.500
VRL4	AR	NM_000044.3	c.219_239del	p.Q74_Q80del	0.500
VRL4	ATRX	NM_000489.4	c.2785C>G	p.Q929E	1.000
VRL4	BCORL1	NM_001184772.2	c.331T>C	p.F111L	1.000
VRL4	CCNQ	NM_152274.4	c.16dup	p.G7Rfs*51	0.500
VRL5	MCL1	NM_021960.4	c.824C>T	p.A275V	0.025
VRL5	MCL1	NM_021960.4	c.670C>T	p.H224Y	0.010
VRL5	BTG2	NM_006763.2	c.185G>A	p.G62D	0.032
VRL5	ETV6	NM_001987.4	c.26G>A	p.S9N	0.494
VRL5	CDKN1B	NM_004064.4	c.448G>A	p.G150R	0.398
VRL5	ARID2	NM_152641.2	c.2936C>G	p.A979G	0.505
VRL5	KMT2D	NM_003482.3	c.9505G>A	p.G3169S	0.027
VRL5	BTG1	NM_001731.2	c.250G>A	p.A84T	0.470
VRL5	BTG1	NM_001731.2	c.138G>C	p.E46D	0.496
VRL5	BTG1	NM_001731.2	c.80G>A	p.R27H	0.478
VRL5	CREBBP	NM_004380.2	c.631G>A	p.G211R	0.490
VRL5	ANKRD11	NM_013275.5	c.1786C>T	p.Q596*	0.491
VRL5	STAT3	NM_139276.2	c.1636T>C	p.W546R	0.503
VRL5	MYD88	NM_002468.4	c.794T>C	p.L265P	0.503
VRL5	IRF4	NM_002460.3	c.196G>A	p.E66K	0.922
VRL5	IRF4	NM_002460.3	c.208C>G	p.L70V	0.925
VRL5	MYC	NM_002467.4	c.100G>C	p.V34L	0.479
VRL5	MYC	NM_002467.4	c.632C>G	p.P211R	0.482
VRL5	ID3	NM_002167.4	c.313A>G	p.T105A	1.000
VRL5	MYCL	NM_001033082.2	c.1175C>G	p.T392S	1.000
VRL5	MUTYH	NM_001128425.1	c.1014G>C	p.Q338H	0.500
VRL5	BCL10	NM_003921.4	c.638G>A	p.G213E	0.500
VRL5	FAM46C	NM_017709.3	c.201C>G	p.H67Q	0.500
VRL5	HSD3B1	NM_000862.2	c.1100C>A	p.T367N	1.000
VRL5	NOTCH2	NM_024408.3	c.57C>G	p.C19W	0.500
VRL5	TET1	NM_030625.2	c.485A>G	p.D162G	0.500
VRL5	TET1	NM_030625.2	c.3369A>G	p.I1123M	1.000
VRL5	MEN1	NM_000244.3	c.1636A>G	p.T546A	1.000
VRL5	RPS6KB2	NM_003952.2	c.1259C>T	p.A420V	1.000
VRL5	ATM	NM_000051.3	c.5948A>G	p.N1983S	1.000
VRL5	KDM5A	NM_001042603.2	c.2594T>C	p.M865T	1.000

VRL5	H3F3C	NM_001013699.2	c.116A>C	p.H39P	0.500
VRL5	SH2B3	NM_005475.2	c.724C>T	p.P242S	0.500
VRL5	SH2B3	NM_005475.2	c.784T>C	p.W262R	1.000
VRL5	HNF1A	NM_000545.6	c.1720A>G	p.S574G	1.000
VRL5	LATS2	NM_014572.2	c.1361C>T	p.P454L	0.500
VRL5	LATS2	NM_014572.2	c.1087G>A	p.G363S	1.000
VRL5	LATS2	NM_014572.2	c.971C>T	p.A324V	0.500
VRL5	FLT3	NM_004119.2	c.680C>T	p.T227M	0.500
VRL5	BRCA2	NM_000059.3	c.7397T>C	p.V2466A	1.000
VRL5	DIS3	NM_014953.4	c.806A>G	p.N269S	1.000
VRL5	IRS2	NM_003749.2	c.3170G>A	p.G1057D	0.500
VRL5	CUL4A	NM_001008895.2	c.1931A>G	p.K644R	1.000
VRL5	BCL2L2	NM_004050.4	c.398A>G	p.Q133R	1.000
VRL5	PRKD1	NM_001330069.1	c.91_96dup	p.S31_G32dup	0.500
VRL5	FOXA1	NM_004496.3	c.247G>A	p.A83T	1.000
VRL5	MLH3	NM_001040108.1	c.2476A>G	p.N826D	1.000
VRL5	TSHR	NM_000369.2	c.2181G>C	p.E727D	1.000
VRL5	LTK	NM_002344.5	c.125G>A	p.R42Q	0.500
VRL5	PALB2	NM_024675.3	c.1676A>G	p.Q559R	0.500
VRL5	ZFHX3	NM_006885.3	c.10567_10581del	p.G3523_G3527del	0.500
VRL5	ZFHX3	NM_006885.3	c.10554_10556del	p.G3527del	0.500
VRL5	ZFHX3	NM_006885.3	c.10527_10532del	p.G3511_G3512del	0.500
VRL5	ZFHX3	NM_006885.3	c.5218_5223del	p.Q1740_Q1741del	0.500
VRL5	ANKRD11	NM_013275.5	c.2912C>T	p.A971V	0.500
VRL5	FANCA	NM_000135.2	c.2426G>A	p.G809D	1.000
VRL5	FANCA	NM_000135.2	c.1927C>G	p.P643A	0.500
VRL5	FANCA	NM_000135.2	c.1501G>A	p.G501S	0.500
VRL5	FANCA	NM_000135.2	c.1235C>T	p.A412V	0.500
VRL5	FANCA	NM_000135.2	c.796A>G	p.T266A	1.000
VRL5	AURKB	NM_001284526.1	c.896T>C	p.M299T	0.500
VRL5	ERBB2	NM_004448.3	c.3508C>G	p.P1170A	0.500
VRL5	BRCA1	NM_007294.3	c.4837A>G	p.S1613G	0.500
VRL5	BRCA1	NM_007294.3	c.3548A>G	p.K1183R	0.500
VRL5	BRCA1	NM_007294.3	c.3113A>G	p.E1038G	0.500
VRL5	BRCA1	NM_007294.3	c.2612C>T	p.P871L	0.500
VRL5	RNF43	NM_017763.5	c.350G>A	p.R117H	0.500
VRL5	BRIP1	NM_032043.2	c.2755T>C	p.S919P	0.500
VRL5	AXIN2	NM_004655.3	c.148C>T	p.P50S	1.000
VRL5	TCF3	NM_001136139.2	c.1475C>T	p.A492V	0.500
VRL5	DOT1L	NM_032482.2	c.4156G>A	p.G1386S	0.500
VRL5	PTPRS	NM_002850.3	c.4369T>C	p.C1457R	1.000
VRL5	NOTCH3	NM_000435.2	c.6668C>T	p.A2223V	1.000
VRL5	PIK3R2	NM_005027.3	c.700A>C	p.S234R	1.000
VRL5	PIK3R2	NM_005027.3	c.937T>C	p.S313P	1.000
VRL5	CEBPA	NM_004364.4	c.584_589dup	p.H195_P196dup	0.500
VRL5	KMT2B	NM_014727.2	c.3059dup	p.R1021Pfs*14	1.000
VRL5	KMT2B	NM_014727.2	c.5486C>T	p.P1829L	0.500
VRL5	KMT2B	NM_014727.2	c.7091A>G	p.D2364G	0.500
VRL5	AXL	NM_021913.4	c.796A>G	p.N266D	1.000
VRL5	CD3EAP	NM_001297590.1	c.1516C>A	p.Q506K	0.500
VRL5	ALK	NM_004304.4	c.4587C>G	p.D1529E	1.000
VRL5	ALK	NM_004304.4	c.4472A>G	p.K1491R	1.000
VRL5	ALK	NM_004304.4	c.4381A>G	p.I1461V	1.000
VRL5	EPCAM	NM_002354.2	c.344T>C	p.M115T	1.000
VRL5	MERTK	NM_006343.2	c.1397G>A	p.R466K	0.500
VRL5	MERTK	NM_006343.2	c.1552A>G	p.I518V	0.500

VRL5	LRP1B	NM_018557.2	c.143A>G	p.Q48R	0.500
VRL5	CTLA4	NM_005214.4	c.49A>G	p.T17A	0.500
VRL5	ASXL1	NM_015338.5	c.2444T>C	p.L815P	1.000
VRL5	DNMT3B	NM_006892.3	c.1610G>A	p.R537Q	0.500
VRL5	PTPRT	NM_133170.3	c.85G>C	p.A29P	1.000
VRL5	NCOA3	NM_181659.2	c.3789_3791del	p.Q1276del	0.500
VRL5	AURKA	NM_003600.3	c.169A>G	p.I57V	1.000
VRL5	AURKA	NM_003600.3	c.91T>A	p.F31I	1.000
VRL5	TMPRSS2	NM_005656.3	c.478G>A	p.V160M	0.500
VRL5	SETD2	NM_014159.6	c.5885C>T	p.P1962L	1.000
VRL5	SETD2	NM_014159.6	c.3358A>G	p.I1120V	0.500
VRL5	PPP4R2	NM_174907.3	c.906_917del	p.D304_E307del	0.500
VRL5	GATA2	NM_032638.4	c.490G>A	p.A164T	1.000
VRL5	ATR	NM_001184.3	c.632T>C	p.M211T	0.500
VRL5	BCL6	NM_001706.4	c.1477G>A	p.A493T	0.500
VRL5	PHOX2B	NM_003924.3	c.765_779del	p.A256_A260del	0.500
VRL5	KIT	NM_000222.2	c.1621A>C	p.M541L	0.500
VRL5	ABRAXAS1	NM_139076.2	c.1042G>A	p.A348T	1.000
VRL5	FAT1	NM_005245.3	c.12177G>C	p.K4059N	1.000
VRL5	FAT1	NM_005245.3	c.10660T>G	p.S3554A	0.500
VRL5	FAT1	NM_005245.3	c.8798A>C	p.Q2933P	0.500
VRL5	FAT1	NM_005245.3	c.7105A>C	p.T2369P	0.500
VRL5	FAT1	NM_005245.3	c.6822C>G	p.D2274E	0.500
VRL5	FAT1	NM_005245.3	c.4690G>A	p.A1564T	0.500
VRL5	FAT1	NM_005245.3	c.3818A>G	p.H1273R	0.500
VRL5	FAT1	NM_005245.3	c.3190A>G	p.R1064G	0.500
VRL5	FAT1	NM_005245.3	c.2584G>C	p.V862L	0.500
VRL5	FAT1	NM_005245.3	c.1842C>G	p.F614L	0.500
VRL5	FAT1	NM_005245.3	c.392C>T	p.A131V	0.500
VRL5	DROSHA	NM_013235.4	c.962C>T	p.S321L	0.500
VRL5	RICTOR	NM_001285439.1	c.2510C>T	p.S837F	0.500
VRL5	MAP3K1	NM_005921.1	c.2416G>A	p.D806N	1.000
VRL5	MAP3K1	NM_005921.1	c.2716G>A	p.V906I	1.000
VRL5	MAP3K1	NM_005921.1	c.2845_2847del	p.T949del	0.500
VRL5	PIK3R1	NM_181523.2	c.978G>A	p.M326I	0.500
VRL5	MSH3	NM_002439.4	c.181_189dup	p.A61_P63dup	0.500
VRL5	MSH3	NM_002439.4	c.235A>G	p.I79V	0.500
VRL5	MSH3	NM_002439.4	c.2846A>G	p.Q949R	1.000
VRL5	MSH3	NM_002439.4	c.3133G>A	p.A1045T	0.500
VRL5	APC	NM_000038.5	c.5465T>A	p.V1822D	1.000
VRL5	FGFR4	NM_002011.4	c.28G>A	p.V10I	0.500
VRL5	FGFR4	NM_002011.4	c.407C>T	p.P136L	1.000
VRL5	FGFR4	NM_002011.4	c.1162G>A	p.G388R	0.500
VRL5	MDC1	NM_014641.2	c.5372T>A	p.V1791E	0.500
VRL5	MDC1	NM_014641.2	c.5234C>G	p.P1745R	0.500
VRL5	MDC1	NM_014641.2	c.3896G>A	p.R1299Q	0.500
VRL5	MDC1	NM_014641.2	c.3847C>A	p.P1283T	0.500
VRL5	MDC1	NM_014641.2	c.3797A>C	p.Y1266S	0.500
VRL5	MDC1	NM_014641.2	c.3620T>C	p.L1207P	0.500
VRL5	MDC1	NM_014641.2	c.3601A>C	p.T1201P	0.500
VRL5	MDC1	NM_014641.2	c.3335C>T	p.S1112F	0.500
VRL5	MDC1	NM_014641.2	c.3298C>G	p.P1100A	0.500
VRL5	NOTCH4	NM_004557.3	c.2824G>A	p.G942R	1.000
VRL5	NOTCH4	NM_004557.3	c.682G>A	p.G228S	1.000
VRL5	NOTCH4	NM_004557.3	c.36_47del	p.L13_L16del	1.000
VRL5	TAP1	NM_000593.5	c.1177A>G	p.I393V	1.000

VRL5	CDKN1A	NM_001291549.1	c.195C>A	p.S65R	0.500
VRL5	CCND3	NM_001760.4	c.775T>G	p.S259A	0.500
VRL5	ROS1	NM_002944.2	c.6686C>G	p.S2229C	0.500
VRL5	ROS1	NM_002944.2	c.6682A>C	p.K2228Q	0.500
VRL5	ROS1	NM_002944.2	c.6637G>A	p.D2213N	0.500
VRL5	ROS1	NM_002944.2	c.500G>A	p.R167Q	0.500
VRL5	PMS2	NM_000535.6	c.1621A>G	p.K541E	1.000
VRL5	PMS2	NM_000535.6	c.1408C>T	p.P470S	0.500
VRL5	ETV1	NM_004956.4	c.298A>G	p.S100G	0.500
VRL5	EGFR	NM_005228.3	c.1562G>A	p.R521K	1.000
VRL5	MET	NM_000245.3	c.1124A>G	p.N375S	0.500
VRL5	KMT2C	NM_170606.2	c.10979C>T	p.S3660L	0.500
VRL5	KMT2C	NM_170606.2	c.10639T>C	p.S3547P	0.500
VRL5	KMT2C	NM_170606.2	c.2959T>C	p.Y987H	0.500
VRL5	KMT2C	NM_170606.2	c.2512G>A	p.G838S	0.500
VRL5	NBN	NM_002485.4	c.553G>C	p.E185Q	1.000
VRL5	RECQL4	NM_004260.3	c.3014G>A	p.R1005Q	1.000
VRL5	RECQL4	NM_004260.3	c.801G>C	p.E267D	1.000
VRL5	RECQL4	NM_004260.3	c.274T>C	p.S92P	1.000
VRL5	PDCD1LG2	NM_025239.3	c.686T>C	p.F229S	1.000
VRL5	PTCH1	NM_000264.3	c.3944C>T	p.P1315L	1.000
VRL5	EGFL7	NM_016215.4	c.457G>A	p.V153I	0.500
VRL5	ATRX	NM_000489.4	c.2785C>G	p.Q929E	0.500
VRL5	BCORL1	NM_001184772.2	c.331T>C	p.F111L	1.000
VRL5	CCNQ	NM_152274.4	c.16dup	p.G7Rfs*51	1.000
VRL6	BTG2	NM_006763.2	c.82G>A	p.G28S	0.319
VRL6	BTG2	NM_006763.2	c.136C>G	p.L46V	0.309
VRL6	CXCR4	NM_003467.2	c.164G>A	p.G55D	0.486
VRL6	MYD88	NM_002468.4	c.794T>C	p.L265P	0.489
VRL6	HIST1H1C	NM_005319.3	c.178G>A	p.A60T	0.486
VRL6	HIST1H3I	NM_003533.2	c.217C>T	p.R73W	0.013
VRL6	HIST1H3I	NM_003533.2	c.180G>C	p.E60D	0.482
VRL6	PIM1	NM_002648.3	c.83G>A	p.G28D	0.490
VRL6	PIM1	NM_002648.3	c.183C>G	p.N61K	0.475
VRL6	PIM1	NM_002648.3	c.302C>G	p.S101C	0.494
VRL6	PIM1	NM_002648.3	c.373C>G	p.P125A	0.468
VRL6	PIM1	NM_002648.3	c.427C>A	p.L143M	0.470
VRL6	PIM1	NM_002648.3	c.529C>A	p.L177I	0.117
VRL6	PIM1	NM_002648.3	c.544C>T	p.L182F	0.482
VRL6	PIM1	NM_002648.3	c.572C>T	p.A191V	0.486
VRL6	PIM1	NM_002648.3	c.587C>A	p.T196N	0.473
VRL6	PREX2	NM_024870.3	c.2246C>T	p.T749M	0.492
VRL6	MYC	NM_002467.4	c.573G>C	p.L191F	0.495
VRL6	ID3	NM_002167.4	c.313A>G	p.T105A	1.000
VRL6	CSF3R	NM_156039.3	c.2278C>A	p.P760T	1.000
VRL6	MYCL	NM_001033082.2	c.1175C>G	p.T392S	1.000
VRL6	HSD3B1	NM_000862.2	c.1100C>A	p.T367N	1.000
VRL6	NOTCH2	NM_024408.3	c.57C>G	p.C19W	0.500
VRL6	NTRK1	NM_002529.3	c.1114G>T	p.A372S	0.500
VRL6	TET1	NM_030625.2	c.485A>G	p.D162G	0.500
VRL6	TET1	NM_030625.2	c.3369A>G	p.I1123M	0.500
VRL6	BMPR1A	NM_004329.2	c.4C>A	p.P2T	1.000
VRL6	MEN1	NM_000244.3	c.1636A>G	p.T546A	0.500
VRL6	RPS6KB2	NM_003952.2	c.1259C>T	p.A420V	0.500
VRL6	INPPL1	NM_001567.3	c.3248C>G	p.A1083G	0.500
VRL6	ATM	NM_000051.3	c.5948A>G	p.N1983S	1.000

VRL6	KDM5A	NM_001042603.2	c.2594T>C	p.M865T	1.000
VRL6	CDKN1B	NM_004064.4	c.326T>G	p.V109G	0.500
VRL6	ARID2	NM_152641.2	c.4300G>T	p.A1434S	0.500
VRL6	GLI1	NM_005269.2	c.2798G>A	p.G933D	0.500
VRL6	GLI1	NM_005269.2	c.3298G>C	p.E1100Q	0.500
VRL6	SH2B3	NM_005475.2	c.784T>C	p.W262R	1.000
VRL6	HNF1A	NM_000545.6	c.79A>C	p.I27L	1.000
VRL6	HNF1A	NM_000545.6	c.1460G>A	p.S487N	1.000
VRL6	HNF1A	NM_000545.6	c.1720A>G	p.S574G	1.000
VRL6	LATS2	NM_014572.2	c.1087G>A	p.G363S	1.000
VRL6	FLT3	NM_004119.2	c.680C>T	p.T227M	1.000
VRL6	FLT3	NM_004119.2	c.20A>G	p.D7G	0.500
VRL6	BRCA2	NM_000059.3	c.1114A>C	p.N372H	1.000
VRL6	BRCA2	NM_000059.3	c.7397T>C	p.V2466A	1.000
VRL6	DIS3	NM_014953.4	c.977C>G	p.T326R	1.000
VRL6	IRS2	NM_003749.2	c.3170G>A	p.G1057D	1.000
VRL6	CUL4A	NM_001008895.2	c.1931A>G	p.K644R	0.500
VRL6	BCL2L2	NM_004050.4	c.398A>G	p.Q133R	1.000
VRL6	PRKD1	NM_001330069.1	c.91_96dup	p.S31_G32dup	0.500
VRL6	FOXA1	NM_004496.3	c.247G>A	p.A83T	0.500
VRL6	MLH3	NM_001040108.1	c.2476A>G	p.N826D	1.000
VRL6	TSHR	NM_000369.2	c.2181G>C	p.E727D	1.000
VRL6	LTK	NM_002344.5	c.125G>A	p.R42Q	0.500
VRL6	MGA	NM_001164273.1	c.4567C>G	p.P1523A	0.500
VRL6	PALB2	NM_024675.3	c.1676A>G	p.Q559R	0.500
VRL6	ZFHX3	NM_006885.3	c.10554_10556del	p.G3527del	0.500
VRL6	ZFHX3	NM_006885.3	c.214T>G	p.S72A	1.000
VRL6	ANKRD11	NM_013275.5	c.2912C>T	p.A971V	1.000
VRL6	FANCA	NM_000135.2	c.3982A>G	p.T1328A	0.500
VRL6	FANCA	NM_000135.2	c.2426G>A	p.G809D	1.000
VRL6	FANCA	NM_000135.2	c.1927C>G	p.P643A	0.500
VRL6	FANCA	NM_000135.2	c.1501G>A	p.G501S	1.000
VRL6	FANCA	NM_000135.2	c.1235C>T	p.A412V	0.500
VRL6	FANCA	NM_000135.2	c.796A>G	p.T266A	1.000
VRL6	TP53	NM_000546.5	c.215C>G	p.P72R	1.000
VRL6	ERBB2	NM_004448.3	c.3508C>G	p.P1170A	0.500
VRL6	BRCA1	NM_007294.3	c.4837A>G	p.S1613G	0.500
VRL6	BRCA1	NM_007294.3	c.3548A>G	p.K1183R	0.500
VRL6	BRCA1	NM_007294.3	c.3113A>G	p.E1038G	0.500
VRL6	BRCA1	NM_007294.3	c.2612C>T	p.P871L	0.500
VRL6	RNF43	NM_017763.5	c.1252C>A	p.L418M	0.500
VRL6	RNF43	NM_017763.5	c.350G>A	p.R117H	0.500
VRL6	RNF43	NM_017763.5	c.139A>G	p.I47V	1.000
VRL6	BRIP1	NM_032043.2	c.2755T>C	p.S919P	1.000
VRL6	AXIN2	NM_004655.3	c.1250C>T	p.A417V	0.500
VRL6	AXIN2	NM_004655.3	c.148C>T	p.P50S	0.500
VRL6	BCL2	NM_000633.2	c.127G>A	p.A43T	0.500
VRL6	TCF3	NM_001136139.2	c.1475C>T	p.A492V	0.500
VRL6	PTPRS	NM_002850.3	c.4369T>C	p.C1457R	1.000
VRL6	NOTCH3	NM_000435.2	c.6668C>T	p.A2223V	0.500
VRL6	NOTCH3	NM_000435.2	c.6100C>G	p.P2034A	0.500
VRL6	PIK3R2	NM_005027.3	c.700A>C	p.S234R	1.000
VRL6	PIK3R2	NM_005027.3	c.937T>C	p.S313P	1.000
VRL6	KMT2B	NM_014727.2	c.3059dup	p.R1021Pfs*14	1.000
VRL6	KMT2B	NM_014727.2	c.7091A>G	p.D2364G	0.500
VRL6	AXL	NM_021913.4	c.796A>G	p.N266D	1.000

VRL6	CD3EAP	NM_001297590.1	c.1516C>A	p.Q506K	0.500
VRL6	ALK	NM_004304.4	c.4587C>G	p.D1529E	1.000
VRL6	ALK	NM_004304.4	c.4472A>G	p.K1491R	1.000
VRL6	ALK	NM_004304.4	c.4381A>G	p.I1461V	1.000
VRL6	EPAS1	NM_001430.4	c.2296A>C	p.T766P	0.500
VRL6	EPCAM	NM_002354.2	c.344T>C	p.M115T	1.000
VRL6	MSH6	NM_000179.2	c.116G>A	p.G39E	0.500
VRL6	LRP1B	NM_018557.2	c.143A>G	p.Q48R	0.500
VRL6	CTLA4	NM_005214.4	c.49A>G	p.T17A	1.000
VRL6	BARD1	NM_000465.3	c.1134G>C	p.R378S	0.500
VRL6	BARD1	NM_000465.3	c.70C>T	p.P24S	0.500
VRL6	PDCD1	NM_005018.2	c.644C>T	p.A215V	1.000
VRL6	ASXL1	NM_015338.5	c.2444T>C	p.L815P	1.000
VRL6	PTPRT	NM_133170.3	c.85G>C	p.A29P	1.000
VRL6	NCOA3	NM_181659.2	c.3753_3761del	p.Q1274_Q1276del	0.500
VRL6	ZNF217	NM_006526.2	c.1643C>T	p.T548I	0.500
VRL6	AURKA	NM_003600.3	c.169A>G	p.I57V	1.000
VRL6	AURKA	NM_003600.3	c.91T>A	p.F31I	1.000
VRL6	SETD2	NM_014159.6	c.3240G>A	p.M1080I	0.500
VRL6	GATA2	NM_032638.4	c.490G>A	p.A164T	0.500
VRL6	ATR	NM_001184.3	c.632T>C	p.M211T	0.500
VRL6	KDR	NM_002253.2	c.1416A>T	p.Q472H	1.000
VRL6	KDR	NM_002253.2	c.889G>A	p.V297I	0.500
VRL6	FAT1	NM_005245.3	c.12177G>C	p.K4059N	1.000
VRL6	FAT1	NM_005245.3	c.11818A>G	p.T3940A	0.500
VRL6	FAT1	NM_005245.3	c.10001T>C	p.V3334A	0.500
VRL6	FAT1	NM_005245.3	c.8798A>C	p.Q2933P	0.500
VRL6	FAT1	NM_005245.3	c.8417G>A	p.S2806N	0.500
VRL6	FAT1	NM_005245.3	c.4985A>G	p.N1662S	0.500
VRL6	FAT1	NM_005245.3	c.4475C>G	p.T1492S	0.500
VRL6	FAT1	NM_005245.3	c.3818A>G	p.H1273R	0.500
VRL6	FAT1	NM_005245.3	c.3190A>G	p.R1064G	0.500
VRL6	FAT1	NM_005245.3	c.2584G>C	p.V862L	0.500
VRL6	FAT1	NM_005245.3	c.1842C>G	p.F614L	0.500
VRL6	FAT1	NM_005245.3	c.1444G>A	p.V482I	0.500
VRL6	FAT1	NM_005245.3	c.1212T>G	p.S404R	0.500
VRL6	SDHA	NM_004168.3	c.550G>A	p.G184R	0.500
VRL6	IL7R	NM_002185.3	c.197T>C	p.I66T	1.000
VRL6	IL7R	NM_002185.3	c.412G>A	p.V138I	1.000
VRL6	IL7R	NM_002185.3	c.731C>T	p.T244I	1.000
VRL6	RICTOR	NM_001285439.1	c.2510C>T	p.S837F	0.500
VRL6	MAP3K1	NM_005921.1	c.2416G>A	p.D806N	1.000
VRL6	MAP3K1	NM_005921.1	c.2716G>A	p.V906I	1.000
VRL6	MAP3K1	NM_005921.1	c.2845_2847del	p.T949del	0.500
VRL6	PIK3R1	NM_181523.2	c.978G>A	p.M326I	0.500
VRL6	MSH3	NM_002439.4	c.181_189dup	p.A61_P63dup	0.500
VRL6	MSH3	NM_002439.4	c.235A>G	p.I79V	0.500
VRL6	MSH3	NM_002439.4	c.2846A>G	p.Q949R	1.000
VRL6	MSH3	NM_002439.4	c.3133G>A	p.A1045T	1.000
VRL6	APC	NM_000038.5	c.5465T>A	p.V1822D	1.000
VRL6	CSF1R	NM_005211.3	c.1085A>G	p.H362R	0.500
VRL6	CSF1R	NM_005211.3	c.835G>A	p.V279M	0.500
VRL6	FGFR4	NM_002011.4	c.28G>A	p.V10I	1.000
VRL6	FGFR4	NM_002011.4	c.407C>T	p.P136L	1.000
VRL6	FGFR4	NM_002011.4	c.535A>G	p.T179A	0.500
VRL6	NSD1	NM_022455.4	c.1840G>T	p.V614L	0.500

VRL6	NSD1	NM_022455.4	c.2176T>C	p.S726P	0.500
VRL6	HIST1H1C	NM_005319.3	c.53C>T	p.A18V	0.500
VRL6	NOTCH4	NM_004557.3	c.349A>C	p.K117Q	0.500
VRL6	NOTCH4	NM_004557.3	c.45_47del	p.L16del	0.500
VRL6	TAP2	NM_000544.3	c.1135G>A	p.V379I	0.500
VRL6	TAP1	NM_000593.5	c.1177A>G	p.I393V	0.500
VRL6	CDKN1A	NM_001291549.1	c.83A>G	p.D28G	0.500
VRL6	CDKN1A	NM_001291549.1	c.195C>A	p.S65R	0.500
VRL6	CCND3	NM_001760.4	c.797C>T	p.A266V	0.500
VRL6	CCND3	NM_001760.4	c.775T>G	p.S259A	0.500
VRL6	PMS2	NM_000535.6	c.1621A>G	p.K541E	1.000
VRL6	PMS2	NM_000535.6	c.1454C>A	p.T485K	1.000
VRL6	ETV1	NM_004956.4	c.298A>G	p.S100G	1.000
VRL6	EGFR	NM_005228.3	c.1562G>A	p.R521K	0.500
VRL6	EPHB4	NM_004444.4	c.1112C>T	p.A371V	0.500
VRL6	EZH2	NM_004456.4	c.553G>C	p.D185H	0.500
VRL6	KMT2C	NM_170606.2	c.10979C>T	p.S3660L	0.500
VRL6	KMT2C	NM_170606.2	c.2959T>C	p.Y987H	0.500
VRL6	KMT2C	NM_170606.2	c.2512G>A	p.G838S	0.500
VRL6	PREX2	NM_024870.3	c.4180C>T	p.R1394W	0.500
VRL6	NBN	NM_002485.4	c.553G>C	p.E185Q	0.500
VRL6	RECQL4	NM_004260.3	c.3014G>A	p.R1005Q	0.500
VRL6	RECQL4	NM_004260.3	c.274T>C	p.S92P	1.000
VRL6	PDCD1LG2	NM_025239.3	c.686T>C	p.F229S	1.000
VRL6	PTPRD	NM_002839.3	c.2983C>T	p.R995C	0.500
VRL6	PTPRD	NM_002839.3	c.2585G>T	p.R862L	0.500
VRL6	PTPRD	NM_002839.3	c.1339C>G	p.Q447E	0.500
VRL6	MTAP	NM_002451.3	c.166G>A	p.V56I	0.500
VRL6	PTCH1	NM_000264.3	c.4324C>T	p.R1442W	0.500
VRL6	PTCH1	NM_000264.3	c.3944C>T	p.P1315L	1.000
VRL6	AR	NM_000044.3	c.228_239dup	p.Q77_Q80dup	0.500
VRL6	BCORL1	NM_001184772.2	c.331T>C	p.F111L	1.000
VRL6	CCNQ	NM_152274.4	c.16dup	p.G7Rfs*51	0.500
VRL7	MTOR	NM_004958.3	c.1712C>T	p.T571M	0.408
VRL7	CDC73	NM_024529.4	c.515C>T	p.S172F	0.476
VRL7	BTG2	NM_006763.2	c.83G>A	p.G28D	0.208
VRL7	BTG2	NM_006763.2	c.142G>C	p.E48Q	0.365
VRL7	KMT2D	NM_003482.3	c.13938_13944dup	p.G4649Rfs*15	0.442
VRL7	HNF1A	NM_000545.6	c.499G>A	p.V167I	0.471
VRL7	GNA13	NM_006572.5	c.943_945del	p.D315del	0.453
VRL7	BRD4	NM_058243.2	c.1991dup	p.R665Afs*2	0.414
VRL7	MEF2B	NM_001145785.1	c.97T>C	p.Y33H	0.476
VRL7	CD79A	NM_001783.3	c.573_617del	p.N192_L206del	0.150
VRL7	LRP1B	NM_018557.2	c.1036G>A	p.G346R	0.010
VRL7	ZNF217	NM_006526.2	c.2065T>G	p.L689V	0.463
VRL7	MYD88	NM_002468.4	c.794T>C	p.L265P	0.493
VRL7	MITF	NM_000248.3	c.328C>T	p.R110*	0.458
VRL7	WWTR1	NM_015472.4	c.688C>T	p.Q230*	0.460
VRL7	CCND3	NM_001760.4	c.869T>A	p.I290K	0.553
VRL7	EPHA7	NM_004440.3	c.2834C>T	p.T945M	0.407
VRL7	KMT2C	NM_170606.2	c.7208G>A	p.R2403Q	0.950
VRL7	SOX17	NM_022454.3	c.424C>T	p.R142C	0.488
VRL7	NTRK2	NM_006180.4	c.1659C>A	p.N553K	0.505
VRL7	P2RY8	NM_178129.4	c.373G>A	p.V125I	0.027
VRL7	P2RY8	NM_178129.4	c.30C>G	p.D10E	0.926
VRL7	ID3	NM_002167.4	c.313A>G	p.T105A	1.000

VRL7	MYCL	NM_001033082.2	c.1175C>G	p.T392S	1.000
VRL7	HSD3B1	NM_000862.2	c.1100C>A	p.T367N	1.000
VRL7	NOTCH2	NM_024408.3	c.57C>G	p.C19W	0.500
VRL7	BTG2	NM_006763.2	c.457G>A	p.V153M	0.500
VRL7	PARP1	NM_001618.3	c.2285T>C	p.V762A	1.000
VRL7	PARP1	NM_001618.3	c.14C>G	p.S5W	0.500
VRL7	TET1	NM_030625.2	c.485A>G	p.D162G	0.500
VRL7	TET1	NM_030625.2	c.1460C>T	p.S487L	0.500
VRL7	TET1	NM_030625.2	c.3369A>G	p.I1123M	1.000
VRL7	BMPR1A	NM_004329.2	c.4C>A	p.P2T	1.000
VRL7	RPS6KA4	NM_003942.2	c.2272T>G	p.S758A	0.500
VRL7	RPS6KB2	NM_003952.2	c.1259C>T	p.A420V	0.500
VRL7	INPPL1	NM_001567.3	c.3248C>G	p.A1083G	0.500
VRL7	ATM	NM_000051.3	c.5948A>G	p.N1983S	1.000
VRL7	KDM5A	NM_001042603.2	c.2594T>C	p.M865T	0.500
VRL7	GLI1	NM_005269.2	c.2798G>A	p.G933D	1.000
VRL7	GLI1	NM_005269.2	c.3298G>C	p.E1100Q	1.000
VRL7	SH2B3	NM_005475.2	c.784T>C	p.W262R	1.000
VRL7	HNF1A	NM_000545.6	c.1720A>G	p.S574G	1.000
VRL7	LATS2	NM_014572.2	c.1087G>A	p.G363S	1.000
VRL7	LATS2	NM_014572.2	c.608C>T	p.A203V	0.500
VRL7	FLT3	NM_004119.2	c.680C>T	p.T227M	1.000
VRL7	FLT3	NM_004119.2	c.20A>G	p.D7G	0.500
VRL7	BRCA2	NM_000059.3	c.1114A>C	p.N372H	0.500
VRL7	BRCA2	NM_000059.3	c.7397T>C	p.V2466A	1.000
VRL7	FOXO1	NM_002015.3	c.244G>A	p.D82N	0.500
VRL7	DIS3	NM_014953.4	c.977C>G	p.T326R	0.500
VRL7	IRS2	NM_003749.2	c.3170G>A	p.G1057D	0.500
VRL7	CUL4A	NM_001008895.2	c.1931A>G	p.K644R	0.500
VRL7	BCL2L2	NM_004050.4	c.398A>G	p.Q133R	1.000
VRL7	FOXA1	NM_004496.3	c.247G>A	p.A83T	0.500
VRL7	MLH3	NM_001040108.1	c.2531C>T	p.P844L	1.000
VRL7	MLH3	NM_001040108.1	c.2476A>G	p.N826D	1.000
VRL7	TSHR	NM_000369.2	c.2181G>C	p.E727D	0.500
VRL7	LTK	NM_002344.5	c.1817G>A	p.R606Q	0.500
VRL7	TP53BP1	NM_001141980.1	c.3535A>G	p.I1179V	0.500
VRL7	TP53BP1	NM_001141980.1	c.3421A>C	p.K1141Q	0.500
VRL7	TP53BP1	NM_001141980.1	c.1249G>A	p.G417S	0.500
VRL7	TP53BP1	NM_001141980.1	c.1074C>G	p.D358E	0.500
VRL7	CD276	NM_001024736.1	c.290C>T	p.P97L	0.500
VRL7	CD276	NM_001024736.1	c.331C>A	p.R111S	0.500
VRL7	CD276	NM_001024736.1	c.479C>T	p.T160M	0.500
VRL7	CD276	NM_001024736.1	c.800G>A	p.R267H	0.500
VRL7	CD276	NM_001024736.1	c.835G>A	p.A279T	0.500
VRL7	BLM	NM_000057.3	c.3961G>A	p.V1321I	0.500
VRL7	ZFHX3	NM_006885.3	c.10554_10556del	p.G3527del	0.500
VRL7	ZFHX3	NM_006885.3	c.4428G>A	p.M1476I	0.500
VRL7	ZFHX3	NM_006885.3	c.2915C>T	p.S972L	0.500
VRL7	ZFHX3	NM_006885.3	c.2330T>C	p.V777A	1.000
VRL7	ANKRD11	NM_013275.5	c.6787C>T	p.P2263S	0.500
VRL7	ANKRD11	NM_013275.5	c.6067G>C	p.A2023P	0.500
VRL7	FANCA	NM_000135.2	c.1501G>A	p.G501S	0.500
VRL7	AURKB	NM_001284526.1	c.896T>C	p.M299T	1.000
VRL7	ERBB2	NM_004448.3	c.3508C>G	p.P1170A	0.500
VRL7	BRCA1	NM_007294.3	c.4837A>G	p.S1613G	0.500
VRL7	RNF43	NM_017763.5	c.1252C>A	p.L418M	0.500

VRL7	RNF43	NM_017763.5	c.350G>A	p.R117H	0.500
VRL7	RNF43	NM_017763.5	c.139A>G	p.I47V	0.500
VRL7	BRIP1	NM_032043.2	c.2755T>C	p.S919P	1.000
VRL7	AXIN2	NM_004655.3	c.148C>T	p.P50S	1.000
VRL7	TCF3	NM_001136139.2	c.1475C>T	p.A492V	0.500
VRL7	PTPRS	NM_002850.3	c.4369T>C	p.C1457R	1.000
VRL7	DNMT1	NM_001130823.2	c.979A>G	p.I327V	0.500
VRL7	NOTCH3	NM_000435.2	c.6668C>T	p.A2223V	1.000
VRL7	PIK3R2	NM_005027.3	c.700A>C	p.S234R	1.000
VRL7	PIK3R2	NM_005027.3	c.937T>C	p.S313P	1.000
VRL7	KMT2B	NM_014727.2	c.3059dup	p.R1021Pfs*14	1.000
VRL7	AXL	NM_021913.4	c.796A>G	p.N266D	1.000
VRL7	CD3EAP	NM_001297590.1	c.1516C>A	p.Q506K	0.500
VRL7	POLD1	NM_001308632.1	c.356G>A	p.R119H	0.500
VRL7	ALK	NM_004304.4	c.4587C>G	p.D1529E	1.000
VRL7	ALK	NM_004304.4	c.4472A>G	p.K1491R	1.000
VRL7	ALK	NM_004304.4	c.4381A>G	p.I1461V	1.000
VRL7	MSH2	NM_000251.2	c.1255C>A	p.Q419K	0.500
VRL7	MSH2	NM_000251.2	c.1886A>G	p.Q629R	0.500
VRL7	MERTK	NM_006343.2	c.1397G>A	p.R466K	0.500
VRL7	MERTK	NM_006343.2	c.1552A>G	p.I518V	0.500
VRL7	LRP1B	NM_018557.2	c.143A>G	p.Q48R	0.500
VRL7	CTLA4	NM_005214.4	c.49A>G	p.T17A	0.500
VRL7	PAK5	NM_020341.3	c.1004G>C	p.R335P	0.500
VRL7	ASXL1	NM_015338.5	c.2444T>C	p.L815P	1.000
VRL7	PTPRT	NM_133170.3	c.85G>C	p.A29P	1.000
VRL7	AURKA	NM_003600.3	c.169A>G	p.I57V	1.000
VRL7	AURKA	NM_003600.3	c.91T>A	p.F31I	1.000
VRL7	TMPRSS2	NM_005656.3	c.478G>A	p.V160M	0.500
VRL7	ICOSLG	NM_001283050.1	c.382G>A	p.V128I	0.500
VRL7	MLH1	NM_000249.3	c.1151T>A	p.V384D	0.500
VRL7	SETD2	NM_014159.6	c.3240G>A	p.M1080I	1.000
VRL7	EPHA3	NM_005233.5	c.2770T>C	p.W924R	0.500
VRL7	ATR	NM_001184.3	c.632T>C	p.M211T	0.500
VRL7	WWTR1	NM_015472.4	c.1199_1200insTTAA	p.L400Lfs*?	0.500
VRL7	KDR	NM_002253.2	c.1416A>T	p.Q472H	0.500
VRL7	ABRAXAS1	NM_139076.2	c.1042G>A	p.A348T	1.000
VRL7	TET2	NM_001127208.2	c.3116C>T	p.S1039L	0.500
VRL7	TET2	NM_001127208.2	c.5162T>G	p.L1721W	0.500
VRL7	FAT1	NM_005245.3	c.12177G>C	p.K4059N	1.000
VRL7	FAT1	NM_005245.3	c.10660T>G	p.S3554A	0.500
VRL7	FAT1	NM_005245.3	c.3190A>G	p.R1064G	1.000
VRL7	FAT1	NM_005245.3	c.2584G>C	p.V862L	1.000
VRL7	FAT1	NM_005245.3	c.1842C>G	p.F614L	1.000
VRL7	FAT1	NM_005245.3	c.392C>T	p.A131V	1.000
VRL7	DROSHA	NM_013235.4	c.962C>T	p.S321L	0.500
VRL7	IL7R	NM_002185.3	c.412G>A	p.V138I	0.500
VRL7	IL7R	NM_002185.3	c.1241C>T	p.T414M	0.500
VRL7	RICTOR	NM_001285439.1	c.2510C>T	p.S837F	0.500
VRL7	MAP3K1	NM_005921.1	c.2416G>A	p.D806N	0.500
VRL7	MAP3K1	NM_005921.1	c.2716G>A	p.V906I	1.000
VRL7	MAP3K1	NM_005921.1	c.2845_2847del	p.T949del	0.500
VRL7	MSH3	NM_002439.4	c.235A>G	p.I79V	1.000
VRL7	MSH3	NM_002439.4	c.2846A>G	p.Q949R	1.000
VRL7	MSH3	NM_002439.4	c.3133G>A	p.A1045T	1.000
VRL7	APC	NM_000038.5	c.5465T>A	p.V1822D	1.000

VRL7	CSF1R	NM_005211.3	c.1085A>G	p.H362R	0.500
VRL7	FGFR4	NM_002011.4	c.28G>A	p.V10I	1.000
VRL7	FGFR4	NM_002011.4	c.407C>T	p.P136L	1.000
VRL7	NSD1	NM_022455.4	c.1840G>T	p.V614L	1.000
VRL7	NSD1	NM_022455.4	c.2176T>C	p.S726P	1.000
VRL7	FLT4	NM_182925.4	c.2670C>G	p.H890Q	0.500
VRL7	MDC1	NM_014641.2	c.803G>A	p.R268K	1.000
VRL7	NOTCH4	NM_004557.3	c.349A>C	p.K117Q	1.000
VRL7	NOTCH4	NM_004557.3	c.45_47del	p.L16del	0.500
VRL7	CDKN1A	NM_001291549.1	c.83A>G	p.D28G	0.500
VRL7	CCND3	NM_001760.4	c.775T>G	p.S259A	0.500
VRL7	ROS1	NM_002944.2	c.521C>A	p.A174E	0.500
VRL7	PMS2	NM_000535.6	c.1621A>G	p.K541E	1.000
VRL7	PMS2	NM_000535.6	c.1454C>A	p.T485K	0.500
VRL7	PMS2	NM_000535.6	c.59G>A	p.R20Q	0.500
VRL7	EGFR	NM_005228.3	c.1562G>A	p.R521K	1.000
VRL7	KMT2C	NM_170606.2	c.2963G>T	p.C988F	0.500
VRL7	KMT2C	NM_170606.2	c.2512G>A	p.G838S	0.500
VRL7	KMT2C	NM_170606.2	c.2447dup	p.Y816*	0.500
VRL7	PRDM14	NM_024504.3	c.730A>G	p.K244E	0.500
VRL7	NBN	NM_002485.4	c.1809C>A	p.F603L	0.500
VRL7	NBN	NM_002485.4	c.553G>C	p.E185Q	0.500
VRL7	RECQL4	NM_004260.3	c.274T>C	p.S92P	0.500
VRL7	PDCD1LG2	NM_025239.3	c.686T>C	p.F229S	1.000
VRL7	PTPRD	NM_002839.3	c.2983C>T	p.R995C	0.500
VRL7	PTPRD	NM_002839.3	c.1339C>G	p.Q447E	0.500
VRL7	MTAP	NM_002451.3	c.166G>A	p.V56I	0.500
VRL7	PTCH1	NM_000264.3	c.3944C>T	p.P1315L	0.500
VRL7	PTCH1	NM_000264.3	c.3913G>T	p.D1305Y	0.500
VRL7	TSC1	NM_000368.4	c.965T>C	p.M322T	0.500
VRL7	AR	NM_000044.3	c.234_239del	p.Q79_Q80del	0.500
VRL7	ATRX	NM_000489.4	c.2785C>G	p.Q929E	1.000
VRL7	BCORL1	NM_001184772.2	c.331T>C	p.F111L	1.000
VRL8	SDHB	NM_003000.2	c.186A>C	p.E62D	0.439
VRL8	HIST2H3D	NM_001123375.2	c.176C>T	p.T59M	0.009
VRL8	BTG2	NM_006763.2	c.27G>A	p.M9I	0.323
VRL8	BTG2	NM_006763.2	c.86G>A	p.C29Y	0.293
VRL8	BTG2	NM_006763.2	c.440G>A	p.S147N	0.336
VRL8	MEN1	NM_000244.3	c.1475C>T	p.S492F	0.487
VRL8	KMT2A	NM_001197104.1	c.2798G>A	p.R933Q	0.468
VRL8	ETV6	NM_001987.4	c.17C>T	p.A6V	0.345
VRL8	ETV6	NM_001987.4	c.26G>A	p.S9N	0.343
VRL8	PTPN11	NM_002834.3	c.823A>G	p.N275D	0.436
VRL8	FOXO1	NM_002015.3	c.580G>A	p.V194M	0.476
VRL8	NFKBIA	NM_020529.2	c.123G>C	p.E41D	0.433
VRL8	RAD51	NM_133487.3	c.942A>C	p.K314N	0.480
VRL8	SOCS1	NM_003745.1	c.412G>C	p.D138H	0.426
VRL8	SOCS1	NM_003745.1	c.333C>G	p.C111W	0.451
VRL8	ANKRD11	NM_013275.5	c.1306G>A	p.G436S	0.539
VRL8	ERBB4	NM_005235.2	c.775G>A	p.V259I	0.331
VRL8	ZNF217	NM_006526.2	c.1312G>T	p.E438*	0.474
VRL8	MDC1	NM_014641.2	c.4551_4561delinsCAGGACAAATA	p.K1519T	0.006
VRL8	PIM1	NM_002648.3	c.79C>G	p.P27A	0.961
VRL8	PIM1	NM_002648.3	c.111G>C	p.Q37H	0.952
VRL8	PIM1	NM_002648.3	c.124C>T	p.P42S	0.951

VRL8	PIM1	NM_002648.3	c.176C>T	p.S59F	0.352
VRL8	PIM1	NM_002648.3	c.241C>T	p.P81S	0.453
VRL8	PIM1	NM_002648.3	c.253C>G	p.R85G	0.012
VRL8	PIM1	NM_002648.3	c.291C>G	p.S97R	0.475
VRL8	PIM1	NM_002648.3	c.296G>C	p.G99A	0.471
VRL8	PIM1	NM_002648.3	c.316C>T	p.L106F	0.458
VRL8	PIM1	NM_002648.3	c.376G>A	p.V126M	0.470
VRL8	PIM1	NM_002648.3	c.385C>T	p.L129F	0.486
VRL8	PIM1	NM_002648.3	c.544C>T	p.L182F	0.453
VRL8	PIM1	NM_002648.3	c.550C>G	p.L184V	0.450
VRL8	PIM1	NM_002648.3	c.607G>A	p.G203R	0.025
VRL8	KMT2C	NM_170606.2	c.2926G>T	p.A976S	0.059
VRL8	MYC	NM_002467.4	c.3_4delinsAA	p.D2N	0.462
VRL8	MYC	NM_002467.4	c.147G>T	p.Q49H	0.462
VRL8	PTPRD	NM_002839.3	c.2432G>T	p.R811L	0.028
VRL8	PTCH1	NM_000264.3	c.14G>C	p.G5A	0.341
VRL8	BCOR	NM_001123385.1	c.1796G>C	p.G599A	0.953
VRL8	MTOR	NM_004958.3	c.6515T>C	p.L2172S	0.500
VRL8	ID3	NM_002167.4	c.313A>G	p.T105A	1.000
VRL8	MYCL	NM_001033082.2	c.1175C>G	p.T392S	1.000
VRL8	MUTYH	NM_001128425.1	c.1014G>C	p.Q338H	1.000
VRL8	NOTCH2	NM_024408.3	c.57C>G	p.C19W	0.500
VRL8	PARP1	NM_001618.3	c.2285T>C	p.V762A	0.500
VRL8	TET1	NM_030625.2	c.577T>A	p.S193T	0.500
VRL8	TET1	NM_030625.2	c.767C>T	p.A256V	0.500
VRL8	TET1	NM_030625.2	c.3053A>G	p.N1018S	0.500
VRL8	TET1	NM_030625.2	c.3369A>G	p.I1123M	0.500
VRL8	BMPR1A	NM_004329.2	c.4C>A	p.P2T	1.000
VRL8	TCF7L2	NM_001146274.1	c.1447C>A	p.P483T	0.500
VRL8	RPS6KA4	NM_003942.2	c.2272T>G	p.S758A	0.500
VRL8	INPPL1	NM_001567.3	c.909G>C	p.K303N	0.500
VRL8	ATM	NM_000051.3	c.5948A>G	p.N1983S	1.000
VRL8	KDM5A	NM_001042603.2	c.2594T>C	p.M865T	1.000
VRL8	RAD52	NM_134424.3	c.1037C>A	p.S346*	0.500
VRL8	PIK3C2G	NM_001288772.1	c.385_387del	p.P129del	0.500
VRL8	PIK3C2G	NM_001288772.1	c.437C>T	p.P146L	0.500
VRL8	H3F3C	NM_001013699.2	c.116A>C	p.H39P	0.500
VRL8	KMT2D	NM_003482.3	c.12028T>C	p.S4010P	0.500
VRL8	KMT2D	NM_003482.3	c.248G>A	p.R83Q	0.500
VRL8	GLI1	NM_005269.2	c.2798G>A	p.G933D	0.500
VRL8	GLI1	NM_005269.2	c.3298G>C	p.E1100Q	0.500
VRL8	SH2B3	NM_005475.2	c.724C>T	p.P242S	0.500
VRL8	SH2B3	NM_005475.2	c.784T>C	p.W262R	1.000
VRL8	HNF1A	NM_000545.6	c.79A>C	p.I27L	0.500
VRL8	HNF1A	NM_000545.6	c.1460G>A	p.S487N	1.000
VRL8	HNF1A	NM_000545.6	c.1720A>G	p.S574G	1.000
VRL8	LATS2	NM_014572.2	c.1087G>A	p.G363S	1.000
VRL8	LATS2	NM_014572.2	c.971C>T	p.A324V	0.500
VRL8	FLT3	NM_004119.2	c.680C>T	p.T227M	1.000
VRL8	FLT3	NM_004119.2	c.20A>G	p.D7G	1.000
VRL8	BRCA2	NM_000059.3	c.7397T>C	p.V2466A	1.000
VRL8	DIS3	NM_014953.4	c.977C>G	p.T326R	0.500
VRL8	DIS3	NM_014953.4	c.806A>G	p.N269S	0.500
VRL8	IRS2	NM_003749.2	c.3170G>A	p.G1057D	0.500
VRL8	BCL2L2	NM_004050.4	c.398A>G	p.Q133R	1.000
VRL8	FOXA1	NM_004496.3	c.247G>A	p.A83T	0.500

VRL8	MLH3	NM_001040108.1	c.2476A>G	p.N826D	1.000
VRL8	TSHR	NM_000369.2	c.2181G>C	p.E727D	1.000
VRL8	MGA	NM_001164273.1	c.5098G>A	p.V1700M	0.500
VRL8	ZFHX3	NM_006885.3	c.10554_10556del	p.G3527del	0.500
VRL8	ZFHX3	NM_006885.3	c.10325C>T	p.P3442L	0.500
VRL8	ZFHX3	NM_006885.3	c.214T>G	p.S72A	0.500
VRL8	ANKRD11	NM_013275.5	c.6176C>A	p.P2059H	0.500
VRL8	ANKRD11	NM_013275.5	c.4912C>G	p.P1638A	0.500
VRL8	FANCA	NM_000135.2	c.2426G>A	p.G809D	1.000
VRL8	FANCA	NM_000135.2	c.1501G>A	p.G501S	1.000
VRL8	FANCA	NM_000135.2	c.796A>G	p.T266A	1.000
VRL8	AURKB	NM_001284526.1	c.896T>C	p.M299T	0.500
VRL8	ERBB2	NM_004448.3	c.1963A>G	p.I655V	0.500
VRL8	ERBB2	NM_004448.3	c.3508C>G	p.P1170A	0.500
VRL8	BRCA1	NM_007294.3	c.4837A>G	p.S1613G	0.500
VRL8	BRCA1	NM_007294.3	c.3548A>G	p.K1183R	0.500
VRL8	BRCA1	NM_007294.3	c.3113A>G	p.E1038G	0.500
VRL8	BRCA1	NM_007294.3	c.2612C>T	p.P871L	0.500
VRL8	RNF43	NM_017763.5	c.1252C>A	p.L418M	0.500
VRL8	RNF43	NM_017763.5	c.139A>G	p.I47V	0.500
VRL8	BRIP1	NM_032043.2	c.2755T>C	p.S919P	0.500
VRL8	AXIN2	NM_004655.3	c.148C>T	p.P50S	0.500
VRL8	GATA6	NM_005257.5	c.43G>C	p.G15R	0.500
VRL8	TCF3	NM_001136139.2	c.1291G>A	p.G431S	0.500
VRL8	DOT1L	NM_032482.2	c.4252G>C	p.V1418L	0.500
VRL8	PTPRS	NM_002850.3	c.4369T>C	p.C1457R	1.000
VRL8	DNMT1	NM_001130823.2	c.979A>G	p.I327V	1.000
VRL8	DNMT1	NM_001130823.2	c.358G>C	p.V120L	1.000
VRL8	NOTCH3	NM_000435.2	c.6668C>T	p.A2223V	0.500
VRL8	PIK3R2	NM_005027.3	c.700A>C	p.S234R	1.000
VRL8	PIK3R2	NM_005027.3	c.937T>C	p.S313P	1.000
VRL8	KMT2B	NM_014727.2	c.3059dup	p.R1021Pfs*14	1.000
VRL8	AXL	NM_021913.4	c.796A>G	p.N266D	1.000
VRL8	CD3EAP	NM_001297590.1	c.1516C>A	p.Q506K	0.500
VRL8	ALK	NM_004304.4	c.4587C>G	p.D1529E	0.500
VRL8	ALK	NM_004304.4	c.4472A>G	p.K1491R	0.500
VRL8	ALK	NM_004304.4	c.4381A>G	p.I1461V	1.000
VRL8	EPAS1	NM_001430.4	c.2296A>C	p.T766P	0.500
VRL8	EPCAM	NM_002354.2	c.344T>C	p.M115T	1.000
VRL8	MSH6	NM_000179.2	c.116G>A	p.G39E	0.500
VRL8	CTLA4	NM_005214.4	c.49A>G	p.T17A	0.500
VRL8	ERBB4	NM_005235.2	c.1972A>T	p.I658F	0.500
VRL8	BARD1	NM_000465.3	c.1134G>C	p.R378S	0.500
VRL8	CUL3	NM_001257198.1	c.1717G>A	p.V573I	0.500
VRL8	PDCD1	NM_005018.2	c.644C>T	p.A215V	0.500
VRL8	ASXL1	NM_015338.5	c.2444T>C	p.L815P	1.000
VRL8	PTPRT	NM_133170.3	c.85G>C	p.A29P	1.000
VRL8	NCOA3	NM_181659.2	c.3789_3791del	p.Q1276del	0.500
VRL8	AURKA	NM_003600.3	c.169A>G	p.I57V	1.000
VRL8	AURKA	NM_003600.3	c.91T>A	p.F31I	0.500
VRL8	RTEL1	NM_001283009.1	c.3126A>C	p.Q1042H	0.500
VRL8	TMPRSS2	NM_005656.3	c.478G>A	p.V160M	0.500
VRL8	SETD2	NM_014159.6	c.5885C>T	p.P1962L	1.000
VRL8	GATA2	NM_032638.4	c.490G>A	p.A164T	0.500
VRL8	ATR	NM_001184.3	c.632T>C	p.M211T	0.500
VRL8	WWTR1	NM_015472.4	c.1199_1200insTTAA	p.L400Lfs*?	0.500

VRL8	FGFR3	NM_000142.4	c.193G>A	p.G65R	0.500
VRL8	ABRAXAS1	NM_139076.2	c.1042G>A	p.A348T	1.000
VRL8	FAT1	NM_005245.3	c.12177G>C	p.K4059N	1.000
VRL8	FAT1	NM_005245.3	c.10930_10931delinsAT	p.A3644M	0.500
VRL8	FAT1	NM_005245.3	c.10660T>G	p.S3554A	0.500
VRL8	FAT1	NM_005245.3	c.8798A>C	p.Q2933P	0.500
VRL8	FAT1	NM_005245.3	c.7105A>C	p.T2369P	0.500
VRL8	FAT1	NM_005245.3	c.6822C>G	p.D2274E	0.500
VRL8	FAT1	NM_005245.3	c.4690G>A	p.A1564T	0.500
VRL8	FAT1	NM_005245.3	c.3818A>G	p.H1273R	0.500
VRL8	RICTOR	NM_001285439.1	c.2510C>T	p.S837F	1.000
VRL8	MAP3K1	NM_005921.1	c.2416G>A	p.D806N	0.500
VRL8	MAP3K1	NM_005921.1	c.2716G>A	p.V906I	1.000
VRL8	MAP3K1	NM_005921.1	c.2845_2847del	p.T949del	0.500
VRL8	MSH3	NM_002439.4	c.199_207del	p.P67_P69del	0.500
VRL8	MSH3	NM_002439.4	c.235A>G	p.I79V	1.000
VRL8	MSH3	NM_002439.4	c.2846A>G	p.Q949R	1.000
VRL8	MSH3	NM_002439.4	c.3133G>A	p.A1045T	1.000
VRL8	APC	NM_000038.5	c.5465T>A	p.V1822D	1.000
VRL8	RAD50	NM_005732.3	c.511G>T	p.A171S	0.500
VRL8	CSF1R	NM_005211.3	c.1085A>G	p.H362R	0.500
VRL8	FGFR4	NM_002011.4	c.28G>A	p.V10I	0.500
VRL8	FGFR4	NM_002011.4	c.407C>T	p.P136L	1.000
VRL8	FGFR4	NM_002011.4	c.535A>G	p.T179A	0.500
VRL8	FGFR4	NM_002011.4	c.1162G>A	p.G388R	0.500
VRL8	NSD1	NM_022455.4	c.1840G>T	p.V614L	0.500
VRL8	NSD1	NM_022455.4	c.2176T>C	p.S726P	0.500
VRL8	HIST1H1C	NM_005319.3	c.53C>T	p.A18V	1.000
VRL8	NOTCH4	NM_004557.3	c.349A>C	p.K117Q	1.000
VRL8	NOTCH4	NM_004557.3	c.36_47del	p.L13_L16del	0.500
VRL8	TAP1	NM_000593.5	c.2090A>G	p.D697G	1.000
VRL8	TAP1	NM_000593.5	c.1177A>G	p.I393V	1.000
VRL8	CDKN1A	NM_001291549.1	c.83A>G	p.D28G	1.000
VRL8	PIM1	NM_002648.3	c.403G>A	p.E135K	1.000
VRL8	PMS2	NM_000535.6	c.1621A>G	p.K541E	1.000
VRL8	PMS2	NM_000535.6	c.1454C>A	p.T485K	0.500
VRL8	PMS2	NM_000535.6	c.1408C>T	p.P470S	0.500
VRL8	ETV1	NM_004956.4	c.298A>G	p.S100G	1.000
VRL8	EGFR	NM_005228.3	c.1562G>A	p.R521K	0.500
VRL8	KMT2C	NM_170606.2	c.2963G>T	p.C988F	0.500
VRL8	KMT2C	NM_170606.2	c.2512G>A	p.G838S	0.500
VRL8	KMT2C	NM_170606.2	c.2447dup	p.Y816*	0.500
VRL8	PREX2	NM_024870.3	c.4463C>T	p.S1488L	0.500
VRL8	NBN	NM_002485.4	c.553G>C	p.E185Q	1.000
VRL8	RECQL4	NM_004260.3	c.3014G>A	p.R1005Q	0.500
VRL8	RECQL4	NM_004260.3	c.801G>C	p.E267D	0.500
VRL8	RECQL4	NM_004260.3	c.274T>C	p.S92P	1.000
VRL8	PDCD1LG2	NM_025239.3	c.686T>C	p.F229S	1.000
VRL8	PTPRD	NM_002839.3	c.1339C>G	p.Q447E	0.500
VRL8	TSC1	NM_000368.4	c.965T>C	p.M322T	0.500
VRL8	AR	NM_000044.3	c.228_239del	p.Q77_Q80del	0.500
VRL8	BCORL1	NM_001184772.2	c.331T>C	p.F111L	1.000
VRL9	KMT2D	NM_003482.3	c.14190G>A	p.W4730*	0.492
VRL9	KMT2D	NM_003482.3	c.12408G>T	p.Q4136H	0.471
VRL9	BTG1	NM_001731.2	c.129C>G	p.S43R	0.495
VRL9	MLH3	NM_001040108.1	c.1755dup	p.E586Rfs*3	0.015

VRL9	ANKRD11	NM_013275.5	c.6792del	p.A2265Pfs*72	0.467
VRL9	TP53	NM_000546.5	c.841G>C	p.D281H	0.493
VRL9	CD79B	NM_001039933.2	c.589T>G	p.Y197D	0.463
VRL9	GNA13	NM_006572.5	c.1004_1022del	p.R335Pfs*22	0.412
VRL9	NF2	NM_000268.3	c.772T>C	p.W258R	0.005
VRL9	MYD88	NM_002468.4	c.794T>C	p.L265P	0.488
VRL9	ATR	NM_001184.3	c.2320dup	p.I774Nfs*3	0.374
VRL9	NSD2	NM_133330.2	c.4027_4028dup	p.E1344Qfs*49	0.017
VRL9	FBXW7	NM_033632.3	c.916C>T	p.Q306*	0.327
VRL9	CTNNA1	NM_001903.4	c.661C>T	p.L221F	0.003
VRL9	HIST1H3H	NM_003536.2	c.181_191del	p.L61Kfs*10	0.449
VRL9	MDC1	NM_014641.2	c.4831C>A	p.P1611T	0.002
VRL9	PIM1	NM_002648.3	c.29C>T	p.A10V	0.491
VRL9	PIM1	NM_002648.3	c.316C>T	p.L106F	0.484
VRL9	PIM1	NM_002648.3	c.543G>C	p.E181D	0.434
VRL9	PNRC1	NM_006813.2	c.348G>C	p.Q116H	0.486
VRL9	ID3	NM_002167.4	c.313A>G	p.T105A	1.000
VRL9	MYCL	NM_001033082.2	c.1175C>G	p.T392S	1.000
VRL9	MUTYH	NM_001128425.1	c.1014G>C	p.Q338H	1.000
VRL9	HSD3B1	NM_000862.2	c.1100C>A	p.T367N	1.000
VRL9	NOTCH2	NM_024408.3	c.57C>G	p.C19W	0.500
VRL9	PARP1	NM_001618.3	c.2285T>C	p.V762A	0.500
VRL9	RET	NM_020975.4	c.406G>A	p.E136K	0.500
VRL9	RET	NM_020975.4	c.2071G>A	p.G691S	0.500
VRL9	TET1	NM_030625.2	c.485A>G	p.D162G	0.500
VRL9	TET1	NM_030625.2	c.3369A>G	p.I1123M	1.000
VRL9	BMPR1A	NM_004329.2	c.4C>A	p.P2T	1.000
VRL9	FGFR2	NM_000141.4	c.17G>C	p.R6P	0.500
VRL9	RPS6KA4	NM_003942.2	c.2272T>G	p.S758A	0.500
VRL9	MEN1	NM_000244.3	c.1636A>G	p.T546A	0.500
VRL9	RPS6KB2	NM_003952.2	c.1259C>T	p.A420V	0.500
VRL9	INPPL1	NM_001567.3	c.3248C>G	p.A1083G	0.500
VRL9	ATM	NM_000051.3	c.5948A>G	p.N1983S	1.000
VRL9	KDM5A	NM_001042603.2	c.2594T>C	p.M865T	0.500
VRL9	PIK3C2G	NM_001288772.1	c.385_387del	p.P129del	0.500
VRL9	PIK3C2G	NM_001288772.1	c.437C>T	p.P146L	0.500
VRL9	SH2B3	NM_005475.2	c.784T>C	p.W262R	1.000
VRL9	HNF1A	NM_000545.6	c.1720A>G	p.S574G	1.000
VRL9	POLE	NM_006231.3	c.5516G>A	p.R1839H	0.500
VRL9	POLE	NM_006231.3	c.755C>T	p.A252V	0.500
VRL9	LATS2	NM_014572.2	c.1431_1436dup	p.P479_A480dup	0.500
VRL9	LATS2	NM_014572.2	c.1087G>A	p.G363S	1.000
VRL9	LATS2	NM_014572.2	c.971C>T	p.A324V	0.500
VRL9	FLT3	NM_004119.2	c.680C>T	p.T227M	0.500
VRL9	FLT3	NM_004119.2	c.20A>G	p.D7G	1.000
VRL9	BRCA2	NM_000059.3	c.7397T>C	p.V2466A	1.000
VRL9	IRS2	NM_003749.2	c.3170G>A	p.G1057D	0.500
VRL9	CUL4A	NM_001008895.2	c.1931A>G	p.K644R	1.000
VRL9	BCL2L2	NM_004050.4	c.398A>G	p.Q133R	1.000
VRL9	FOXA1	NM_004496.3	c.247G>A	p.A83T	1.000
VRL9	MLH3	NM_001040108.1	c.2476A>G	p.N826D	1.000
VRL9	TSHR	NM_000369.2	c.2181G>C	p.E727D	1.000
VRL9	LTK	NM_002344.5	c.125G>A	p.R42Q	0.500
VRL9	TP53BP1	NM_001141980.1	c.3535A>G	p.I1179V	0.500
VRL9	TP53BP1	NM_001141980.1	c.3421A>C	p.K1141Q	1.000
VRL9	TP53BP1	NM_001141980.1	c.1249G>A	p.G417S	1.000

VRL9	TP53BP1	NM_001141980.1	c.1074C>G	p.D358E	1.000
VRL9	CD276	NM_001024736.1	c.290C>T	p.P97L	0.500
VRL9	CD276	NM_001024736.1	c.331C>A	p.R111S	0.500
VRL9	CD276	NM_001024736.1	c.479C>T	p.T160M	0.500
VRL9	CD276	NM_001024736.1	c.800G>A	p.R267H	0.500
VRL9	CD276	NM_001024736.1	c.835G>A	p.A279T	0.500
VRL9	AXIN1	NM_003502.3	c.2215C>T	p.R739C	0.500
VRL9	ZFHX3	NM_006885.3	c.10554_10556del	p.G3527del	0.500
VRL9	FANCA	NM_000135.2	c.3982A>G	p.T1328A	0.500
VRL9	FANCA	NM_000135.2	c.2426G>A	p.G809D	1.000
VRL9	FANCA	NM_000135.2	c.1927C>G	p.P643A	0.500
VRL9	FANCA	NM_000135.2	c.1501G>A	p.G501S	1.000
VRL9	FANCA	NM_000135.2	c.1235C>T	p.A412V	0.500
VRL9	FANCA	NM_000135.2	c.796A>G	p.T266A	1.000
VRL9	TP53	NM_000546.5	c.215C>G	p.P72R	0.500
VRL9	RNF43	NM_017763.5	c.1252C>A	p.L418M	0.500
VRL9	RNF43	NM_017763.5	c.350G>A	p.R117H	0.500
VRL9	RNF43	NM_017763.5	c.139A>G	p.I47V	0.500
VRL9	BRIP1	NM_032043.2	c.2755T>C	p.S919P	0.500
VRL9	BRIP1	NM_032043.2	c.2324A>G	p.N775S	0.500
VRL9	TCF3	NM_001136139.2	c.1475C>T	p.A492V	0.500
VRL9	DOT1L	NM_032482.2	c.4252G>C	p.V1418L	1.000
VRL9	PTPRS	NM_002850.3	c.4369T>C	p.C1457R	1.000
VRL9	DNMT1	NM_001130823.2	c.979A>G	p.I327V	0.500
VRL9	NOTCH3	NM_000435.2	c.4552C>A	p.L1518M	0.500
VRL9	PIK3R2	NM_005027.3	c.700A>C	p.S234R	1.000
VRL9	PIK3R2	NM_005027.3	c.937T>C	p.S313P	1.000
VRL9	KMT2B	NM_014727.2	c.3059dup	p.R1021Pfs*14	1.000
VRL9	KMT2B	NM_014727.2	c.5486C>T	p.P1829L	0.500
VRL9	KMT2B	NM_014727.2	c.7091A>G	p.D2364G	1.000
VRL9	AXL	NM_021913.4	c.796A>G	p.N266D	1.000
VRL9	POLD1	NM_001308632.1	c.356G>A	p.R119H	1.000
VRL9	PPP2R1A	NM_014225.5	c.1052G>A	p.G351D	0.500
VRL9	ALK	NM_004304.4	c.4587C>G	p.D1529E	0.500
VRL9	ALK	NM_004304.4	c.4472A>G	p.K1491R	0.500
VRL9	ALK	NM_004304.4	c.4381A>G	p.I1461V	1.000
VRL9	ALK	NM_004304.4	c.3035C>T	p.T1012M	0.500
VRL9	EPCAM	NM_002354.2	c.344T>C	p.M115T	1.000
VRL9	LRP1B	NM_018557.2	c.143A>G	p.Q48R	0.500
VRL9	BARD1	NM_000465.3	c.1075_1095del	p.L359_P365del	0.500
VRL9	CUL3	NM_001257198.1	c.1717G>A	p.V573I	1.000
VRL9	ASXL1	NM_015338.5	c.2444T>C	p.L815P	1.000
VRL9	PTPRT	NM_133170.3	c.85G>C	p.A29P	0.500
VRL9	NCOA3	NM_181659.2	c.3789_3791del	p.Q1276del	0.500
VRL9	ZNF217	NM_006526.2	c.2666A>G	p.D889G	0.500
VRL9	ZNF217	NM_006526.2	c.2215G>A	p.V739I	0.500
VRL9	AURKA	NM_003600.3	c.169A>G	p.I57V	1.000
VRL9	AURKA	NM_003600.3	c.91T>A	p.F31I	0.500
VRL9	GNAS	NM_080425.3	c.1219A>G	p.T407A	0.500
VRL9	CHEK2	NM_007194.3	c.538C>T	p.R180C	0.500
VRL9	SETD2	NM_014159.6	c.5885C>T	p.P1962L	1.000
VRL9	GATA2	NM_032638.4	c.490G>A	p.A164T	0.500
VRL9	NSD2	NM_133330.2	c.4028dup	p.E1344Rfs*91	0.500
VRL9	PHOX2B	NM_003924.3	c.765_779del	p.A256_A260del	0.500
VRL9	KDR	NM_002253.2	c.1416A>T	p.Q472H	0.500
VRL9	ABRAXAS1	NM_139076.2	c.1042G>A	p.A348T	1.000

VRL9	TET2	NM_001127208.2	c.86C>G	p.P29R	0.500
VRL9	FAT1	NM_005245.3	c.12177G>C	p.K4059N	1.000
VRL9	FAT1	NM_005245.3	c.10660T>G	p.S3554A	0.500
VRL9	FAT1	NM_005245.3	c.8798A>T	p.Q2933L	0.500
VRL9	FAT1	NM_005245.3	c.3818A>G	p.H1273R	0.500
VRL9	FAT1	NM_005245.3	c.3190A>G	p.R1064G	0.500
VRL9	FAT1	NM_005245.3	c.2584G>C	p.V862L	0.500
VRL9	FAT1	NM_005245.3	c.1842C>G	p.F614L	0.500
VRL9	FAT1	NM_005245.3	c.1212T>G	p.S404R	0.500
VRL9	FAT1	NM_005245.3	c.77G>A	p.R26Q	0.500
VRL9	DROSHA	NM_013235.4	c.962C>T	p.S321L	0.500
VRL9	IL7R	NM_002185.3	c.197T>C	p.I66T	0.500
VRL9	IL7R	NM_002185.3	c.412G>A	p.V138I	0.500
VRL9	RICTOR	NM_001285439.1	c.2510C>T	p.S837F	0.500
VRL9	MAP3K1	NM_005921.1	c.2716G>A	p.V906I	0.500
VRL9	MAP3K1	NM_005921.1	c.2845_2847del	p.T949del	0.500
VRL9	MSH3	NM_002439.4	c.235A>G	p.I79V	1.000
VRL9	MSH3	NM_002439.4	c.2846A>G	p.Q949R	1.000
VRL9	MSH3	NM_002439.4	c.3133G>A	p.A1045T	1.000
VRL9	APC	NM_000038.5	c.5465T>A	p.V1822D	1.000
VRL9	CSF1R	NM_005211.3	c.1085A>G	p.H362R	0.500
VRL9	FGFR4	NM_002011.4	c.407C>T	p.P136L	1.000
VRL9	FGFR4	NM_002011.4	c.1162G>A	p.G388R	1.000
VRL9	NSD1	NM_022455.4	c.1840G>T	p.V614L	1.000
VRL9	NSD1	NM_022455.4	c.2176T>C	p.S726P	1.000
VRL9	FLT4	NM_182925.4	c.2670C>G	p.H890Q	0.500
VRL9	NOTCH4	NM_004557.3	c.2824G>A	p.G942R	0.500
VRL9	NOTCH4	NM_004557.3	c.349A>C	p.K117Q	0.500
VRL9	NOTCH4	NM_004557.3	c.45_47dup	p.L16dup	0.500
VRL9	CDKN1A	NM_001291549.1	c.83A>G	p.D28G	0.500
VRL9	CDKN1A	NM_001291549.1	c.195C>A	p.S65R	0.500
VRL9	CCND3	NM_001760.4	c.775T>G	p.S259A	0.500
VRL9	ROS1	NM_002944.2	c.4058A>C	p.Y1353S	1.000
VRL9	PMS2	NM_000535.6	c.1621A>G	p.K541E	1.000
VRL9	PMS2	NM_000535.6	c.59G>A	p.R20Q	0.500
VRL9	ETV1	NM_004956.4	c.298A>G	p.S100G	1.000
VRL9	EGFR	NM_005228.3	c.1562G>A	p.R521K	1.000
VRL9	PIK3CG	NM_002649.3	c.1325C>A	p.S442Y	0.500
VRL9	KMT2C	NM_170606.2	c.2959T>C	p.Y987H	0.500
VRL9	KMT2C	NM_170606.2	c.2512G>A	p.G838S	0.500
VRL9	KMT2C	NM_170606.2	c.2447dup	p.Y816*	0.500
VRL9	NBN	NM_002485.4	c.553G>C	p.E185Q	1.000
VRL9	RECQL4	NM_004260.3	c.3014G>A	p.R1005Q	0.500
VRL9	RECQL4	NM_004260.3	c.801G>C	p.E267D	0.500
VRL9	RECQL4	NM_004260.3	c.274T>C	p.S92P	1.000
VRL9	PDCD1LG2	NM_025239.3	c.686T>C	p.F229S	1.000
VRL9	PTCH1	NM_000264.3	c.3944C>T	p.P1315L	1.000
VRL9	BCOR	NM_001123385.1	c.599C>T	p.T200M	0.500
VRL9	KDM6A	NM_001291415.1	c.2333C>A	p.T778K	0.500
VRL9	AR	NM_000044.3	c.237_239dup	p.Q80dup	0.500
VRL9	BCORL1	NM_001184772.2	c.331T>C	p.F111L	1.000
VRL9	BCORL1	NM_001184772.2	c.2632A>G	p.S878G	0.500
VRL9	CCNQ	NM_152274.4	c.16dup	p.G7Rfs*51	1.000
VRL10	BTG2	NM_006763.2	c.11G>A	p.G4E	0.392
VRL10	BTG2	NM_006763.2	c.71T>A	p.L24Q	0.391
VRL10	BTG2	NM_006763.2	c.117C>G	p.S39R	0.087

VRL10	BTG2	NM_006763.2	c.139A>G	p.T47A	0.339
VRL10	ETV6	NM_001987.4	c.352C>T	p.Q118*	0.313
VRL10	FOXO1	NM_002015.3	c.82C>T	p.P28S	0.484
VRL10	AKT1	NM_005163.2	c.1393C>G	p.R465G	0.503
VRL10	TP53	NM_000546.5	c.773A>C	p.E258A	0.747
VRL10	DNMT3A	NM_022552.4	c.1671C>A	p.C557*	0.380
VRL10	MYD88	NM_002468.4	c.794T>C	p.L265P	0.837
VRL10	TET2	NM_001127208.2	c.4866G>C	p.L1622F	0.477
VRL10	PIM1	NM_002648.3	c.68C>T	p.T23I	0.628
VRL10	PIM1	NM_002648.3	c.70A>G	p.K24E	0.273
VRL10	PIM1	NM_002648.3	c.88G>A	p.E30K	0.558
VRL10	PIM1	NM_002648.3	c.134G>A	p.G45D	0.551
VRL10	PIM1	NM_002648.3	c.183C>G	p.N61K	0.547
VRL10	PIM1	NM_002648.3	c.241C>T	p.P81S	0.854
VRL10	PIM1	NM_002648.3	c.256G>A	p.V86M	0.364
VRL10	PIM1	NM_002648.3	c.264G>A	p.M88I	0.765
VRL10	PIM1	NM_002648.3	c.272T>A	p.V91D	0.494
VRL10	PIM1	NM_002648.3	c.281A>G	p.K94R	0.504
VRL10	PIM1	NM_002648.3	c.373C>T	p.P125S	0.504
VRL10	PIM1	NM_002648.3	c.412G>A	p.A138T	0.346
VRL10	PIM1	NM_002648.3	c.587C>T	p.T196I	0.501
VRL10	PIM1	NM_002648.3	c.600C>G	p.D200E	0.502
VRL10	RAC1	NM_018890.3	c.367C>A	p.H123N	0.408
VRL10	PAX5	NM_016734.2	c.20A>C	p.Y7S	0.439
VRL10	PAX5	NM_016734.2	c.14A>G	p.K5R	0.456
VRL10	ATRX	NM_000489.4	c.4902G>A	p.W1634*	0.393
VRL10	SPEN	NM_015001.2	c.2909C>T	p.A970V	1.000
VRL10	SPEN	NM_015001.2	c.3272T>C	p.L1091P	1.000
VRL10	SPEN	NM_015001.2	c.7078A>G	p.N2360D	1.000
VRL10	ID3	NM_002167.4	c.313A>G	p.T105A	1.000
VRL10	MYCL	NM_001033082.2	c.1175C>G	p.T392S	1.000
VRL10	MPL	NM_005373.2	c.1120A>G	p.T374A	0.500
VRL10	MUTYH	NM_001128425.1	c.1014G>C	p.Q338H	0.500
VRL10	BCL10	NM_003921.4	c.13G>T	p.A5S	0.500
VRL10	HSD3B1	NM_000862.2	c.1100C>A	p.T367N	1.000
VRL10	NOTCH2	NM_024408.3	c.57C>G	p.C19W	0.500
VRL10	NUF2	NM_031423.3	c.715A>C	p.S239R	0.500
VRL10	TET1	NM_030625.2	c.3369A>G	p.I1123M	1.000
VRL10	BMPR1A	NM_004329.2	c.4C>A	p.P2T	1.000
VRL10	TCF7L2	NM_001146274.1	c.1447C>A	p.P483T	0.500
VRL10	MEN1	NM_000244.3	c.1636A>G	p.T546A	1.000
VRL10	RPS6KB2	NM_003952.2	c.1259C>T	p.A420V	0.500
VRL10	ATM	NM_000051.3	c.5948A>G	p.N1983S	1.000
VRL10	KDM5A	NM_001042603.2	c.2594T>C	p.M865T	0.500
VRL10	PIK3C2G	NM_001288772.1	c.385_387del	p.P129del	1.000
VRL10	PIK3C2G	NM_001288772.1	c.386C>G	p.P129R	1.000
VRL10	PIK3C2G	NM_001288772.1	c.437C>T	p.P146L	1.000
VRL10	H3F3C	NM_001013699.2	c.116A>C	p.H39P	0.500
VRL10	KMT2D	NM_003482.3	c.11817_11837del	p.L3940_Q3946del	0.500
VRL10	SH2B3	NM_005475.2	c.784T>C	p.W262R	1.000
VRL10	HNF1A	NM_000545.6	c.79A>C	p.I27L	0.500
VRL10	HNF1A	NM_000545.6	c.1460G>A	p.S487N	1.000
VRL10	HNF1A	NM_000545.6	c.1720A>G	p.S574G	1.000
VRL10	LATS2	NM_014572.2	c.1087G>A	p.G363S	1.000
VRL10	LATS2	NM_014572.2	c.971C>T	p.A324V	0.500
VRL10	FLT3	NM_004119.2	c.680C>T	p.T227M	1.000

VRL10	FLT3	NM_004119.2	c.20A>G	p.D7G	1.000
VRL10	BRCA2	NM_000059.3	c.1114A>C	p.N372H	0.500
VRL10	BRCA2	NM_000059.3	c.7397T>C	p.V2466A	1.000
VRL10	DIS3	NM_014953.4	c.977C>G	p.T326R	0.500
VRL10	IRS2	NM_003749.2	c.3938C>T	p.A1313V	0.500
VRL10	BCL2L2	NM_004050.4	c.398A>G	p.Q133R	1.000
VRL10	FOXA1	NM_004496.3	c.247G>A	p.A83T	1.000
VRL10	MLH3	NM_001040108.1	c.2825C>T	p.T942I	0.500
VRL10	MLH3	NM_001040108.1	c.2476A>G	p.N826D	1.000
VRL10	TSHR	NM_000369.2	c.2181G>C	p.E727D	1.000
VRL10	LTK	NM_002344.5	c.125G>A	p.R42Q	0.500
VRL10	MGA	NM_001164273.1	c.4567C>G	p.P1523A	0.500
VRL10	BLM	NM_000057.3	c.893C>T	p.T298M	0.500
VRL10	IGF1R	NM_000875.4	c.2570A>G	p.N857S	0.500
VRL10	SLX4	NM_032444.2	c.73G>A	p.G25R	0.500
VRL10	SOCS1	NM_003745.1	c.227C>G	p.A76G	0.500
VRL10	SOCS1	NM_003745.1	c.149C>T	p.P50L	0.500
VRL10	ZFHX3	NM_006885.3	c.1378G>C	p.E460Q	0.500
VRL10	ANKRD11	NM_013275.5	c.6176C>A	p.P2059H	0.500
VRL10	ANKRD11	NM_013275.5	c.4912C>G	p.P1638A	0.500
VRL10	FANCA	NM_000135.2	c.2426G>A	p.G809D	1.000
VRL10	FANCA	NM_000135.2	c.1501G>A	p.G501S	1.000
VRL10	FANCA	NM_000135.2	c.796A>G	p.T266A	1.000
VRL10	TP53	NM_000546.5	c.215C>G	p.P72R	0.500
VRL10	ERBB2	NM_004448.3	c.3508C>G	p.P1170A	0.500
VRL10	BRCA1	NM_007294.3	c.4837A>G	p.S1613G	1.000
VRL10	BRCA1	NM_007294.3	c.3548A>G	p.K1183R	1.000
VRL10	BRCA1	NM_007294.3	c.3113A>G	p.E1038G	1.000
VRL10	BRCA1	NM_007294.3	c.2612C>T	p.P871L	1.000
VRL10	RNF43	NM_017763.5	c.1252C>A	p.L418M	1.000
VRL10	RNF43	NM_017763.5	c.139A>G	p.I47V	1.000
VRL10	BRIP1	NM_032043.2	c.2755T>C	p.S919P	0.500
VRL10	AXIN2	NM_004655.3	c.148C>T	p.P50S	1.000
VRL10	TCF3	NM_001136139.2	c.1475C>T	p.A492V	1.000
VRL10	DOT1L	NM_032482.2	c.4156G>A	p.G1386S	0.500
VRL10	DOT1L	NM_032482.2	c.4252G>C	p.V1418L	0.500
VRL10	PTPRS	NM_002850.3	c.4369T>C	p.C1457R	1.000
VRL10	DNMT1	NM_001130823.2	c.979A>G	p.I327V	1.000
VRL10	DNMT1	NM_001130823.2	c.290A>G	p.H97R	0.500
VRL10	NOTCH3	NM_000435.2	c.6668C>T	p.A2223V	0.500
VRL10	PIK3R2	NM_005027.3	c.700A>C	p.S234R	1.000
VRL10	PIK3R2	NM_005027.3	c.937T>C	p.S313P	1.000
VRL10	KMT2B	NM_014727.2	c.3059dup	p.R1021Pfs*14	1.000
VRL10	KMT2B	NM_014727.2	c.7091A>G	p.D2364G	0.500
VRL10	AXL	NM_021913.4	c.796A>G	p.N266D	1.000
VRL10	ALK	NM_004304.4	c.4587C>G	p.D1529E	1.000
VRL10	ALK	NM_004304.4	c.4472A>G	p.K1491R	1.000
VRL10	ALK	NM_004304.4	c.4381A>G	p.I1461V	1.000
VRL10	EPCAM	NM_002354.2	c.344T>C	p.M115T	1.000
VRL10	MSH6	NM_000179.2	c.116G>A	p.G39E	0.500
VRL10	LRP1B	NM_018557.2	c.9877G>A	p.G3293R	0.500
VRL10	LRP1B	NM_018557.2	c.143A>G	p.Q48R	0.500
VRL10	CTLA4	NM_005214.4	c.49A>G	p.T17A	0.500
VRL10	BARD1	NM_000465.3	c.1134G>C	p.R378S	1.000
VRL10	BARD1	NM_000465.3	c.70C>T	p.P24S	1.000
VRL10	ASXL1	NM_015338.5	c.2444T>C	p.L815P	1.000

VRL10	PTPRT	NM_133170.3	c.85G>C	p.A29P	0.500
VRL10	NCOA3	NM_181659.2	c.3789_3791del	p.Q1276del	0.500
VRL10	ZNF217	NM_006526.2	c.1643C>T	p.T548I	0.500
VRL10	AURKA	NM_003600.3	c.169A>G	p.I57V	1.000
VRL10	AURKA	NM_003600.3	c.91T>A	p.F31I	0.500
VRL10	RTEL1	NM_001283009.1	c.3126A>C	p.Q1042H	0.500
VRL10	TGFBR2	NM_003242.5	c.571G>A	p.V191I	1.000
VRL10	SETD2	NM_014159.6	c.3240G>A	p.M1080I	1.000
VRL10	SHQ1	NM_018130.2	c.1466G>A	p.S489N	0.500
VRL10	GATA2	NM_032638.4	c.490G>A	p.A164T	0.500
VRL10	ATR	NM_001184.3	c.632T>C	p.M211T	0.500
VRL10	WWTR1	NM_015472.4	c.1199_1200insTTAA	p.L400Lfs*?	1.000
VRL10	ABRAXAS1	NM_139076.2	c.1042G>A	p.A348T	0.500
VRL10	TET2	NM_001127208.2	c.5284A>G	p.I1762V	0.500
VRL10	FAT1	NM_005245.3	c.13652C>G	p.A4551G	0.500
VRL10	FAT1	NM_005245.3	c.12177G>C	p.K4059N	1.000
VRL10	FAT1	NM_005245.3	c.10226C>T	p.T3409M	0.500
VRL10	FAT1	NM_005245.3	c.3190A>G	p.R1064G	0.500
VRL10	FAT1	NM_005245.3	c.2584G>C	p.V862L	0.500
VRL10	FAT1	NM_005245.3	c.1842C>G	p.F614L	0.500
VRL10	FAT1	NM_005245.3	c.392C>T	p.A131V	0.500
VRL10	TERT	NM_198253.2	c.1154G>A	p.R385H	0.500
VRL10	DROSHA	NM_013235.4	c.962C>T	p.S321L	0.500
VRL10	RICTOR	NM_001285439.1	c.2510C>T	p.S837F	1.000
VRL10	MSH3	NM_002439.4	c.2846A>G	p.Q949R	1.000
VRL10	MSH3	NM_002439.4	c.3133G>A	p.A1045T	1.000
VRL10	APC	NM_000038.5	c.5465T>A	p.V1822D	1.000
VRL10	CSF1R	NM_005211.3	c.1085A>G	p.H362R	0.500
VRL10	FGFR4	NM_002011.4	c.28G>A	p.V10I	0.500
VRL10	FGFR4	NM_002011.4	c.407C>T	p.P136L	1.000
VRL10	FGFR4	NM_002011.4	c.535A>G	p.T179A	0.500
VRL10	FGFR4	NM_002011.4	c.1162G>A	p.G388R	0.500
VRL10	NSD1	NM_022455.4	c.1840G>T	p.V614L	0.500
VRL10	NSD1	NM_022455.4	c.2176T>C	p.S726P	0.500
VRL10	HIST1H1C	NM_005319.3	c.53C>T	p.A18V	1.000
VRL10	MDC1	NM_014641.2	c.5200G>A	p.A1734T	0.500
VRL10	MDC1	NM_014641.2	c.4991C>G	p.T1664S	0.500
VRL10	MDC1	NM_014641.2	c.4618T>C	p.S1540P	0.500
VRL10	MDC1	NM_014641.2	c.3947T>C	p.M1316T	0.500
VRL10	MDC1	NM_014641.2	c.3847C>A	p.P1283T	0.500
VRL10	MDC1	NM_014641.2	c.3797A>C	p.Y1266S	0.500
VRL10	MDC1	NM_014641.2	c.3538T>C	p.S1180P	1.000
VRL10	MDC1	NM_014641.2	c.1157C>T	p.P386L	1.000
VRL10	MDC1	NM_014641.2	c.1111G>A	p.E371K	1.000
VRL10	DDR1	NM_013994.2	c.920G>A	p.R307H	0.500
VRL10	TAP1	NM_000593.5	c.2090A>G	p.D697G	0.500
VRL10	TAP1	NM_000593.5	c.1177A>G	p.I393V	0.500
VRL10	CDKN1A	NM_001291549.1	c.83A>G	p.D28G	0.500
VRL10	CDKN1A	NM_001291549.1	c.195C>A	p.S65R	0.500
VRL10	ROS1	NM_002944.2	c.670C>T	p.P224S	0.500
VRL10	PRKN	NM_004562.2	c.1138G>C	p.V380L	0.500
VRL10	PMS2	NM_000535.6	c.1621A>G	p.K541E	1.000
VRL10	PMS2	NM_000535.6	c.1454C>A	p.T485K	1.000
VRL10	ETV1	NM_004956.4	c.298A>G	p.S100G	0.500
VRL10	PIK3CG	NM_002649.3	c.1325C>A	p.S442Y	0.500
VRL10	KMT2C	NM_170606.2	c.2959T>C	p.Y987H	0.500

VRL10	KMT2C	NM_170606.2	c.2512G>A	p.G838S	0.500
VRL10	KMT2C	NM_170606.2	c.2447dup	p.Y816*	0.500
VRL10	PRDM14	NM_024504.3	c.730A>G	p.K244E	0.500
VRL10	NBN	NM_002485.4	c.553G>C	p.E185Q	0.500
VRL10	RECQL4	NM_004260.3	c.3014G>A	p.R1005Q	1.000
VRL10	RECQL4	NM_004260.3	c.801G>C	p.E267D	0.500
VRL10	RECQL4	NM_004260.3	c.274T>C	p.S92P	1.000
VRL10	PDCD1LG2	NM_025239.3	c.686T>C	p.F229S	1.000
VRL10	MTAP	NM_002451.3	c.166G>A	p.V56I	0.500
VRL10	PTCH1	NM_000264.3	c.3944C>T	p.P1315L	0.500
VRL10	EGFL7	NM_016215.4	c.457G>A	p.V153I	0.500
VRL10	AR	NM_000044.3	c.234_239dup	p.Q79_Q80dup	0.500
VRL10	BCORL1	NM_001184772.2	c.331T>C	p.F111L	1.000
VRL11	BTG2	NM_006763.2	c.235_244del	p.S79Pfs*19	0.466
VRL11	TET1	NM_030625.2	c.3299T>C	p.V1100A	0.677
VRL11	PIK3C2G	NM_001288772.1	c.227A>C	p.H76P	0.331
VRL11	CD79B	NM_001039933.2	c.589T>G	p.Y197D	0.982
VRL11	TGFBR2	NM_003242.5	c.802T>A	p.S268T	0.240
VRL11	MYD88	NM_002468.4	c.794T>C	p.L265P	0.500
VRL11	FOXL2	NM_023067.3	c.1106C>T	p.A369V	0.489
VRL11	EPHA5	NM_001281765.2	c.1441G>T	p.A481S	0.495
VRL11	ABRAXAS1	NM_139076.2	c.1018A>G	p.T340A	0.527
VRL11	CSF1R	NM_005211.3	c.196A>G	p.S66G	0.498
VRL11	IRF4	NM_002460.3	c.207_208delinsCG	p.L70V	0.659
VRL11	PIM1	NM_002648.3	c.4C>T	p.L2F	0.013
VRL11	PIM1	NM_002648.3	c.72G>C	p.K24N	0.416
VRL11	PIM1	NM_002648.3	c.241C>T	p.P81S	0.479
VRL11	PIM1	NM_002648.3	c.290G>A	p.S97N	0.459
VRL11	PIM1	NM_002648.3	c.403G>A	p.E135K	0.049
VRL11	PIM1	NM_002648.3	c.543G>C	p.E181D	0.479
VRL11	PIM1	NM_002648.3	c.549_550delinsAT	p.L184F	0.419
VRL11	PIM1	NM_002648.3	c.553A>G	p.I185V	0.463
VRL11	CARD11	NM_032415.5	c.625G>A	p.A209T	0.688
VRL11	CRLF2	NM_022148.3	c.475G>A	p.E159K	0.502
VRL11	TNFRSF14	NM_003820.3	c.349G>A	p.A117T	0.500
VRL11	ID3	NM_002167.4	c.313A>G	p.T105A	0.500
VRL11	MYCL	NM_001033082.2	c.1175C>G	p.T392S	1.000
VRL11	MUTYH	NM_001128425.1	c.1014G>C	p.Q338H	0.500
VRL11	FAM46C	NM_017709.3	c.201C>G	p.H67Q	0.500
VRL11	HSD3B1	NM_000862.2	c.1100C>A	p.T367N	1.000
VRL11	NOTCH2	NM_024408.3	c.57C>G	p.C19W	0.500
VRL11	NUF2	NM_031423.3	c.715A>C	p.S239R	0.500
VRL11	PARP1	NM_001618.3	c.2285T>C	p.V762A	1.000
VRL11	RET	NM_020975.4	c.2071G>A	p.G691S	0.500
VRL11	TET1	NM_030625.2	c.485A>G	p.D162G	0.500
VRL11	TET1	NM_030625.2	c.577T>A	p.S193T	0.500
VRL11	TET1	NM_030625.2	c.3369A>G	p.I1123M	0.500
VRL11	BMPR1A	NM_004329.2	c.4C>A	p.P2T	0.500
VRL11	RPS6KA4	NM_003942.2	c.2272T>G	p.S758A	1.000
VRL11	MEN1	NM_000244.3	c.1636A>G	p.T546A	1.000
VRL11	RPS6KB2	NM_003952.2	c.1259C>T	p.A420V	0.500
VRL11	INPPL1	NM_001567.3	c.909G>C	p.K303N	0.500
VRL11	ATM	NM_000051.3	c.5948A>G	p.N1983S	1.000
VRL11	KDM5A	NM_001042603.2	c.2594T>C	p.M865T	0.500
VRL11	RAD52	NM_134424.3	c.1037C>A	p.S346*	0.500
VRL11	FGF23	NM_020638.2	c.716C>T	p.T239M	0.500

VRL11	PIK3C2G	NM_001288772.1	c.385_387del	p.P129del	0.500
VRL11	PIK3C2G	NM_001288772.1	c.437C>T	p.P146L	0.500
VRL11	GLI1	NM_005269.2	c.2798G>A	p.G933D	0.500
VRL11	GLI1	NM_005269.2	c.3035G>T	p.G1012V	0.500
VRL11	GLI1	NM_005269.2	c.3298G>C	p.E1100Q	1.000
VRL11	SH2B3	NM_005475.2	c.784T>C	p.W262R	1.000
VRL11	HNF1A	NM_000545.6	c.79A>C	p.I27L	1.000
VRL11	HNF1A	NM_000545.6	c.1460G>A	p.S487N	1.000
VRL11	HNF1A	NM_000545.6	c.1720A>G	p.S574G	1.000
VRL11	LATS2	NM_014572.2	c.1087G>A	p.G363S	1.000
VRL11	LATS2	NM_014572.2	c.971C>T	p.A324V	1.000
VRL11	FLT3	NM_004119.2	c.680C>T	p.T227M	0.500
VRL11	FLT3	NM_004119.2	c.20A>G	p.D7G	0.500
VRL11	BRCA2	NM_000059.3	c.7397T>C	p.V2466A	1.000
VRL11	FOXO1	NM_002015.3	c.244G>A	p.D82N	0.500
VRL11	DIS3	NM_014953.4	c.977C>G	p.T326R	1.000
VRL11	IRS2	NM_003749.2	c.3170G>A	p.G1057D	1.000
VRL11	CUL4A	NM_001008895.2	c.1931A>G	p.K644R	0.500
VRL11	BCL2L2	NM_004050.4	c.398A>G	p.Q133R	1.000
VRL11	FOXA1	NM_004496.3	c.247G>A	p.A83T	1.000
VRL11	MLH3	NM_001040108.1	c.2531C>T	p.P844L	0.500
VRL11	MLH3	NM_001040108.1	c.2476A>G	p.N826D	1.000
VRL11	TSHR	NM_000369.2	c.2181G>C	p.E727D	1.000
VRL11	LTK	NM_002344.5	c.1705C>A	p.R569S	0.500
VRL11	TP53BP1	NM_001141980.1	c.3421A>C	p.K1141Q	1.000
VRL11	TP53BP1	NM_001141980.1	c.1249G>A	p.G417S	1.000
VRL11	TP53BP1	NM_001141980.1	c.1074C>G	p.D358E	1.000
VRL11	CD276	NM_001024736.1	c.290C>T	p.P97L	0.500
VRL11	CD276	NM_001024736.1	c.331C>A	p.R111S	0.500
VRL11	CD276	NM_001024736.1	c.479C>T	p.T160M	0.500
VRL11	CD276	NM_001024736.1	c.800G>A	p.R267H	0.500
VRL11	CD276	NM_001024736.1	c.835G>A	p.A279T	0.500
VRL11	SLX4	NM_032444.2	c.3812C>T	p.S1271F	0.500
VRL11	SLX4	NM_032444.2	c.3662C>T	p.A1221V	0.500
VRL11	SLX4	NM_032444.2	c.3365C>T	p.P1122L	0.500
VRL11	SLX4	NM_032444.2	c.2854_2855delinsAT	p.A952M	0.500
VRL11	SLX4	NM_032444.2	c.2449G>C	p.E817Q	0.500
VRL11	SLX4	NM_032444.2	c.2012T>C	p.L671S	0.500
VRL11	SLX4	NM_032444.2	c.1371T>G	p.N457K	0.500
VRL11	SLX4	NM_032444.2	c.1271C>T	p.A424V	0.500
VRL11	SLX4	NM_032444.2	c.610C>T	p.R204C	0.500
VRL11	ZFHX3	NM_006885.3	c.1378G>C	p.E460Q	0.500
VRL11	ANKRD11	NM_013275.5	c.6868C>T	p.P2290S	0.500
VRL11	ANKRD11	NM_013275.5	c.6176C>A	p.P2059H	0.500
VRL11	ANKRD11	NM_013275.5	c.4912C>G	p.P1638A	0.500
VRL11	FANCA	NM_000135.2	c.3982A>G	p.T1328A	1.000
VRL11	FANCA	NM_000135.2	c.2426G>A	p.G809D	1.000
VRL11	FANCA	NM_000135.2	c.1927C>G	p.P643A	1.000
VRL11	FANCA	NM_000135.2	c.1501G>A	p.G501S	1.000
VRL11	FANCA	NM_000135.2	c.1235C>T	p.A412V	1.000
VRL11	FANCA	NM_000135.2	c.796A>G	p.T266A	1.000
VRL11	TP53	NM_000546.5	c.215C>G	p.P72R	0.500
VRL11	AURKB	NM_001284526.1	c.896T>C	p.M299T	0.500
VRL11	ERBB2	NM_004448.3	c.3508C>G	p.P1170A	0.500
VRL11	RARA	NM_000964.3	c.1299C>A	p.D433E	0.500
VRL11	BRCA1	NM_007294.3	c.4837A>G	p.S1613G	0.500

VRL11	BRCA1	NM_007294.3	c.3548A>G	p.K1183R	0.500
VRL11	BRCA1	NM_007294.3	c.3113A>G	p.E1038G	0.500
VRL11	BRCA1	NM_007294.3	c.2612C>T	p.P871L	0.500
VRL11	RNF43	NM_017763.5	c.350G>A	p.R117H	1.000
VRL11	RNF43	NM_017763.5	c.139A>G	p.I47V	1.000
VRL11	BRIP1	NM_032043.2	c.2755T>C	p.S919P	1.000
VRL11	AXIN2	NM_004655.3	c.148C>T	p.P50S	1.000
VRL11	GATA6	NM_005257.5	c.43G>C	p.G15R	0.500
VRL11	BCL2	NM_000633.2	c.127G>A	p.A43T	0.500
VRL11	TCF3	NM_001136139.2	c.1475C>T	p.A492V	0.500
VRL11	DOT1L	NM_032482.2	c.4252G>C	p.V1418L	0.500
VRL11	PTPRS	NM_002850.3	c.4369T>C	p.C1457R	1.000
VRL11	NOTCH3	NM_000435.2	c.6668C>T	p.A2223V	0.500
VRL11	PIK3R2	NM_005027.3	c.700A>C	p.S234R	1.000
VRL11	PIK3R2	NM_005027.3	c.937T>C	p.S313P	1.000
VRL11	CEBPA	NM_004364.4	c.584_589dup	p.H195_P196dup	0.500
VRL11	KMT2B	NM_014727.2	c.1760C>G	p.P587R	0.500
VRL11	KMT2B	NM_014727.2	c.3059dup	p.R1021Pfs*14	1.000
VRL11	KMT2B	NM_014727.2	c.7091A>G	p.D2364G	1.000
VRL11	AXL	NM_021913.4	c.796A>G	p.N266D	1.000
VRL11	CD3EAP	NM_001297590.1	c.1516C>A	p.Q506K	1.000
VRL11	ALK	NM_004304.4	c.4587C>G	p.D1529E	1.000
VRL11	ALK	NM_004304.4	c.4472A>G	p.K1491R	1.000
VRL11	ALK	NM_004304.4	c.4381A>G	p.I1461V	1.000
VRL11	EPCAM	NM_002354.2	c.344T>C	p.M115T	1.000
VRL11	CTLA4	NM_005214.4	c.49A>G	p.T17A	0.500
VRL11	PDCD1	NM_005018.2	c.644C>T	p.A215V	0.500
VRL11	ASXL1	NM_015338.5	c.2444T>C	p.L815P	1.000
VRL11	PTPRT	NM_133170.3	c.85G>C	p.A29P	0.500
VRL11	NCOA3	NM_181659.2	c.3789_3791del	p.Q1276del	0.500
VRL11	ZNF217	NM_006526.2	c.1643C>T	p.T548I	0.500
VRL11	AURKA	NM_003600.3	c.169A>G	p.I57V	1.000
VRL11	AURKA	NM_003600.3	c.91T>A	p.F31I	0.500
VRL11	RTEL1	NM_001283009.1	c.3126A>C	p.Q1042H	0.500
VRL11	SETD2	NM_014159.6	c.5885C>T	p.P1962L	1.000
VRL11	ATR	NM_001184.3	c.632T>C	p.M211T	0.500
VRL11	KDR	NM_002253.2	c.1416A>T	p.Q472H	0.500
VRL11	ABRAXAS1	NM_139076.2	c.1042G>A	p.A348T	1.000
VRL11	TET2	NM_001127208.2	c.5162T>G	p.L1721W	0.500
VRL11	TET2	NM_001127208.2	c.5284A>G	p.I1762V	0.500
VRL11	FAT1	NM_005245.3	c.12177G>C	p.K4059N	1.000
VRL11	FAT1	NM_005245.3	c.3423G>C	p.E1141D	0.500
VRL11	FAT1	NM_005245.3	c.3190A>G	p.R1064G	1.000
VRL11	FAT1	NM_005245.3	c.2584G>C	p.V862L	1.000
VRL11	FAT1	NM_005245.3	c.1842C>G	p.F614L	1.000
VRL11	FAT1	NM_005245.3	c.1444G>A	p.V482I	0.500
VRL11	FAT1	NM_005245.3	c.1212T>G	p.S404R	0.500
VRL11	FAT1	NM_005245.3	c.392C>T	p.A131V	0.500
VRL11	IL7R	NM_002185.3	c.197T>C	p.I66T	1.000
VRL11	IL7R	NM_002185.3	c.412G>A	p.V138I	1.000
VRL11	RICTOR	NM_001285439.1	c.2510C>T	p.S837F	0.500
VRL11	MAP3K1	NM_005921.1	c.2716G>A	p.V906I	1.000
VRL11	MAP3K1	NM_005921.1	c.2845_2847del	p.T949del	0.500
VRL11	MSH3	NM_002439.4	c.181_189dup	p.A61_P63dup	0.500
VRL11	MSH3	NM_002439.4	c.2846A>G	p.Q949R	1.000
VRL11	MSH3	NM_002439.4	c.3133G>A	p.A1045T	1.000

VRL11	APC	NM_000038.5	c.5465T>A	p.V1822D	1.000
VRL11	CSF1R	NM_005211.3	c.1085A>G	p.H362R	1.000
VRL11	FGFR4	NM_002011.4	c.28G>A	p.V10I	1.000
VRL11	FGFR4	NM_002011.4	c.407C>T	p.P136L	1.000
VRL11	HIST1H1C	NM_005319.3	c.53C>T	p.A18V	1.000
VRL11	NOTCH4	NM_004557.3	c.611C>T	p.P204L	0.500
VRL11	NOTCH4	NM_004557.3	c.349A>C	p.K117Q	0.500
VRL11	TAP1	NM_000593.5	c.2090A>G	p.D697G	0.500
VRL11	TAP1	NM_000593.5	c.1177A>G	p.I393V	0.500
VRL11	CDKN1A	NM_001291549.1	c.195C>A	p.S65R	1.000
VRL11	CCND3	NM_001760.4	c.775T>G	p.S259A	0.500
VRL11	PMS2	NM_000535.6	c.1621A>G	p.K541E	1.000
VRL11	PMS2	NM_000535.6	c.1454C>A	p.T485K	0.500
VRL11	ETV1	NM_004956.4	c.298A>G	p.S100G	1.000
VRL11	EGFR	NM_005228.3	c.1562G>A	p.R521K	0.500
VRL11	PIK3CG	NM_002649.3	c.89C>G	p.A30G	0.500
VRL11	PIK3CG	NM_002649.3	c.2569A>G	p.T857A	0.500
VRL11	KMT2C	NM_170606.2	c.2959T>C	p.Y987H	0.500
VRL11	KMT2C	NM_170606.2	c.2512G>A	p.G838S	0.500
VRL11	PREX2	NM_024870.3	c.4463C>T	p.S1488L	0.500
VRL11	PRDM14	NM_024504.3	c.730A>G	p.K244E	0.500
VRL11	NBN	NM_002485.4	c.553G>C	p.E185Q	0.500
VRL11	RECQL4	NM_004260.3	c.3014G>A	p.R1005Q	1.000
VRL11	RECQL4	NM_004260.3	c.801G>C	p.E267D	1.000
VRL11	RECQL4	NM_004260.3	c.274T>C	p.S92P	1.000
VRL11	PDCD1LG2	NM_025239.3	c.686T>C	p.F229S	1.000
VRL11	PTCH1	NM_000264.3	c.3944C>T	p.P1315L	1.000
VRL11	KDM6A	NM_001291415.1	c.2333C>A	p.T778K	0.500
VRL11	AMER1	NM_152424.3	c.85G>A	p.A29T	0.500
VRL11	AR	NM_000044.3	c.237_239dup	p.Q80dup	0.500
VRL11	ATRX	NM_000489.4	c.2785C>G	p.Q929E	1.000
VRL11	BCORL1	NM_001184772.2	c.331T>C	p.F111L	1.000
VRL11	CCNQ	NM_152274.4	c.16dup	p.G7Rfs*51	1.000
VRL12	PTEN	NM_000314.6	c.25G>A	p.V9I	0.011
VRL12	BTG1	NM_001731.2	c.3G>A	p.M1?	0.020
VRL12	LRP1B	NM_018557.2	c.3912G>A	p.W1304*	0.230
VRL12	MYD88	NM_002468.4	c.794T>C	p.L265P	0.645
VRL12	FOXL2	NM_023067.3	c.937G>A	p.G313R	0.438
VRL12	ATR	NM_001184.3	c.2320dup	p.I774Nfs*3	0.381
VRL12	LATS1	NM_004690.3	c.2449G>T	p.E817*	0.039
VRL12	TEK	NM_000459.4	c.653C>T	p.P218L	0.986
VRL12	TNFRSF14	NM_003820.3	c.349G>A	p.A117T	0.500
VRL12	PIK3CD	NM_005026.3	c.1366A>G	p.T456A	0.500
VRL12	ID3	NM_002167.4	c.313A>G	p.T105A	1.000
VRL12	MYCL	NM_001033082.2	c.1175C>G	p.T392S	1.000
VRL12	FAM46C	NM_017709.3	c.201C>G	p.H67Q	0.500
VRL12	HSD3B1	NM_000862.2	c.1100C>A	p.T367N	1.000
VRL12	NOTCH2	NM_024408.3	c.57C>G	p.C19W	0.500
VRL12	PARP1	NM_001618.3	c.2285T>C	p.V762A	0.500
VRL12	TET1	NM_030625.2	c.485A>G	p.D162G	1.000
VRL12	TET1	NM_030625.2	c.3369A>G	p.I1123M	1.000
VRL12	BMPR1A	NM_004329.2	c.4C>A	p.P2T	1.000
VRL12	BMPR1A	NM_004329.2	c.59G>A	p.R20H	0.500
VRL12	MEN1	NM_000244.3	c.1636A>G	p.T546A	1.000
VRL12	INPPL1	NM_001567.3	c.3248C>G	p.A1083G	0.500
VRL12	ATM	NM_000051.3	c.125A>G	p.H42R	0.500

VRL12	ATM	NM_000051.3	c.5948A>G	p.N1983S	1.000
VRL12	CBL	NM_005188.3	c.1858C>T	p.L620F	0.500
VRL12	KDM5A	NM_001042603.2	c.4420C>A	p.P1474T	0.500
VRL12	KMT2D	NM_003482.3	c.12764G>A	p.G4255D	0.500
VRL12	KMT2D	NM_003482.3	c.10785_10789del	p.Y3595*	0.500
VRL12	KMT2D	NM_003482.3	c.7046C>T	p.P2349L	0.500
VRL12	SH2B3	NM_005475.2	c.784T>C	p.W262R	1.000
VRL12	TBX3	NM_016569.3	c.1685C>T	p.A562V	0.500
VRL12	HNF1A	NM_000545.6	c.79A>C	p.I27L	0.500
VRL12	HNF1A	NM_000545.6	c.1460G>A	p.S487N	0.500
VRL12	HNF1A	NM_000545.6	c.1720A>G	p.S574G	1.000
VRL12	LATS2	NM_014572.2	c.1087G>A	p.G363S	1.000
VRL12	FLT3	NM_004119.2	c.680C>T	p.T227M	0.500
VRL12	FLT3	NM_004119.2	c.20A>G	p.D7G	0.500
VRL12	BRCA2	NM_000059.3	c.865A>C	p.N289H	0.500
VRL12	BRCA2	NM_000059.3	c.1114A>C	p.N372H	0.500
VRL12	BRCA2	NM_000059.3	c.2971A>G	p.N991D	0.500
VRL12	BRCA2	NM_000059.3	c.7397T>C	p.V2466A	1.000
VRL12	IRS2	NM_003749.2	c.3170G>A	p.G1057D	0.500
VRL12	CUL4A	NM_001008895.2	c.1931A>G	p.K644R	0.500
VRL12	BCL2L2	NM_004050.4	c.398A>G	p.Q133R	1.000
VRL12	FOXA1	NM_004496.3	c.247G>A	p.A83T	1.000
VRL12	MLH3	NM_001040108.1	c.2825C>T	p.T942I	1.000
VRL12	MLH3	NM_001040108.1	c.2476A>G	p.N826D	1.000
VRL12	TSHR	NM_000369.2	c.2181G>C	p.E727D	1.000
VRL12	LTK	NM_002344.5	c.125G>A	p.R42Q	0.500
VRL12	MGA	NM_001164273.1	c.4567C>G	p.P1523A	0.500
VRL12	TP53BP1	NM_001141980.1	c.3421A>C	p.K1141Q	0.500
VRL12	TP53BP1	NM_001141980.1	c.1249G>A	p.G417S	0.500
VRL12	TP53BP1	NM_001141980.1	c.1074C>G	p.D358E	0.500
VRL12	BLM	NM_000057.3	c.893C>T	p.T298M	0.500
VRL12	AXIN1	NM_003502.3	c.1750G>A	p.A584T	0.500
VRL12	ZFHX3	NM_006885.3	c.1378G>C	p.E460Q	0.500
VRL12	ZFHX3	NM_006885.3	c.214T>G	p.S72A	0.500
VRL12	FANCA	NM_000135.2	c.2426G>A	p.G809D	1.000
VRL12	FANCA	NM_000135.2	c.1501G>A	p.G501S	0.500
VRL12	FANCA	NM_000135.2	c.796A>G	p.T266A	1.000
VRL12	TP53	NM_000546.5	c.215C>G	p.P72R	1.000
VRL12	AURKB	NM_001284526.1	c.896T>C	p.M299T	1.000
VRL12	ERBB2	NM_004448.3	c.1963A>G	p.I655V	0.500
VRL12	ERBB2	NM_004448.3	c.3508C>G	p.P1170A	0.500
VRL12	BRCA1	NM_007294.3	c.4837A>G	p.S1613G	0.500
VRL12	BRCA1	NM_007294.3	c.3548A>G	p.K1183R	0.500
VRL12	BRCA1	NM_007294.3	c.3113A>G	p.E1038G	0.500
VRL12	BRCA1	NM_007294.3	c.2612C>T	p.P871L	0.500
VRL12	RNF43	NM_017763.5	c.662G>A	p.R221Q	1.000
VRL12	RNF43	NM_017763.5	c.350G>A	p.R117H	1.000
VRL12	BRIP1	NM_032043.2	c.2755T>C	p.S919P	0.500
VRL12	AXIN2	NM_004655.3	c.148C>T	p.P50S	0.500
VRL12	BCL2	NM_000633.2	c.127G>A	p.A43T	0.500
VRL12	TCF3	NM_001136139.2	c.1475C>T	p.A492V	0.500
VRL12	DOT1L	NM_032482.2	c.4156G>A	p.G1386S	0.500
VRL12	DOT1L	NM_032482.2	c.4252G>C	p.V1418L	0.500
VRL12	PTPRS	NM_002850.3	c.4369T>C	p.C1457R	1.000
VRL12	DNMT1	NM_001130823.2	c.979A>G	p.I327V	0.500
VRL12	DNMT1	NM_001130823.2	c.206G>A	p.R69H	0.500

VRL12	CALR	NM_004343.3	c.1142A>C	p.E381A	0.500
VRL12	NOTCH3	NM_000435.2	c.6668C>T	p.A2223V	0.500
VRL12	PIK3R2	NM_005027.3	c.700A>C	p.S234R	1.000
VRL12	PIK3R2	NM_005027.3	c.937T>C	p.S313P	1.000
VRL12	KMT2B	NM_014727.2	c.3059dup	p.R1021Pfs*14	1.000
VRL12	KMT2B	NM_014727.2	c.5591G>A	p.R1864Q	0.500
VRL12	KMT2B	NM_014727.2	c.7091A>G	p.D2364G	0.500
VRL12	AXL	NM_021913.4	c.796A>G	p.N266D	1.000
VRL12	CD79A	NM_001783.3	c.572T>C	p.L191P	0.500
VRL12	CD3EAP	NM_001297590.1	c.1516C>A	p.Q506K	1.000
VRL12	ALK	NM_004304.4	c.4587C>G	p.D1529E	1.000
VRL12	ALK	NM_004304.4	c.4472A>G	p.K1491R	1.000
VRL12	ALK	NM_004304.4	c.4381A>G	p.I1461V	1.000
VRL12	EPCAM	NM_002354.2	c.344T>C	p.M115T	0.500
VRL12	MSH2	NM_000251.2	c.1168C>T	p.L390F	0.500
VRL12	MERTK	NM_006343.2	c.1397G>A	p.R466K	0.500
VRL12	MERTK	NM_006343.2	c.1552A>G	p.I518V	0.500
VRL12	LRP1B	NM_018557.2	c.143A>G	p.Q48R	0.500
VRL12	CTLA4	NM_005214.4	c.49A>G	p.T17A	1.000
VRL12	IRS1	NM_005544.2	c.3235C>T	p.P1079S	0.500
VRL12	PDCD1	NM_005018.2	c.644C>T	p.A215V	1.000
VRL12	ASXL1	NM_015338.5	c.2444T>C	p.L815P	1.000
VRL12	NCOA3	NM_181659.2	c.3789_3791del	p.Q1276del	0.500
VRL12	ZNF217	NM_006526.2	c.1643C>T	p.T548I	1.000
VRL12	AURKA	NM_003600.3	c.169A>G	p.I57V	1.000
VRL12	AURKA	NM_003600.3	c.91T>A	p.F31I	1.000
VRL12	RTEL1	NM_001283009.1	c.3126A>C	p.Q1042H	1.000
VRL12	TMPRSS2	NM_005656.3	c.478G>A	p.V160M	0.500
VRL12	EP300	NM_001429.3	c.2989A>G	p.I997V	0.500
VRL12	PNPLA3	NM_025225.2	c.444C>G	p.I148M	0.500
VRL12	SETD2	NM_014159.6	c.5885C>T	p.P1962L	0.500
VRL12	ATR	NM_001184.3	c.7274G>A	p.R2425Q	1.000
VRL12	ATR	NM_001184.3	c.632T>C	p.M211T	1.000
VRL12	KDR	NM_002253.2	c.1416A>T	p.Q472H	0.500
VRL12	KDR	NM_002253.2	c.889G>A	p.V297I	0.500
VRL12	ABRAXAS1	NM_139076.2	c.1042G>A	p.A348T	1.000
VRL12	TET2	NM_001127208.2	c.86C>G	p.P29R	0.500
VRL12	TET2	NM_001127208.2	c.5162T>G	p.L1721W	0.500
VRL12	FAT1	NM_005245.3	c.12177G>C	p.K4059N	1.000
VRL12	FAT1	NM_005245.3	c.10660T>G	p.S3554A	0.500
VRL12	FAT1	NM_005245.3	c.10001T>C	p.V3334A	0.500
VRL12	FAT1	NM_005245.3	c.8798A>C	p.Q2933P	0.500
VRL12	FAT1	NM_005245.3	c.8494A>G	p.R2832G	0.500
VRL12	FAT1	NM_005245.3	c.4985A>G	p.N1662S	0.500
VRL12	FAT1	NM_005245.3	c.4892C>T	p.A1631V	0.500
VRL12	FAT1	NM_005245.3	c.3818A>G	p.H1273R	0.500
VRL12	IL7R	NM_002185.3	c.197T>C	p.I66T	0.500
VRL12	IL7R	NM_002185.3	c.412G>A	p.V138I	1.000
VRL12	MAP3K1	NM_005921.1	c.2716G>A	p.V906I	0.500
VRL12	MAP3K1	NM_005921.1	c.2845_2847del	p.T949del	0.500
VRL12	MSH3	NM_002439.4	c.181_189dup	p.A61_P63dup	0.500
VRL12	MSH3	NM_002439.4	c.2846A>G	p.Q949R	1.000
VRL12	APC	NM_000038.5	c.5465T>A	p.V1822D	1.000
VRL12	CSF1R	NM_005211.3	c.1085A>G	p.H362R	0.500
VRL12	FGFR4	NM_002011.4	c.28G>A	p.V10I	0.500
VRL12	FGFR4	NM_002011.4	c.407C>T	p.P136L	1.000

VRL12	FGFR4	NM_002011.4	c.535A>G	p.T179A	0.500
VRL12	FGFR4	NM_002011.4	c.1162G>A	p.G388R	0.500
VRL12	NSD1	NM_022455.4	c.1840G>T	p.V614L	0.500
VRL12	NSD1	NM_022455.4	c.2176T>C	p.S726P	0.500
VRL12	FLT4	NM_182925.4	c.2670C>G	p.H890Q	0.500
VRL12	HIST1H1C	NM_005319.3	c.53C>T	p.A18V	1.000
VRL12	NOTCH4	NM_004557.3	c.349A>C	p.K117Q	1.000
VRL12	TAP2	NM_000544.3	c.1135G>A	p.V379I	1.000
VRL12	CDKN1A	NM_001291549.1	c.83A>G	p.D28G	1.000
VRL12	CDKN1A	NM_001291549.1	c.195C>A	p.S65R	0.500
VRL12	CCND3	NM_001760.4	c.775T>G	p.S259A	0.500
VRL12	EPHA7	NM_004440.3	c.832C>T	p.P278S	1.000
VRL12	EPHA7	NM_004440.3	c.412A>G	p.I138V	1.000
VRL12	ROS1	NM_002944.2	c.6686C>G	p.S2229C	1.000
VRL12	ROS1	NM_002944.2	c.6682A>C	p.K2228Q	1.000
VRL12	ROS1	NM_002944.2	c.6637G>A	p.D2213N	1.000
VRL12	ROS1	NM_002944.2	c.3857G>A	p.R1286H	1.000
VRL12	IFNGR1	NM_000416.2	c.665A>G	p.H222R	1.000
VRL12	PMS2	NM_000535.6	c.1621A>G	p.K541E	1.000
VRL12	PMS2	NM_000535.6	c.1454C>A	p.T485K	0.500
VRL12	PMS2	NM_000535.6	c.1408C>T	p.P470S	0.500
VRL12	ETV1	NM_004956.4	c.298A>G	p.S100G	0.500
VRL12	EGFR	NM_005228.3	c.1562G>A	p.R521K	1.000
VRL12	KMT2C	NM_170606.2	c.10979C>T	p.S3660L	0.500
VRL12	KMT2C	NM_170606.2	c.2963G>T	p.C988F	0.500
VRL12	KMT2C	NM_170606.2	c.2959T>C	p.Y987H	0.500
VRL12	KMT2C	NM_170606.2	c.2512G>A	p.G838S	0.500
VRL12	KMT2C	NM_170606.2	c.2447dup	p.Y816*	0.500
VRL12	PREX2	NM_024870.3	c.4463C>T	p.S1488L	1.000
VRL12	PRDM14	NM_024504.3	c.730A>G	p.K244E	0.500
VRL12	RECQL4	NM_004260.3	c.274T>C	p.S92P	1.000
VRL12	PDCD1LG2	NM_025239.3	c.686T>C	p.F229S	1.000
VRL12	PTCH1	NM_000264.3	c.3944C>T	p.P1315L	0.500
VRL12	EGFL7	NM_016215.4	c.457G>A	p.V153I	0.500
VRL12	AR	NM_000044.3	c.231_239dup	p.Q78_Q80dup	0.500
VRL12	ATRX	NM_000489.4	c.2785C>G	p.Q929E	1.000
VRL12	BCORL1	NM_001184772.2	c.331T>C	p.F111L	1.000
VRL12	CCNQ	NM_152274.4	c.16dup	p.G7Rfs*51	1.000
VRL13	APC	NM_000038.5	c.5465T>A	p.V1822D	1.000
VRL13	ATM	NM_000051.3	c.5948A>G	p.N1983S	1.000
VRL13	AXIN2	NM_004655.3	c.148C>T	p.P50S	1.000
VRL13	BARD1	NM_000465.3	c.70C>T	p.P24S	0.996
VRL13	BARD1	NM_000465.3	c.1134G>C	p.R378S	1.000
VRL13	BARD1	NM_000465.3	c.1519G>A	p.V507M	0.994
VRL13	BCL10	NM_003921.4	c.13G>T	p.A5S	0.382
VRL13	BLM	NM_000057.3	c.893C>T	p.T298M	0.537
VRL13	BMPR1A	NM_004329.2	c.4C>A	p.P2T	0.391
VRL13	BRCA1	NM_007294.3	c.3113A>G	p.E1038G	0.500
VRL13	BRCA1	NM_007294.3	c.2612C>T	p.P871L	0.454
VRL13	BRCA1	NM_007294.3	c.4837A>G	p.S1613G	0.528
VRL13	BRCA1	NM_007294.3	c.3548A>G	p.K1183R	0.513
VRL13	BRCA2	NM_000059.3	c.1114A>C	p.N372H	0.500
VRL13	BRCA2	NM_000059.3	c.8187G>T	p.K2729N	0.485
VRL13	BRCA2	NM_000059.3	c.10234A>G	p.I3412V	0.498
VRL13	BRCA2	NM_000059.3	c.7397T>C	p.V2466A	1.000
VRL13	BRIP1	NM_032043.2	c.2755T>C	p.S919P	0.472

VRL13	CHEK1	NM_001114121.2	c.1411A>G	p.I471V	1.000
VRL13	EPCAM	NM_002354.2	c.344T>C	p.M115T	1.000
VRL13	HNF1A	NM_000545.6	c.1720A>G	p.S574G	1.000
VRL13	HNF1A	NM_000545.6	c.1460G>A	p.S487N	1.000
VRL13	HNF1A	NM_000545.6	c.79A>C	p.I27L	0.510
VRL13	MSH6	NM_000179.2	c.116G>A	p.G39E	1.000
VRL13	PMS2	NM_000535.6	c.2570G>C	p.G857A	0.533
VRL13	PMS2	NM_000535.6	c.1408C>T	p.P470S	1.000
VRL13	PMS2	NM_000535.6	c.1621A>G	p.K541E	1.000
VRL13	STK11	NM_000455.4	c.1062C>G	p.F354L	0.434
VRL13	TP53	NM_000546.5	c.215C>G	p.P72R	0.452
VRL14	MYD88	NM_002468.5	c.794T>C	p.L265P	0.452
VRL14	APC	NM_000038.5	c.5465T>A	p.V1822D	0.996
VRL14	ATM	NM_000051.3	c.5948A>G	p.N1983S	1.000
VRL14	ATR	NM_001184.3	c.632T>C	p.M211T	0.473
VRL14	BARD1	NM_000465.3	c.1134G>C	p.R378S	1.000
VRL14	BARD1	NM_000465.3	c.1519G>A	p.V507M	1.000
VRL14	BARD1	NM_000465.3	c.70C>T	p.P24S	0.473
VRL14	BCL10	NM_003921.4	c.13G>T	p.A5S	0.397
VRL14	BMPR1A	NM_004329.2	c.4C>A	p.P2T	1.000
VRL14	BRCA2	NM_000059.3	c.7397T>C	p.V2466A	1.000
VRL14	BRCA2	NM_000059.3	c.1114A>C	p.N372H	1.000
VRL14	BRIP1	NM_032043.2	c.2755T>C	p.S919P	1.000
VRL14	CHEK1	NM_001114121.2	c.1411A>G	p.I471V	1.000
VRL14	HNF1A	NM_000545.6	c.79A>C	p.I27L	0.997
VRL14	HNF1A	NM_000545.6	c.1720A>G	p.S574G	0.998
VRL14	HNF1A	NM_000545.6	c.1460G>A	p.S487N	1.000
VRL14	NBN	NM_002485.4	c.553G>C	p.E185Q	1.000
VRL14	PALB2	NM_024675.3	c.398G>C	p.S133T	0.222
VRL14	PALB2	NM_024675.3	c.1676A>G	p.Q559R	0.441
VRL14	PMS2	NM_000535.6	c.1454C>A	p.T485K	0.469
VRL14	PMS2	NM_000535.6	c.1621A>G	p.K541E	1.000
VRL14	PMS2	NM_000535.6	c.59G>A	p.R20Q	0.476
VRL14	PMS2	NM_000535.6	c.2570G>C	p.G857A	0.344
VRL14	POLE	NM_006231.3	c.755C>T	p.A252V	0.502
VRL14	TP53	NM_000546.5	c.215C>G	p.P72R	0.505
VRL15	MYD88	NM_002468.5	c.794T>C	p.L265P	0.476
VRL15	APC	NM_000038.5	c.8383G>A	p.A2795T	0.505
VRL15	ATM	NM_000051.3	c.5948A>G	p.N1983S	1.000
VRL15	ATR	NM_001184.3	c.2046G>C	p.Q682H	0.474
VRL15	AXIN2	NM_004655.3	c.148C>T	p.P50S	0.492
VRL15	BLM	NM_000057.3	c.893C>T	p.T298M	0.498
VRL15	BMPR1A	NM_004329.2	c.4C>A	p.P2T	0.447
VRL15	BRCA2	NM_000059.3	c.7397T>C	p.V2466A	0.999
VRL15	BRCA2	NM_000059.3	c.1114A>C	p.N372H	0.512
VRL15	BRIP1	NM_032043.2	c.2755T>C	p.S919P	1.000
VRL15	CHEK1	NM_001114121.2	c.1411A>G	p.I471V	1.000
VRL15	EPCAM	NM_002354.2	c.344T>C	p.M115T	0.460
VRL15	HNF1A	NM_000545.6	c.1460G>A	p.S487N	0.999
VRL15	HNF1A	NM_000545.6	c.1720A>G	p.S574G	1.000
VRL15	HNF1A	NM_000545.6	c.79A>C	p.I27L	1.000
VRL15	MSH2	NM_000251.2	c.1886A>G	p.Q629R	0.489
VRL15	NBN	NM_002485.4	c.553G>C	p.E185Q	0.444
VRL15	PMS2	NM_000535.6	c.1408C>T	p.P470S	1.000
VRL15	PMS2	NM_000535.6	c.1621A>G	p.K541E	1.000
VRL15	PMS2	NM_000535.6	c.2570G>C	p.G857A	0.499

VRL15	PMS2	NM_000535.6	c.1532C>T	p.T511M	0.496
VRL15	POLD1	NM_002691.3	c.356G>A	p.R119H	0.499
VRL15	TP53	NM_000546.5	c.215C>G	p.P72R	0.999
VRL16	APC	NM_000038	c.5465T>A	p.V1822D	1.000
VRL16	ATM	NM_000051	c.5948A>G	p.N1983S	1.000
VRL16	ATR	NM_001184	c.632T>C	p.M211T	0.511
VRL16	AXIN2	NM_004655	c.148C>T	p.P50S	0.447
VRL16	BARD1	NM_000465	c.1519G>A	p.V507M	0.420
VRL16	BARD1	NM_000465	c.70C>T	p.P24S	0.443
VRL16	BARD1	NM_000465	c.1134G>C	p.R378S	0.449
VRL16	BLM	NM_000057	c.893C>T	p.T298M	0.414
VRL16	BMPR1A	NM_004329	c.4C>A	p.P2T	0.333
VRL16	BRCA1	NM_007294	c.3548A>G	p.K1183R	0.519
VRL16	BRCA1	NM_007294	c.2612C>T	p.P871L	0.438
VRL16	BRCA1	NM_007294	c.3113A>G	p.E1038G	0.438
VRL16	BRCA1	NM_007294	c.4837A>G	p.S1613G	0.551
VRL16	BRCA2	NM_000059	c.7397T>C	p.V2466A	0.993
VRL16	BRIP1	NM_032043	c.2755T>C	p.S919P	1.000
VRL16	BRIP1	NM_032043	c.2440C>T	p.R814C	0.364
VRL16	CHEK1	NM_001114121	c.1411A>G	p.I471V	1.000
VRL16	EPCAM	NM_002354	c.344T>C	p.M115T	1.000
VRL16	FLT3	NM_004119	c.1073A>T	p.D358V	0.492
VRL16	HNF1A	NM_000545	c.1460G>A	p.S487N	0.450
VRL16	HNF1A	NM_000545	c.1720A>G	p.S574G	1.000
VRL16	HNF1A	NM_000545	c.79A>C	p.I27L	0.460
VRL16	KIT	NM_000222	c.1621A>C	p.M541L	0.443
VRL16	MSH2	NM_000251	c.1168C>T	p.L390F	0.477
VRL16	MUTYH	NM_012222	c.1005G>C	p.Q335H	1.000
VRL16	PALB2	NM_024675	c.398G>C	p.S133T	0.253
VRL16	PMS2	NM_000535	c.1408C>T	p.P470S	0.464
VRL16	PMS2	NM_000535	c.1621A>G	p.K541E	1.000
VRL16	POLE	NM_006231	c.755C>T	p.A252V	0.446
VRL16	RAD51D	NM_002878	c.494G>A	p.R165Q	0.379
VRL16	TP53	NM_000546	c.215C>G	p.P72R	0.451
VRL16	MYD88	NM_002468.5	c.794T>C	p.L265P	0.293
VRL16	NF2	NM_000268.3	c.589G>A	p.G197S	0.028
VRL16	CD79B	NM_000626.3	c.586T>C	p.Y196H	0.252
VRL16	IGF1R	NM_000875.4	c.1943G>T	p.W648L	0.029
VRL16	KDM5A	NM_001042603.2	c.3989C>T	p.S1330F	0.024
VRL16	AURKB	NM_001313954.1	c.76G>T	p.G26W	0.030
VRL16	EP300	NM_001429.3	c.4410G>T	p.M1470I	0.037
VRL16	BTG1	NM_001731.2	c.168G>A	p.W56*	0.306
VRL16	H3F3A	NM_002107.4	c.386G>A	p.R129H	0.020
VRL16	IRF4	NM_002460.3	c.161G>A	p.W54*	0.262
VRL16	RECQL	NM_002907.3	c.170C>A	p.A57E	0.024
VRL16	NKX2-1	NM_003317.3	c.152C>T	p.A51V	0.273
VRL16	TP53BP1	NM_005657.3	c.3841C>A	p.Q1281K	0.031
VRL16	SMAD2	NM_005901.5	c.463C>A	p.L155I	0.028
VRL16	KMT2A	NM_005933.3	c.5882C>A	p.A1961D	0.026
VRL16	SF3B1	NM_012433.3	c.1816C>A	p.L606M	0.034
VRL16	SUZ12	NM_015355.3	c.1477G>T	p.E493*	0.036
VRL16	ERAP1	NM_016442.4	c.2336C>A	p.T779K	0.020
VRL16	ASXL2	NM_018263	c.341G>A	p.S114N	0.021
VRL16	LRP1B	NM_018557.2	c.10960G>A	p.G3654R	0.308
VRL16	ABCB9	NM_019624.3	c.1150G>T	p.E384*	0.028
VRL16	KDM6A	NM_021140.3	c.1354G>A	p.G452R	0.034

VRL16	BCORL1	NM_021946.4	c.683C>A	p.P228H	0.011
VRL16	PIK3R1	NM_181523.2	c.689C>A	p.P230H	0.027
VRL17	MYD88	NM_002468.5	c.794T>C	p.L265P	0.994
VRL17	ALK	NM_004304.4	c.487G>T	p.V163L	0.502
VRL17	ATM	NM_000051.3	c.5948A>G	p.N1983S	1.000
VRL17	ATR	NM_001184.3	c.632T>C	p.M211T	0.528
VRL17	AXIN2	NM_004655.3	c.148C>T	p.P50S	0.455
VRL17	BMPR1A	NM_004329.2	c.4C>A	p.P2T	0.352
VRL17	BRCA2	NM_000059.3	c.7397T>C	p.V2466A	1.000
VRL17	BRCA2	NM_000059.3	c.1114A>C	p.N372H	1.000
VRL17	BRIP1	NM_032043.2	c.2755T>C	p.S919P	1.000
VRL17	CHEK1	NM_001114121.2	c.334delC	p.H112fs	0.447
VRL17	CHEK1	NM_001114121.2	c.1411A>G	p.I471V	1.000
VRL17	EPCAM	NM_002354.2	c.344T>C	p.M115T	1.000
VRL17	HNF1A	NM_000545.6	c.1720A>G	p.S574G	0.998
VRL17	MLH1	NM_000249.3	c.1151T>A	p.V384D	0.470
VRL17	PALB2	NM_024675.3	c.1676A>G	p.Q559R	0.470
VRL17	PALB2	NM_024675.3	c.398G>C	p.S133T	0.217
VRL17	PMS2	NM_000535.6	c.1621A>G	p.K541E	1.000
VRL17	PMS2	NM_000535.6	c.1408C>T	p.P470S	0.501
VRL17	PMS2	NM_000535.6	c.1454C>A	p.T485K	0.479
VRL17	TP53	NM_000546.5	c.215C>G	p.P72R	1.000
VRL18	APC	NM_000038.5	c.5465T>A	p.V1822D	0.490
VRL18	ATM	NM_000051.3	c.5948A>G	p.N1983S	1.000
VRL18	ATR	NM_001184.3	c.325C>T	p.R109W	0.468
VRL18	ATR	NM_001184.3	c.7274G>A	p.R2425Q	0.464
VRL18	ATR	NM_001184.3	c.632T>C	p.M211T	1.000
VRL18	AXIN2	NM_004655.3	c.148C>T	p.P50S	0.452
VRL18	BARD1	NM_000465.3	c.1134G>C	p.R378S	0.434
VRL18	BARD1	NM_000465.3	c.70C>T	p.P24S	0.437
VRL18	BARD1	NM_000465.3	c.1519G>A	p.V507M	0.420
VRL18	BMPR1A	NM_004329.2	c.4C>A	p.P2T	0.279
VRL18	BRCA2	NM_000059.3	c.7397T>C	p.V2466A	1.000
VRL18	BRIP1	NM_032043.2	c.430G>A	p.A144T	0.467
VRL18	BRIP1	NM_032043.2	c.2755T>C	p.S919P	0.474
VRL18	CHEK1	NM_001114121.2	c.1411A>G	p.I471V	1.000
VRL18	EPCAM	NM_002354.2	c.344T>C	p.M115T	1.000
VRL18	HNF1A	NM_000545.6	c.1460G>A	p.S487N	1.000
VRL18	HNF1A	NM_000545.6	c.79A>C	p.I27L	0.476
VRL18	HNF1A	NM_000545.6	c.1720A>G	p.S574G	1.000
VRL18	MSH6	NM_000179.2	c.116G>A	p.G39E	0.437
VRL18	MUTYH	NM_012222.2	c.1005G>C	p.Q335H	0.456
VRL18	NBN	NM_002485.4	c.553G>C	p.E185Q	0.428
VRL18	PALB2	NM_024675.3	c.1676A>G	p.Q559R	0.540
VRL18	PALB2	NM_024675.3	c.398G>C	p.S133T	0.246
VRL18	PMS2	NM_000535.6	c.1621A>G	p.K541E	1.000
VRL18	PMS2	NM_000535.6	c.59G>A	p.R20Q	0.511
VRL18	PMS2	NM_000535.6	c.1408C>T	p.P470S	0.504
VRL18	TP53	NM_000546.5	c.215C>G	p.P72R	0.425
VRL19	MYD88	NM_002468.5	c.794T>C	p.L265P	0.461
VRL19	ALK	NM_004304	c.3035C>T	p.T1012M	0.480
VRL19	APC	NM_000038	c.5465T>A	p.V1822D	0.523
VRL19	ATM	NM_000051	c.5948A>G	p.N1983S	1.000
VRL19	ATR	NM_001184	c.632T>C	p.M211T	0.446
VRL19	AXIN2	NM_004655	c.1807G>C	p.A603P	0.496
VRL19	BCL10	NM_003921	c.13G>T	p.A5S	0.425

VRL19	BMPR1A	NM_004329	c.4C>A	p.P2T	1.000
VRL19	BRCA2	NM_000059	c.1114A>C	p.N372H	0.532
VRL19	BRCA2	NM_000059	c.7397T>C	p.V2466A	1.000
VRL19	BRIP1	NM_032043	c.2755T>C	p.S919P	1.000
VRL19	CHEK1	NM_001114121	c.1411A>G	p.I471V	1.000
VRL19	EPCAM	NM_002354	c.344T>C	p.M115T	1.000
VRL19	HNF1A	NM_000545	c.1460G>A	p.S487N	0.466
VRL19	HNF1A	NM_000545	c.1720A>G	p.S574G	1.000
VRL19	HNF1A	NM_000545	c.79A>C	p.I27L	0.448
VRL19	KIT	NM_000222	c.1621A>C	p.M541L	0.448
VRL19	MEN1	NM_130799	c.1508G>A	p.G503D	0.486
VRL19	MSH6	NM_000179	c.116G>A	p.G39E	0.467
VRL19	PALB2	NM_024675	c.1676A>G	p.Q559R	0.420
VRL19	PALB2	NM_024675	c.398G>C	p.S133T	0.212
VRL19	PMS2	NM_000535	c.1621A>G	p.K541E	0.997
VRL19	PMS2	NM_000535	c.379G>A	p.A127T	0.466
VRL19	PMS2	NM_000535	c.2570G>C	p.G857A	1.000
VRL19	PMS2	NM_000535	c.1408C>T	p.P470S	1.000
VRL19	POLE	NM_006231	c.755C>T	p.A252V	0.515
VRL19	TEK	NM_000459	c.443T>C	p.I148T	0.441
VRL20	KRAS	NM_033360.3	c.40G>A	p.V14I	0.439
VRL20	MYD88	NM_002468.5	c.794T>C	p.L265P	0.449
VRL20	FGFR2	NM_000141.4	c.1849T>G	p.L617V	0.433
VRL20	APC	NM_000038.5	c.5465T>A	p.V1822D	0.995
VRL20	ATM	NM_000051.3	c.5948A>G	p.N1983S	1.000
VRL20	ATR	NM_001184.3	c.632T>C	p.M211T	1.000
VRL20	BLM	NM_000057.3	c.893C>T	p.T298M	0.487
VRL20	BMPR1A	NM_004329.2	c.4C>A	p.P2T	1.000
VRL20	BRCA1	NM_007294.3	c.3113A>G	p.E1038G	1.000
VRL20	BRCA1	NM_007294.3	c.3548A>G	p.K1183R	1.000
VRL20	BRCA1	NM_007294.3	c.4837A>G	p.S1613G	1.000
VRL20	BRCA1	NM_007294.3	c.2612C>T	p.P871L	1.000
VRL20	BRCA2	NM_000059.3	c.7397T>C	p.V2466A	1.000
VRL20	BRCA2	NM_000059.3	c.1114A>C	p.N372H	0.468
VRL20	BRIP1	NM_032043.2	c.2755T>C	p.S919P	1.000
VRL20	CHEK1	NM_001114121.2	c.1411A>G	p.I471V	1.000
VRL20	EP300	NM_001429.3	c.6481A>G	p.M2161V	0.496
VRL20	EPCAM	NM_002354.2	c.344T>C	p.M115T	0.571
VRL20	HNF1A	NM_000545.6	c.1720A>G	p.S574G	1.000
VRL20	MUTYH	NM_012222.2	c.1005G>C	p.Q335H	0.441
VRL20	NBN	NM_002485.4	c.553G>C	p.E185Q	0.429
VRL20	PALB2	NM_024675.3	c.398G>C	p.S133T	0.230
VRL20	PMS2	NM_000535.6	c.1408C>T	p.P470S	0.491
VRL20	PMS2	NM_000535.6	c.2570G>C	p.G857A	1.000
VRL20	PMS2	NM_000535.6	c.1621A>G	p.K541E	1.000
VRL20	POLE	NM_006231.3	c.755C>T	p.A252V	0.434
VRL20	TP53	NM_000546.5	c.215C>G	p.P72R	0.459
VRL21	MYD88	NM_002468.5	c.794T>C	p.L265P	0.492
VRL21	AR	NM_000044.4	c.1163A>G	p.K388R	0.254
VRL21	BTK	NM_000061.2	c.1939C>A	p.L647I	0.288
VRL21	KMT5A	NM_001324506.1	c.580T>C	p.C194R	0.012
VRL21	ETV6	NM_001987.4	c.1164C>A	p.N388K	0.296
VRL21	TGFBR2	NM_003242.5	c.374A>G	p.E125G	0.010
VRL21	EZH2	NM_004456.4	c.1544A>G	p.K515R	0.006
VRL21	IRS1	NM_005544.2	c.109G>A	p.E37K	0.012
VRL21	PIK3CB	NM_006219.2	c.1810dupC	p.R604fs	0.009

VRL21	RANBP2	NM_006267.4	c.1114A>G	p.I372V	0.017
VRL21	LATS2	NM_014572.2	c.1031dupC	p.Q345fs	0.010
VRL21	TBX3	NM_016569.3	c.966G>T	p.M322I	0.349
VRL21	AXL	NM_021913.4	c.874delC	p.H292fs	0.006
VRL21	TAF1	NM_138923.3	c.2942C>T	p.P981L	0.221
VRL21	AMER1	NM_152424.4	c.62G>A	p.R21H	0.206
VRL21	APC	NM_000038	c.5465T>A	p.V1822D	0.517
VRL21	ATM	NM_000051	c.5948A>G	p.N1983S	0.999
VRL21	ATR	NM_001184	c.632T>C	p.M211T	0.506
VRL21	AXIN2	NM_004655	c.148C>T	p.P50S	0.450
VRL21	BARD1	NM_000465	c.1134G>C	p.R378S	0.495
VRL21	BARD1	NM_000465	c.1519G>A	p.V507M	0.455
VRL21	BARD1	NM_000465	c.70C>T	p.P24S	0.476
VRL21	BMPR1A	NM_004329	c.4C>A	p.P2T	1.000
VRL21	BRCA2	NM_000059	c.1114A>C	p.N372H	0.665
VRL21	BRCA2	NM_000059	c.10234A>G	p.I3412V	0.316
VRL21	BRCA2	NM_000059	c.7397T>C	p.V2466A	1.000
VRL21	BRIP1	NM_032043	c.2755T>C	p.S919P	0.485
VRL21	CHEK1	NM_001114121	c.1411A>G	p.I471V	1.000
VRL21	EPCAM	NM_002354	c.344T>C	p.M115T	1.000
VRL21	HNF1A	NM_000545	c.1720A>G	p.S574G	1.000
VRL21	HNF1A	NM_000545	c.79A>C	p.I27L	0.662
VRL21	NBN	NM_002485	c.553G>C	p.E185Q	0.559
VRL21	PMS2	NM_000535	c.1621A>G	p.K541E	1.000
VRL21	PMS2	NM_000535	c.1408C>T	p.P470S	0.432
VRL21	PMS2	NM_000535	c.2570G>C	p.G857A	0.706
VRL21	PMS2	NM_000535	c.1454C>A	p.T485K	0.531
VRL21	STK11	NM_000455	c.1062C>G	p.F354L	0.505
VRL21	TSC2	NM_000548	c.2032G>A	p.A678T	0.515
VRL22	RUNX1	NM_001001890.2	c.73A>C	p.M25L	0.012
VRL22	HIST2H3D	NM_001123375.2	c.260G>A	p.S87N	0.270
VRL22	ETV6	NM_001987.4	c.1138T>G	p.W380G	0.309
VRL22	TBX3	NM_016569.3	c.1577C>T	p.A526V	0.321
VRL22	ARID1B	NM_017519.2	c.811G>T	p.A271S	0.011
VRL22	MCL1	NM_021960.4	c.464G>A	p.S155N	0.288
VRL22	WT1	NM_024426.5	c.797delC	p.P266fs	0.007
VRL22	APC	NM_000038	c.5465T>A	p.V1822D	1.000
VRL22	ATM	NM_000051	c.5948A>G	p.N1983S	0.999
VRL22	ATR	NM_001184	c.632T>C	p.M211T	0.497
VRL22	BMPR1A	NM_004329	c.4C>A	p.P2T	1.000
VRL22	BRCA2	NM_000059	c.7397T>C	p.V2466A	1.000
VRL22	BRCA2	NM_000059	c.1114A>C	p.N372H	0.529
VRL22	BRCA2	NM_000059	c.8474C>A	p.A2825E	0.485
VRL22	BRIP1	NM_032043	c.2755T>C	p.S919P	1.000
VRL22	CHEK1	NM_001114121	c.1411A>G	p.I471V	1.000
VRL22	EPCAM	NM_002354	c.515C>T	p.T172M	0.431
VRL22	EPCAM	NM_002354	c.344T>C	p.M115T	1.000
VRL22	HNF1A	NM_000545	c.1720A>G	p.S574G	1.000
VRL22	MLH1	NM_000249	c.655A>G	p.I219V	0.486
VRL22	PMS2	NM_000535	c.1621A>G	p.K541E	1.000
VRL22	PMS2	NM_000535	c.1408C>T	p.P470S	0.314
VRL22	PMS2	NM_000535	c.2570G>C	p.G857A	0.517
VRL22	POLD1	NM_002691	c.356G>A	p.R119H	0.490
VRL22	TP53	NM_000546	c.215C>G	p.P72R	1.000
VRL23	MYD88	NM_002468.5	c.794T>C	p.L265P	0.079
VRL23	SYK	NM_001135052.3	c.1553C>T	p.P518L	0.061

VRL23	PIM1	NM_001243186	c.1024G>A	p.G342S	0.040
VRL23	IRS2	NM_003749.2	c.736C>T	p.R246C	0.019
VRL23	CTCF	NM_006565.3	c.1802A>G	p.K601R	0.533
VRL23	ABL2	NM_007314.3	c.667A>G	p.N223D	0.580
VRL23	NSD1	NM_022455.4	c.6991G>A	p.D2331N	0.500
VRL23	CARD11	NM_032415.5	c.1010G>A	p.R337Q	0.059
VRL23	CCND1	NM_053056.2	c.205G>A	p.E69K	0.426
VRL23	APC	NM_000038	c.5465T>A	p.V1822D	1.000
VRL23	ATM	NM_000051	c.5948A>G	p.N1983S	1.000
VRL23	BMPR1A	NM_004329	c.4C>A	p.P2T	1.000
VRL23	BRCA1	NM_007294	c.4045A>C	p.T1349P	0.546
VRL23	BRCA1	NM_007294	c.3113A>G	p.E1038G	1.000
VRL23	BRCA1	NM_007294	c.2612C>T	p.P871L	1.000
VRL23	BRCA1	NM_007294	c.4837A>G	p.S1613G	1.000
VRL23	BRCA1	NM_007294	c.3548A>G	p.K1183R	0.989
VRL23	BRCA2	NM_000059	c.7397T>C	p.V2466A	1.000
VRL23	BRCA2	NM_000059	c.1114A>C	p.N372H	1.000
VRL23	BRIP1	NM_032043	c.2755T>C	p.S919P	0.433
VRL23	CHEK1	NM_001114121	c.1411A>G	p.I471V	1.000
VRL23	EPCAM	NM_002354	c.344T>C	p.M115T	1.000
VRL23	HNF1A	NM_000545	c.79A>C	p.I27L	1.000
VRL23	HNF1A	NM_000545	c.1460G>A	p.S487N	0.992
VRL23	HNF1A	NM_000545	c.1720A>G	p.S574G	1.000
VRL23	KIT	NM_000222	c.1621A>C	p.M541L	0.587
VRL23	MSH6	NM_000179	c.116G>A	p.G39E	0.455
VRL23	NBN	NM_002485	c.553G>C	p.E185Q	0.415
VRL23	NSD1	NM_022455	c.6991G>A	p.D2331N	0.500
VRL23	PMS2	NM_000535	c.1621A>G	p.K541E	1.000
VRL23	PMS2	NM_000535	c.1454C>A	p.T485K	0.487
VRL23	PMS2	NM_000535	c.1408C>T	p.P470S	0.505
VRL23	TP53	NM_000546	c.215C>G	p.P72R	1.000
VRL23	TSC1	NM_000368	c.965T>C	p.M322T	0.570
Ctrl1	ITGB3	NM_000212.2	c.923C>G	p.S308C	0.475
Ctrl1	ESR1	NM_001122742.1	c.806G>A	p.R269H	0.477
Ctrl1	PDPK1	NM_002613.4	c.1353T>G	p.F451L	0.460
Ctrl1	PIK3C2G	NM_004570.5	c.4180G>A	p.E1394K	0.010
Ctrl1	IRS1	NM_005544.2	c.109G>A	p.E37K	0.007
Ctrl1	DOT1L	NM_032482.2	c.4607G>A	p.G1536D	0.490
Ctrl1	APC	NM_000038	c.5465T>A	p.V1822D	1.000
Ctrl1	ATM	NM_000051	c.4258C>T	p.L1420F	0.479
Ctrl1	ATM	NM_000051	c.5948A>G	p.N1983S	1.000
Ctrl1	ATR	NM_001184	c.632T>C	p.M211T	0.493
Ctrl1	AXIN2	NM_004655	c.148C>T	p.P50S	0.478
Ctrl1	BMPR1A	NM_004329	c.4C>A	p.P2T	1.000
Ctrl1	BRCA1	NM_007294	c.3113A>G	p.E1038G	1.000
Ctrl1	BRCA1	NM_007294	c.3548A>G	p.K1183R	1.000
Ctrl1	BRCA1	NM_007294	c.4837A>G	p.S1613G	1.000
Ctrl1	BRCA1	NM_007294	c.2612C>T	p.P871L	1.000
Ctrl1	BRCA2	NM_000059	c.7397T>C	p.V2466A	1.000
Ctrl1	BRIP1	NM_032043	c.2755T>C	p.S919P	1.000
Ctrl1	CHEK1	NM_001114121	c.1411A>G	p.I471V	1.000
Ctrl1	EPCAM	NM_002354	c.344T>C	p.M115T	1.000
Ctrl1	HNF1A	NM_000545	c.79A>C	p.I27L	1.000
Ctrl1	HNF1A	NM_000545	c.1720A>G	p.S574G	1.000
Ctrl1	HNF1A	NM_000545	c.1460G>A	p.S487N	0.999
Ctrl1	MUTYH	NM_012222	c.1005G>C	p.Q335H	0.485

Ctrl1	NBN	NM_002485	c.553G>C	p.E185Q	0.446
Ctrl1	NOTCH2	NM_024408	c.5065A>T	p.I1689F	0.495
Ctrl1	PMS2	NM_000535	c.1454C>A	p.T485K	0.490
Ctrl1	PMS2	NM_000535	c.1621A>G	p.K541E	1.000
Ctrl1	PMS2	NM_000535	c.1408C>T	p.P470S	0.506
Ctrl1	POLD1	NM_002691	c.356G>A	p.R119H	0.501
Ctrl1	TP53	NM_000546	c.215C>G	p.P72R	0.999
Ctrl1	TSC2	NM_000548	c.856A>G	p.M286V	0.522
Ctrl1	TSC2	NM_000548	c.3421G>A	p.A1141T	0.474
Ctrl2	NOTCH3	NM_000435.2	c.3607G>A	p.E1203K	0.007
Ctrl2	GRIN2A	NM_000833.4	c.3217G>A	p.E1073K	0.006
Ctrl2	MGA	NM_001080541.2	c.3744delA	p.E1249fs	0.005
Ctrl2	SMARCA4	NM_001128844.1	c.2858delA	p.K953fs	0.006
Ctrl2	AXIN1	NM_003502.3	c.421G>T	p.A141S	0.522
Ctrl2	HIST1H3B	NM_003537.3	c.131C>T	p.P44L	0.006
Ctrl2	EPHA3	NM_005233.5	c.1607C>T	p.S536F	0.010
Ctrl2	IRS1	NM_005544.2	c.109G>A	p.E37K	0.006
Ctrl2	ARID1A	NM_006015.5	c.492_494delCGC	p.A165del	0.012
Ctrl2	KAT6A	NM_006766.4	c.3506G>A	p.R1169Q	0.006
Ctrl2	NUP93	NM_014669.4	c.2097delT	p.F699fs	0.006
Ctrl2	KDM6A	NM_021140.3	c.1999C>T	p.Q667*	0.006
Ctrl2	FANCD2	NM_033084.4	c.2613A>C	p.K871N	0.010
Ctrl2	AMER1	NM_152424.4	c.800C>T	p.S267L	0.008
Ctrl2	KIT	NM_000222.2	c.1718C>T	p.P573L	0.009
Ctrl2	APC	NM_000038	c.5465T>A	p.V1822D	0.999
Ctrl2	ATM	NM_000051	c.5948A>G	p.N1983S	0.999
Ctrl2	ATR	NM_001184	c.632T>C	p.M211T	0.483
Ctrl2	BLM	NM_000057	c.893C>T	p.T298M	0.466
Ctrl2	BMPR1A	NM_004329	c.4C>A	p.P2T	1.000
Ctrl2	BRCA1	NM_007294	c.4837A>G	p.S1613G	0.999
Ctrl2	BRCA1	NM_007294	c.3113A>G	p.E1038G	1.000
Ctrl2	BRCA1	NM_007294	c.3548A>G	p.K1183R	0.999
Ctrl2	BRCA1	NM_007294	c.2612C>T	p.P871L	1.000
Ctrl2	BRCA2	NM_000059	c.1114A>C	p.N372H	0.507
Ctrl2	BRCA2	NM_000059	c.7397T>C	p.V2466A	1.000
Ctrl2	BRIP1	NM_032043	c.430G>A	p.A144T	0.495
Ctrl2	BRIP1	NM_032043	c.2755T>C	p.S919P	1.000
Ctrl2	CHEK1	NM_001114121	c.1411A>G	p.I471V	1.000
Ctrl2	EPCAM	NM_002354	c.344T>C	p.M115T	0.491
Ctrl2	HNF1A	NM_000545	c.79A>C	p.I27L	1.000
Ctrl2	HNF1A	NM_000545	c.1460G>A	p.S487N	0.998
Ctrl2	HNF1A	NM_000545	c.1720A>G	p.S574G	1.000
Ctrl2	NBN	NM_002485	c.553G>C	p.E185Q	0.482
Ctrl2	PMS2	NM_000535	c.1454C>A	p.T485K	0.492
Ctrl2	PMS2	NM_000535	c.1621A>G	p.K541E	0.999
Ctrl2	PMS2	NM_000535	c.1408C>T	p.P470S	0.492
Ctrl2	TP53	NM_000546	c.215C>G	p.P72R	0.999
Ctrl3	KIT	NM_000222.2	c.1718C>T	p.P573L	0.013
Ctrl3	KIT	NM_000222.2	c.2341G>A	p.A781T	0.005
Ctrl3	HNF1A	NM_000545.6	c.1010G>A	p.S337N	0.026
Ctrl3	PTGS2	NM_000963.3	c.1516G>A	p.E506K	0.010
Ctrl3	RUNX1	NM_001001890.2	c.1187G>A	p.R396H	0.013
Ctrl3	PLK2	NM_001252226.1	c.2008T>C	p.C670R	0.027
Ctrl3	KLF4	NM_001314052	c.469G>A	p.G157R	0.008
Ctrl3	NTHL1	NM_001318193.1	c.173C>T	p.P58L	0.005
Ctrl3	LYN	NM_002350.3	c.521C>T	p.P174L	0.013

Ctrl3	SPTA1	NM_003126.2	c.1055G>A	p.W352*	0.012
Ctrl3	TGFBR2	NM_003242.5	c.374A>G	p.E125G	0.012
Ctrl3	BCL2L2	NM_004050.4	c.262C>T	p.Q88*	0.013
Ctrl3	PIK3C2G	NM_004570.5	c.4180G>A	p.E1394K	0.015
Ctrl3	MTOR	NM_004958.3	c.2444A>G	p.K815R	0.028
Ctrl3	SETD2	NM_014159.6	c.5014G>A	p.G1672R	0.014
Ctrl3	ASXL1	NM_015338.5	c.1257_1258delAG	p.R419fs	0.020
Ctrl3	ARID1B	NM_017519.2	c.811G>T	p.A271S	0.013
Ctrl3	ZNF703	NM_025069.2	c.1517C>G	p.A506G	0.018
Ctrl3	SLX4	NM_032444.3	c.3761C>T	p.S1254L	0.013
Ctrl3	DOT1L	NM_032482.2	c.2269C>T	p.R757W	0.014
Ctrl3	TAF1	NM_138923.3	c.1316C>T	p.S439F	0.012
Ctrl3	APC	NM_000038	c.5465T>A	p.V1822D	1.000
Ctrl3	ATM	NM_000051	c.5948A>G	p.N1983S	1.000
Ctrl3	ATR	NM_001184	c.632T>C	p.M211T	1.000
Ctrl3	AXIN2	NM_004655	c.148C>T	p.P50S	0.487
Ctrl3	BLM	NM_000057	c.893C>T	p.T298M	0.451
Ctrl3	BRCA1	NM_007294	c.3113A>G	p.E1038G	0.474
Ctrl3	BRCA1	NM_007294	c.3548A>G	p.K1183R	0.478
Ctrl3	BRCA1	NM_007294	c.4837A>G	p.S1613G	0.500
Ctrl3	BRCA1	NM_007294	c.2612C>T	p.P871L	0.510
Ctrl3	BRCA2	NM_000059	c.1114A>C	p.N372H	0.496
Ctrl3	BRCA2	NM_000059	c.7397T>C	p.V2466A	1.000
Ctrl3	BRIP1	NM_032043	c.2755T>C	p.S919P	0.998
Ctrl3	CHEK1	NM_001114121	c.1411A>G	p.I471V	1.000
Ctrl3	EPCAM	NM_002354	c.344T>C	p.M115T	0.998
Ctrl3	HNF1A	NM_000545	c.1720A>G	p.S574G	0.999
Ctrl3	HNF1A	NM_000545	c.79A>C	p.I27L	0.502
Ctrl3	HNF1A	NM_000545	c.1460G>A	p.S487N	0.997
Ctrl3	KIT	NM_000222	c.1621A>C	p.M541L	0.483
Ctrl3	NBN	NM_002485	c.553G>C	p.E185Q	0.495
Ctrl3	PALB2	NM_024675	c.1676A>G	p.Q559R	0.440
Ctrl3	PMS2	NM_000535	c.1621A>G	p.K541E	1.000
Ctrl3	PMS2	NM_000535	c.1454C>A	p.T485K	0.507
Ctrl3	PMS2	NM_000535	c.1408C>T	p.P470S	0.508
Ctrl3	TP53	NM_000546	c.215C>G	p.P72R	0.996
Ctrl3	TSC2	NM_000548	c.3475C>T	p.R1159W	0.499
Ctrl4	INPP4A	NM_001134224.1	c.2198G>A	p.S733N	0.515
Ctrl4	RBM10	NM_001204468.1	c.4T>C	p.S2P	0.519
Ctrl4	EP300	NM_001429.3	c.7100C>T	p.P2367L	0.445
Ctrl4	RPTOR	NM_020761.2	c.2082G>T	p.L694F	0.463
Ctrl4	APC	NM_000038	c.5465T>A	p.V1822D	0.996
Ctrl4	ATM	NM_000051	c.5948A>G	p.N1983S	1.000
Ctrl4	ATR	NM_001184	c.632T>C	p.M211T	0.475
Ctrl4	AXIN2	NM_004655	c.148C>T	p.P50S	0.479
Ctrl4	BARD1	NM_000465	c.70C>T	p.P24S	1.000
Ctrl4	BARD1	NM_000465	c.1134G>C	p.R378S	1.000
Ctrl4	BARD1	NM_000465	c.1519G>A	p.V507M	1.000
Ctrl4	BLM	NM_000057	c.893C>T	p.T298M	0.429
Ctrl4	BMPR1A	NM_004329	c.4C>A	p.P2T	0.443
Ctrl4	BRCA1	NM_007294	c.2612C>T	p.P871L	1.000
Ctrl4	BRCA1	NM_007294	c.3113A>G	p.E1038G	1.000
Ctrl4	BRCA1	NM_007294	c.4837A>G	p.S1613G	1.000
Ctrl4	BRCA1	NM_007294	c.3548A>G	p.K1183R	1.000
Ctrl4	BRCA2	NM_000059	c.865A>C	p.N289H	0.546
Ctrl4	BRCA2	NM_000059	c.7397T>C	p.V2466A	1.000

Ctrl4	BRCA2	NM_000059	c.2971A>G	p.N991D	0.500
Ctrl4	BRIP1	NM_032043	c.2755T>C	p.S919P	1.000
Ctrl4	CHEK1	NM_001114121	c.1411A>G	p.I471V	1.000
Ctrl4	EP300	NM_001429	c.6481A>G	p.M2161V	0.499
Ctrl4	EPCAM	NM_002354	c.344T>C	p.M115T	1.000
Ctrl4	HNF1A	NM_000545	c.1720A>G	p.S574G	0.997
Ctrl4	MUTYH	NM_012222	c.1005G>C	p.Q335H	0.495
Ctrl4	NBN	NM_002485	c.553G>C	p.E185Q	0.511
Ctrl4	PALB2	NM_024675	c.1676A>G	p.Q559R	0.604
Ctrl4	PMS2	NM_000535	c.2570G>C	p.G857A	0.410
Ctrl4	PMS2	NM_000535	c.1408C>T	p.P470S	0.472
Ctrl4	PMS2	NM_000535	c.1454C>A	p.T485K	0.550
Ctrl4	PMS2	NM_000535	c.1621A>G	p.K541E	1.000
Ctrl4	POLD1	NM_002691	c.1932C>G	p.D644E	0.428
Ctrl4	TP53	NM_000546	c.215C>G	p.P72R	0.534
Ctrl5	AR	NM_000044.4	c.1415_1420delGCGGCG	p.G472_G473del	0.333
Ctrl5	HGF	NM_000601.5	c.1807G>A	p.G603R	0.048
Ctrl5	ERG	NM_001136154.1	c.175C>T	p.P59S	0.082
Ctrl5	LNPEP	NM_005575.2	c.1795C>T	p.Q599*	0.217
Ctrl5	NCOR1	NM_006311.3	c.98G>A	p.R33H	0.074
Ctrl5	CYLD	NM_015247.2	c.2020G>A	p.G674R	0.069
Ctrl5	TERT	NM_198253.2	c.1597G>A	p.E533K	0.048
Ctrl5	FGFR4	NM_213647.2	c.1209_1212delCGCC	p.A404fs	0.083
Ctrl5	APC	NM_000038	c.5465T>A	p.V1822D	1.000
Ctrl5	ATM	NM_000051	c.5948A>G	p.N1983S	1.000
Ctrl5	ATR	NM_001184	c.632T>C	p.M211T	0.482
Ctrl5	ATR	NM_001184	c.4165G>T	p.D1389Y	0.482
Ctrl5	BARD1	NM_000465	c.70C>T	p.P24S	1.000
Ctrl5	BARD1	NM_000465	c.1519G>A	p.V507M	1.000
Ctrl5	BARD1	NM_000465	c.1134G>C	p.R378S	1.000
Ctrl5	BMPR1A	NM_004329	c.4C>A	p.P2T	1.000
Ctrl5	BRCA2	NM_000059	c.7397T>C	p.V2466A	1.000
Ctrl5	BRCA2	NM_000059	c.1114A>C	p.N372H	0.554
Ctrl5	BRIP1	NM_032043	c.2755T>C	p.S919P	1.000
Ctrl5	CHEK1	NM_001114121	c.1411A>G	p.I471V	1.000
Ctrl5	EPCAM	NM_002354	c.344T>C	p.M115T	0.507
Ctrl5	HNF1A	NM_000545	c.79A>C	p.I27L	1.000
Ctrl5	HNF1A	NM_000545	c.1460G>A	p.S487N	0.982
Ctrl5	HNF1A	NM_000545	c.1720A>G	p.S574G	1.000
Ctrl5	MSH6	NM_000179	c.116G>A	p.G39E	0.583
Ctrl5	MUTYH	NM_012222	c.1005G>C	p.Q335H	0.516
Ctrl5	PMS2	NM_000535	c.1621A>G	p.K541E	1.000
Ctrl5	PMS2	NM_000535	c.2570G>C	p.G857A	0.638
Ctrl5	PMS2	NM_000535	c.1454C>A	p.T485K	0.606
Ctrl5	PMS2	NM_000535	c.1408C>T	p.P470S	0.377
Ctrl5	POLD1	NM_002691	c.56G>A	p.R19H	0.516
Ctrl7	NF1	NM_001042492.2	c.688G>A	p.E230K	0.044
Ctrl7	SMARCA4	NM_001128844.1	c.1273C>T	p.R425W	0.036
Ctrl7	SMARCA4	NM_001128844.1	c.1283C>T	p.T428I	0.042
Ctrl7	SMARCA4	NM_001128844.1	c.1286C>T	p.A429V	0.035
Ctrl7	TNFAIP3	NM_001270508.1	c.2274delC	p.K759fs	0.035
Ctrl7	CHD4	NM_001297553.1	c.1931A>T	p.Q644L	0.027
Ctrl7	H3F3A	NM_002107.4	c.386G>A	p.R129H	0.020
Ctrl7	RBM10	NM_005676.4	c.363G>T	p.E121D	0.027
Ctrl7	RYBP	NM_012234.6	c.10delA	p.R4fs	0.017
Ctrl7	ZNF703	NM_025069.2	c.1578delC	p.G527fs	0.023

Ctrl7	FLCN	NM_144997.6	c.1285dupC	p.H429fs	0.085
Ctrl7	FGFR4	NM_213647.2	c.1209_1212delCGCC	p.A404fs	0.085
Ctrl7	APC	NM_000038	c.5465T>A	p.V1822D	1.000
Ctrl7	ATM	NM_000051	c.5948A>G	p.N1983S	1.000
Ctrl7	ATR	NM_001184	c.632T>C	p.M211T	0.538
Ctrl7	BLM	NM_000057	c.893C>T	p.T298M	0.518
Ctrl7	BLM	NM_000057	c.3961G>A	p.V1321I	0.506
Ctrl7	BMPR1A	NM_004329	c.4C>A	p.P2T	1.000
Ctrl7	BRCA1	NM_007294	c.4837A>G	p.S1613G	1.000
Ctrl7	BRCA1	NM_007294	c.3548A>G	p.K1183R	1.000
Ctrl7	BRCA1	NM_007294	c.2612C>T	p.P871L	1.000
Ctrl7	BRCA1	NM_007294	c.3113A>G	p.E1038G	1.000
Ctrl7	BRCA2	NM_000059	c.1114A>C	p.N372H	0.557
Ctrl7	BRCA2	NM_000059	c.7397T>C	p.V2466A	1.000
Ctrl7	BRIP1	NM_032043	c.2755T>C	p.S919P	0.517
Ctrl7	CHEK1	NM_001114121	c.1411A>G	p.I471V	1.000
Ctrl7	EPCAM	NM_002354	c.344T>C	p.M115T	1.000
Ctrl7	HNF1A	NM_000545	c.79A>C	p.I27L	0.615
Ctrl7	HNF1A	NM_000545	c.1460G>A	p.S487N	0.575
Ctrl7	HNF1A	NM_000545	c.1720A>G	p.S574G	1.000
Ctrl7	MSH6	NM_000179	c.116G>A	p.G39E	0.464
Ctrl7	MUTYH	NM_012222	c.1005G>C	p.Q335H	0.496
Ctrl7	PMS2	NM_000535	c.1621A>G	p.K541E	0.993
Ctrl7	PMS2	NM_000535	c.1454C>A	p.T485K	1.000
Ctrl7	POLD1	NM_002691	c.356G>A	p.R119H	0.376
Ctrl7	RAD51D	NM_002878	c.494G>A	p.R165Q	0.467
Ctrl7	TP53	NM_000546	c.215C>G	p.P72R	0.563
Ctrl7	TSC1	NM_000368	c.965T>C	p.M322T	0.487
Ctrl9	NRDC	NM_002525.2	c.2716G>A	p.G906S	0.471
Ctrl9	PTPRD	NM_002839.3	c.5693G>A	p.R1898H	0.010
Ctrl9	MED12	NM_005120.2	c.1234G>A	p.A412T	0.468
Ctrl9	IRS1	NM_005544.2	c.109G>A	p.E37K	0.006
Ctrl9	KAT6A	NM_006766.4	c.3506G>A	p.R1169Q	0.006
Ctrl9	SLX4	NM_032444.3	c.929G>A	p.R310Q	0.006
Ctrl9	KLHL6	NM_130446.3	c.253T>A	p.C85S	0.522
Ctrl9	PTPRS	NM_130855.2	c.2399A>T	p.Q800L	0.474
Ctrl9	APC	NM_000038	c.5465T>A	p.V1822D	1.000
Ctrl9	ATM	NM_000051	c.5948A>G	p.N1983S	0.999
Ctrl9	ATR	NM_001184	c.7274G>A	p.R2425Q	0.516
Ctrl9	ATR	NM_001184	c.632T>C	p.M211T	0.493
Ctrl9	BARD1	NM_000465	c.1519G>A	p.V507M	1.000
Ctrl9	BARD1	NM_000465	c.70C>T	p.P24S	0.999
Ctrl9	BARD1	NM_000465	c.1134G>C	p.R378S	1.000
Ctrl9	BMPR1A	NM_004329	c.4C>A	p.P2T	1.000
Ctrl9	BRCA1	NM_007294	c.2612C>T	p.P871L	0.474
Ctrl9	BRCA1	NM_007294	c.3113A>G	p.E1038G	0.494
Ctrl9	BRCA1	NM_007294	c.3548A>G	p.K1183R	0.490
Ctrl9	BRCA1	NM_007294	c.4837A>G	p.S1613G	0.495
Ctrl9	BRCA2	NM_000059	c.7397T>C	p.V2466A	1.000
Ctrl9	BRIP1	NM_032043	c.2755T>C	p.S919P	1.000
Ctrl9	CHEK1	NM_001114121	c.1411A>G	p.I471V	1.000
Ctrl9	EPCAM	NM_002354	c.344T>C	p.M115T	0.511
Ctrl9	HNF1A	NM_000545	c.1460G>A	p.S487N	0.463
Ctrl9	HNF1A	NM_000545	c.1720A>G	p.S574G	1.000
Ctrl9	HNF1A	NM_000545	c.79A>C	p.I27L	0.487
Ctrl9	MSH6	NM_000179	c.116G>A	p.G39E	0.486

Ctrl9	MUTYH	NM_012222	c.1005G>C	p.Q335H	0.466
Ctrl9	NBN	NM_002485	c.553G>C	p.E185Q	0.455
Ctrl9	PALB2	NM_024675	c.1676A>G	p.Q559R	0.480
Ctrl9	PMS2	NM_000535	c.1408C>T	p.P470S	1.000
Ctrl9	PMS2	NM_000535	c.2570G>C	p.G857A	0.996
Ctrl9	PMS2	NM_000535	c.1621A>G	p.K541E	1.000
Ctrl9	POLD1	NM_002691	c.56G>A	p.R19H	0.471
Ctrl9	POLD1	NM_002691	c.356G>A	p.R119H	0.507
Ctrl9	RAD52	NM_001297419	c.1037C>A	p.S346*	0.490
Ctrl9	STK11	NM_000455	c.1062C>G	p.F354L	0.466
Ctrl9	TERT	NM_198253	c.838G>A	p.E280K	0.477
Ctrl10	TET2	NM_001127208.2	c.2830_2832delAAG	p.K944del	0.454
Ctrl10	MET	NM_001127500.2	c.1642T>G	p.S548A	0.492
Ctrl10	TAPBPL	NM_018009.4	c.428T>C	p.M143T	0.476
Ctrl10	APC	NM_000038	c.5465T>A	p.V1822D	0.999
Ctrl10	ATM	NM_000051	c.5948A>G	p.N1983S	1.000
Ctrl10	ATR	NM_001184	c.632T>C	p.M211T	0.496
Ctrl10	AXIN2	NM_004655	c.148C>T	p.P50S	0.476
Ctrl10	BARD1	NM_000465	c.70C>T	p.P24S	0.425
Ctrl10	BARD1	NM_000465	c.1134G>C	p.R378S	0.474
Ctrl10	BARD1	NM_000465	c.1519G>A	p.V507M	0.473
Ctrl10	BLM	NM_000057	c.2293G>A	p.V765I	0.485
Ctrl10	BMPR1A	NM_004329	c.4C>A	p.P2T	0.448
Ctrl10	BRCA1	NM_007294	c.3548A>G	p.K1183R	0.477
Ctrl10	BRCA1	NM_007294	c.3113A>G	p.E1038G	0.501
Ctrl10	BRCA1	NM_007294	c.4837A>G	p.S1613G	0.483
Ctrl10	BRCA1	NM_007294	c.2612C>T	p.P871L	0.485
Ctrl10	BRCA2	NM_000059	c.7397T>C	p.V2466A	1.000
Ctrl10	BRIP1	NM_032043	c.2755T>C	p.S919P	0.480
Ctrl10	CHEK1	NM_001114121	c.1411A>G	p.I471V	1.000
Ctrl10	EP300	NM_001429	c.1519A>G	p.S507G	0.484
Ctrl10	EPCAM	NM_002354	c.344T>C	p.M115T	1.000
Ctrl10	HNF1A	NM_000545	c.1460G>A	p.S487N	0.509
Ctrl10	HNF1A	NM_000545	c.1720A>G	p.S574G	0.999
Ctrl10	HNF1A	NM_000545	c.79A>C	p.I27L	0.467
Ctrl10	MSH6	NM_000179	c.116G>A	p.G39E	0.474
Ctrl10	MUTYH	NM_012222	c.1005G>C	p.Q335H	0.479
Ctrl10	NBN	NM_002485	c.553G>C	p.E185Q	1.000
Ctrl10	PMS2	NM_000535	c.1408C>T	p.P470S	0.489
Ctrl10	PMS2	NM_000535	c.1454C>A	p.T485K	0.501
Ctrl10	PMS2	NM_000535	c.1621A>G	p.K541E	1.000
Ctrl10	POLD1	NM_002691	c.356G>A	p.R119H	0.504
Ctrl10	TP53	NM_000546	c.215C>G	p.P72R	1.000
Ctrl12	BRCA2	NM_000059.3	c.4829_4830delTG	p.V1610fs	0.446
Ctrl12	DMD	NM_000109.3	c.3770G>C	p.W1257S	1.000
Ctrl12	CHD2	NM_001042572.2	c.1207C>T	p.R403W	0.471
Ctrl12	NRDC	NM_002525.2	c.615_617delAAC	p.K205_T206delinsN	0.401
Ctrl12	SLIT2	NM_004787.3	c.2945T>C	p.F982S	0.437
Ctrl12	APC	NM_000038	c.5465T>A	p.V1822D	1.000
Ctrl12	ATM	NM_000051	c.5948A>G	p.N1983S	1.000
Ctrl12	BARD1	NM_000465	c.1134G>C	p.R378S	0.495
Ctrl12	BARD1	NM_000465	c.70C>T	p.P24S	0.493
Ctrl12	BARD1	NM_000465	c.1519G>A	p.V507M	0.468
Ctrl12	BMPR1A	NM_004329	c.4C>A	p.P2T	0.429
Ctrl12	BRCA1	NM_007294	c.3548A>G	p.K1183R	0.999
Ctrl12	BRCA1	NM_007294	c.2612C>T	p.P871L	1.000

Ctrl12	BRCA1	NM_007294	c.4837A>G	p.S1613G	1.000
Ctrl12	BRCA1	NM_007294	c.3113A>G	p.E1038G	0.999
Ctrl12	BRCA2	NM_000059	c.4829_4830delTG	p.V1610fs	0.446
Ctrl12	BRCA2	NM_000059	c.865A>C	p.N289H	0.452
Ctrl12	BRCA2	NM_000059	c.2971A>G	p.N991D	0.486
Ctrl12	BRCA2	NM_000059	c.7397T>C	p.V2466A	1.000
Ctrl12	CHEK1	NM_001114121	c.1411A>G	p.I471V	1.000
Ctrl12	EPCAM	NM_002354	c.344T>C	p.M115T	0.999
Ctrl12	HNF1A	NM_000545	c.1720A>G	p.S574G	0.999
Ctrl12	HNF1A	NM_000545	c.1460G>A	p.S487N	0.504
Ctrl12	HNF1A	NM_000545	c.79A>C	p.I27L	0.430
Ctrl12	MSH6	NM_000179	c.116G>A	p.G39E	0.473
Ctrl12	MSH6	NM_000179	c.4068_4071dupGATT	p.K1358fs	0.380
Ctrl12	MUTYH	NM_012222	c.1005G>C	p.Q335H	0.481
Ctrl12	NBN	NM_002485	c.553G>C	p.E185Q	0.458
Ctrl12	NBN	NM_002485	c.1690G>A	p.E564K	0.491
Ctrl12	PMS2	NM_000535	c.2570G>C	p.G857A	0.327
Ctrl12	PMS2	NM_000535	c.1621A>G	p.K541E	0.999
Ctrl12	POLD1	NM_002691	c.56G>A	p.R19H	0.495
Ctrl12	TP53	NM_000546	c.215C>G	p.P72R	1.000
Ctrl13	EP300	NM_001429.3	c.773C>T	p.T258I	0.532
Ctrl13	ETV6	NM_001987.4	c.1067A>G	p.N356S	0.534
Ctrl13	AXIN1	NM_003502.3	c.2389C>T	p.R797C	0.479
Ctrl13	CEBPA	NM_004364.4	c.427C>G	p.L143V	0.475
Ctrl13	EPHA5	NM_004439.7	c.1288G>A	p.D430N	0.535
Ctrl13	RRAS	NM_006270.4	c.640C>T	p.P214S	0.443
Ctrl13	MUTYH	NM_012222.2	c.917G>A	p.R306H	0.455
Ctrl13	SPEN	NM_015001.2	c.4718C>G	p.T1573S	0.456
Ctrl13	APC	NM_000038	c.5465T>A	p.V1822D	1.000
Ctrl13	ATM	NM_000051	c.5948A>G	p.N1983S	1.000
Ctrl13	ATR	NM_001184	c.7274G>A	p.R2425Q	0.496
Ctrl13	ATR	NM_001184	c.632T>C	p.M211T	1.000
Ctrl13	ATR	NM_001184	c.3637A>G	p.S1213G	0.471
Ctrl13	BARD1	NM_000465	c.1134G>C	p.R378S	0.998
Ctrl13	BARD1	NM_000465	c.1519G>A	p.V507M	1.000
Ctrl13	BARD1	NM_000465	c.70C>T	p.P24S	1.000
Ctrl13	BMPR1A	NM_004329	c.4C>A	p.P2T	0.997
Ctrl13	BRCA1	NM_007294	c.3548A>G	p.K1183R	1.000
Ctrl13	BRCA1	NM_007294	c.4837A>G	p.S1613G	1.000
Ctrl13	BRCA1	NM_007294	c.3113A>G	p.E1038G	1.000
Ctrl13	BRCA1	NM_007294	c.2612C>T	p.P871L	1.000
Ctrl13	BRCA2	NM_000059	c.7397T>C	p.V2466A	0.998
Ctrl13	BRIP1	NM_032043	c.2755T>C	p.S919P	0.998
Ctrl13	CHEK1	NM_001114121	c.1411A>G	p.I471V	1.000
Ctrl13	EP300	NM_001429	c.2989A>G	p.I997V	0.492
Ctrl13	EPCAM	NM_002354	c.344T>C	p.M115T	0.532
Ctrl13	HNF1A	NM_000545	c.1720A>G	p.S574G	1.000
Ctrl13	HNF1A	NM_000545	c.1460G>A	p.S487N	1.000
Ctrl13	HNF1A	NM_000545	c.79A>C	p.I27L	1.000
Ctrl13	MSH2	NM_000251	c.1168C>T	p.L390F	0.589
Ctrl13	MUTYH	NM_012222	c.1109C>T	p.A370V	0.480
Ctrl13	NBN	NM_002485	c.553G>C	p.E185Q	0.529
Ctrl13	PMS2	NM_000535	c.1621A>G	p.K541E	1.000
Ctrl13	PMS2	NM_000535	c.1408C>T	p.P470S	0.471
Ctrl13	PMS2	NM_000535	c.1454C>A	p.T485K	0.531
Ctrl13	POLE	NM_006231	c.6494G>A	p.R2165H	0.511

Ctrl13	STK11	NM_000455	c.1062C>G	p.F354L	0.420
Ctrl14	AR	NM_000044.4	c.331G>A	p.D111N	0.046
Ctrl14	KIT	NM_000222.2	c.2287G>T	p.A763S	0.015
Ctrl14	PTCH1	NM_000264.4	c.3945delC	p.Y1316fs	0.015
Ctrl14	TSC2	NM_000548.4	c.700G>A	p.E234K	0.016
Ctrl14	GRIN2A	NM_000833.4	c.1576G>A	p.V526M	0.015
Ctrl14	ESR1	NM_001122742.1	c.296dupC	p.L100fs	0.014
Ctrl14	INPP4A	NM_001134224.1	c.1934G>C	p.S645T	0.505
Ctrl14	SYK	NM_001135052.3	c.98dupG	p.M34fs	0.013
Ctrl14	ERG	NM_001136154.1	c.1232delC	p.P411fs	0.017
Ctrl14	KMT5A	NM_001324506.1	c.580T>C	p.C194R	0.018
Ctrl14	INPPL1	NM_001567.3	c.269G>A	p.R90H	0.507
Ctrl14	CANX	NM_001746.3	c.1305dupT	p.D436fs	0.029
Ctrl14	FLT1	NM_002019.4	c.3803C>T	p.S1268L	0.402
Ctrl14	JUN	NM_002228.3	c.617A>C	p.Q206P	0.021
Ctrl14	KDR	NM_002253.3	c.712G>A	p.V238I	0.454
Ctrl14	PLCG2	NM_002661.4	c.1093G>A	p.D365N	0.013
Ctrl14	TGFBR2	NM_003242.5	c.374A>G	p.E125G	0.020
Ctrl14	INPP4B	NM_003866.2	c.344A>G	p.Y115C	0.483
Ctrl14	HERC1	NM_003922.3	c.11122G>A	p.D3708N	0.447
Ctrl14	SLIT2	NM_004787.3	c.4499_4501delGGA	p.R1500_K1501delins	0.017
Ctrl14	PIK3CD	NM_005026.4	c.2108C>T	p.S703F	0.024
Ctrl14	MPL	NM_005373.2	c.695C>T	p.S232L	0.017
Ctrl14	PIK3CB	NM_006219.2	c.1810dupC	p.R604fs	0.022
Ctrl14	NPEPPS	NM_006310.3	c.890A>G	p.N297S	0.345
Ctrl14	SF3B1	NM_012433.3	c.25G>A	p.E9K	0.015
Ctrl14	NOTCH1	NM_017617.5	c.6392dupG	p.T2132fs	0.018
Ctrl14	FBXW7	NM_018315.5	c.139G>A	p.E47K	0.017
Ctrl14	TRAF2	NM_021138.3	c.122T>C	p.L41P	0.015
Ctrl14	ZNF703	NM_025069.2	c.1540G>T	p.A514S	0.014
Ctrl14	TET1	NM_030625.2	c.1171G>A	p.E391K	0.014
Ctrl14	APC	NM_000038	c.5465T>A	p.V1822D	1.000
Ctrl14	ATM	NM_000051	c.5948A>G	p.N1983S	0.989
Ctrl14	ATR	NM_001184	c.3637A>G	p.S1213G	0.482
Ctrl14	ATR	NM_001184	c.632T>C	p.M211T	1.000
Ctrl14	ATR	NM_001184	c.7274G>A	p.R2425Q	0.513
Ctrl14	BARD1	NM_000465	c.1519G>A	p.V507M	1.000
Ctrl14	BARD1	NM_000465	c.70C>T	p.P24S	0.461
Ctrl14	BARD1	NM_000465	c.1134G>C	p.R378S	1.000
Ctrl14	BRCA1	NM_007294	c.2566T>C	p.Y856H	0.525
Ctrl14	BRCA2	NM_000059	c.7397T>C	p.V2466A	1.000
Ctrl14	BRIP1	NM_032043	c.2755T>C	p.S919P	1.000
Ctrl14	BRIP1	NM_032043	c.430G>A	p.A144T	0.495
Ctrl14	CHEK1	NM_001114121	c.1411A>G	p.I471V	1.000
Ctrl14	EPCAM	NM_002354	c.344T>C	p.M115T	1.000
Ctrl14	HNF1A	NM_000545	c.79A>C	p.I27L	0.499
Ctrl14	HNF1A	NM_000545	c.1460G>A	p.S487N	0.520
Ctrl14	HNF1A	NM_000545	c.1720A>G	p.S574G	0.998
Ctrl14	MLH1	NM_000249	c.655A>G	p.I219V	0.500
Ctrl14	NBN	NM_002485	c.553G>C	p.E185Q	0.453
Ctrl14	PALB2	NM_024675	c.1676A>G	p.Q559R	0.465
Ctrl14	PMS2	NM_000535	c.1621A>G	p.K541E	0.490
Ctrl15	CDK4	NM_000075.3	c.763C>T	p.R255C	0.005
Ctrl15	FGFR2	NM_000141.4	c.910G>A	p.D304N	0.012
Ctrl15	LIG1	NM_000234.2	c.897C>G	p.I299M	0.496
Ctrl15	PTCH1	NM_000264.4	c.4181G>A	p.R1394Q	0.008

Ctrl15	NOTCH3	NM_000435.2	c.3607G>A	p.E1203K	0.008
Ctrl15	ATRX	NM_000489.5	c.6235C>T	p.R2079*	0.005
Ctrl15	GRM3	NM_000840.2	c.2024A>T	p.N675I	0.495
Ctrl15	DDR2	NM_001014796.2	c.2141G>A	p.R714Q	0.485
Ctrl15	INPP4A	NM_001134224.1	c.2905G>A	p.E969K	0.010
Ctrl15	ERG	NM_001136154.1	c.1231C>G	p.P411A	0.496
Ctrl15	CD70	NM_001252.4	c.488G>A	p.R163Q	0.011
Ctrl15	CHD2	NM_001271.3	c.4033C>T	p.R1345W	0.482
Ctrl15	EP300	NM_001429.3	c.5485C>T	p.R1829C	0.010
Ctrl15	PARP1	NM_001618.3	c.1832T>C	p.I611T	0.493
Ctrl15	CBFB	NM_001755.2	c.7C>T	p.R3C	0.012
Ctrl15	FLT1	NM_002019.4	c.2890G>A	p.E964K	0.012
Ctrl15	IL7R	NM_002185.4	c.775T>G	p.L259V	0.480
Ctrl15	KDR	NM_002253.3	c.2837G>A	p.R946H	0.488
Ctrl15	TCF3	NM_003200.4	c.937G>A	p.G313R	0.017
Ctrl15	TOP1	NM_003286.3	c.1522C>T	p.R508C	0.005
Ctrl15	TPP2	NM_003291.3	c.2611G>A	p.D871N	0.010
Ctrl15	TP63	NM_003722.4	c.469G>A	p.D157N	0.010
Ctrl15	DMD	NM_004013.2	c.2792G>A	p.R931Q	0.011
Ctrl15	SLIT2	NM_004787.3	c.1162C>T	p.R388W	0.008
Ctrl15	XRCC2	NM_005431.1	c.191G>A	p.R64Q	0.013
Ctrl15	IRS1	NM_005544.2	c.109G>A	p.E37K	0.005
Ctrl15	RBM10	NM_005676.4	c.322C>T	p.R108W	0.010
Ctrl15	IKZF1	NM_006060.6	c.698C>T	p.P233L	0.009
Ctrl15	PDGFRA	NM_006206.5	c.2942G>A	p.R981H	0.476
Ctrl15	ZNF217	NM_006526.2	c.1351G>A	p.E451K	0.009
Ctrl15	KAT6A	NM_006766.4	c.3506G>A	p.R1169Q	0.012
Ctrl15	MAGI2	NM_012301.3	c.325C>T	p.R109C	0.009
Ctrl15	ANKRD11	NM_013275.5	c.6910G>A	p.E2304K	0.015
Ctrl15	SPEN	NM_015001.2	c.8713G>A	p.D2905N	0.006
Ctrl15	RNF43	NM_017763.5	c.1820C>T	p.S607L	0.008
Ctrl15	RET	NM_020975.5	c.1385C>T	p.S462L	0.007
Ctrl15	FANCE	NM_021922.2	c.316C>T	p.R106W	0.006
Ctrl15	BCORL1	NM_021946.4	c.1624G>A	p.D542N	0.010
Ctrl15	ADGRA2	NM_032777.9	c.2020G>A	p.V674M	0.015
Ctrl15	FLT4	NM_182925.4	c.845G>A	p.R282Q	0.020
Ctrl15	ALK	NM_004304	c.1111G>A	p.A371T	0.493
Ctrl15	APC	NM_000038	c.5465T>A	p.V1822D	1.000
Ctrl15	ATM	NM_000051	c.5948A>G	p.N1983S	1.000
Ctrl15	ATR	NM_001184	c.7274G>A	p.R2425Q	0.503
Ctrl15	ATR	NM_001184	c.632T>C	p.M211T	0.999
Ctrl15	AXIN2	NM_004655	c.148C>T	p.P50S	0.466
Ctrl15	BMPR1A	NM_004329	c.4C>A	p.P2T	1.000
Ctrl15	BRCA1	NM_007294	c.3113A>G	p.E1038G	0.483
Ctrl15	BRCA1	NM_007294	c.3548A>G	p.K1183R	0.514
Ctrl15	BRCA1	NM_007294	c.2612C>T	p.P871L	0.484
Ctrl15	BRCA1	NM_007294	c.4837A>G	p.S1613G	0.488
Ctrl15	BRCA2	NM_000059	c.7052C>G	p.A2351G	0.443
Ctrl15	BRCA2	NM_000059	c.1114A>C	p.N372H	0.512
Ctrl15	BRCA2	NM_000059	c.7397T>C	p.V2466A	0.999
Ctrl15	BRIP1	NM_032043	c.2755T>C	p.S919P	1.000
Ctrl15	CHEK1	NM_001114121	c.1411A>G	p.I471V	1.000
Ctrl15	EPCAM	NM_002354	c.344T>C	p.M115T	1.000
Ctrl15	HNF1A	NM_000545	c.1720A>G	p.S574G	0.999
Ctrl15	HNF1A	NM_000545	c.1460G>A	p.S487N	0.999
Ctrl15	HNF1A	NM_000545	c.79A>C	p.I27L	1.000

Ctrl15	MSH6	NM_000179	c.116G>A	p.G39E	0.486
Ctrl15	PALB2	NM_024675	c.1676A>G	p.Q559R	0.486
Ctrl15	PMS2	NM_000535	c.1454C>A	p.T485K	0.506
Ctrl15	PMS2	NM_000535	c.2570G>C	p.G857A	0.492
Ctrl15	PMS2	NM_000535	c.1408C>T	p.P470S	0.486
Ctrl15	PMS2	NM_000535	c.1621A>G	p.K541E	0.999
Ctrl15	POLD1	NM_002691	c.356G>A	p.R119H	0.497
Ctrl15	POLD1	NM_002691	c.56G>A	p.R19H	0.486
Ctrl15	POLE	NM_006231	c.755C>T	p.A252V	0.491
Ctrl15	TP53	NM_000546	c.215C>G	p.P72R	0.495
Ctrl16	DMD	NM_000109.3	c.5992C>G	p.H1998D	1.000
Ctrl16	PRKCI	NM_002740.5	c.844A>G	p.M282V	0.466
Ctrl16	IRS2	NM_003749.2	c.705G>C	p.Q235H	0.481
Ctrl16	AXIN2	NM_004655.4	c.1561C>T	p.H521Y	0.475
Ctrl16	NPEPPS	NM_006310.3	c.682G>A	p.E228K	0.471
Ctrl16	SHQ1	NM_018130.2	c.1605delT	p.P537fs	0.437
Ctrl16	APC	NM_000038	c.5465T>A	p.V1822D	0.488
Ctrl16	ATM	NM_000051	c.5948A>G	p.N1983S	1.000
Ctrl16	ATR	NM_001184	c.632T>C	p.M211T	0.508
Ctrl16	BARD1	NM_000465	c.1134G>C	p.R378S	0.471
Ctrl16	BARD1	NM_000465	c.1519G>A	p.V507M	0.448
Ctrl16	BARD1	NM_000465	c.70C>T	p.P24S	0.493
Ctrl16	BMPR1A	NM_004329	c.4C>A	p.P2T	0.392
Ctrl16	BRCA2	NM_000059	c.1114A>C	p.N372H	0.469
Ctrl16	BRCA2	NM_000059	c.7397T>C	p.V2466A	0.999
Ctrl16	BRIP1	NM_032043	c.2755T>C	p.S919P	0.481
Ctrl16	CHEK1	NM_001114121	c.1411A>G	p.I471V	1.000
Ctrl16	EPCAM	NM_002354	c.344T>C	p.M115T	1.000
Ctrl16	HNF1A	NM_000545	c.1720A>G	p.S574G	1.000
Ctrl16	HNF1A	NM_000545	c.1460G>A	p.S487N	0.458
Ctrl16	HNF1A	NM_000545	c.79A>C	p.I27L	0.472
Ctrl16	MSH6	NM_000179	c.116G>A	p.G39E	0.443
Ctrl16	MUTYH	NM_012222	c.1005G>C	p.Q335H	0.511
Ctrl16	NBN	NM_002485	c.553G>C	p.E185Q	0.446
Ctrl16	PMS2	NM_000535	c.2570G>C	p.G857A	0.339
Ctrl16	PMS2	NM_000535	c.1621A>G	p.K541E	0.479
Ctrl16	POLD1	NM_002691	c.356G>A	p.R119H	0.496
Ctrl16	TSC1	NM_000368	c.965T>C	p.M322T	0.511
Ctrl18	MLH3	NM_001040108.1	c.1189_1191delATT	p.I397del	0.385
Ctrl18	AXIN2	NM_004655.4	c.854T>C	p.I285T	0.501
Ctrl18	CREBBP	NM_004380.2	c.6685G>A	p.G2229S	0.496
Ctrl18	PIK3CB	NM_006219.2	c.2365G>A	p.V789I	0.450
Ctrl18	NOTCH1	NM_017617.5	c.1487G>A	p.S496N	0.471
Ctrl18	APC	NM_000038	c.5465T>A	p.V1822D	1.000
Ctrl18	ATM	NM_000051	c.5948A>G	p.N1983S	1.000
Ctrl18	BARD1	NM_000465	c.1075_1095delTTGCCTGAATGTTCTTCACCA	p.L359_P365del	0.805
Ctrl18	BLM	NM_000057	c.893C>T	p.T298M	0.454
Ctrl18	BMPR1A	NM_004329	c.4C>A	p.P2T	1.000
Ctrl18	BRCA1	NM_007294	c.4837A>G	p.S1613G	0.488
Ctrl18	BRCA1	NM_007294	c.2612C>T	p.P871L	0.499
Ctrl18	BRCA1	NM_007294	c.3548A>G	p.K1183R	0.475
Ctrl18	BRCA1	NM_007294	c.3113A>G	p.E1038G	0.499
Ctrl18	BRCA2	NM_000059	c.7397T>C	p.V2466A	0.999
Ctrl18	BRIP1	NM_032043	c.2755T>C	p.S919P	0.469
Ctrl18	CHEK1	NM_001114121	c.1411A>G	p.I471V	1.000

Ctrl18	EPCAM	NM_002354	c.344T>C	p.M115T	1.000
Ctrl18	EPCAM	NM_002354	c.515C>T	p.T172M	1.000
Ctrl18	HNF1A	NM_000545	c.1460G>A	p.S487N	0.999
Ctrl18	HNF1A	NM_000545	c.1720A>G	p.S574G	0.999
Ctrl18	HNF1A	NM_000545	c.79A>C	p.I27L	1.000
Ctrl18	KIT	NM_000222	c.1621A>C	p.M541L	0.503
Ctrl18	MUTYH	NM_012222	c.1005G>C	p.Q335H	0.488
Ctrl18	PALB2	NM_024675	c.1676A>G	p.Q559R	0.489
Ctrl18	PMS2	NM_000535	c.1621A>G	p.K541E	1.000
Ctrl18	PMS2	NM_000535	c.1408C>T	p.P470S	1.000
Ctrl18	PMS2	NM_000535	c.1532C>T	p.T511M	0.484
Ctrl18	POLE	NM_006231	c.755C>T	p.A252V	0.460
Ctrl18	TP53	NM_000546	c.215C>G	p.P72R	0.999
Ctrl19	FGFR3	NM_001163213.1	c.956C>T	p.A319V	0.496
Ctrl19	TNFAIP3	NM_001270508.1	c.1939A>C	p.T647P	0.509
Ctrl19	PIK3CG	NM_001282426.1	c.2341G>A	p.E781K	0.478
Ctrl19	MSH3	NM_002439.4	c.22delT	p.S8fs	0.492
Ctrl19	HIST1H3A	NM_003529.2	c.15G>C	p.K5N	0.493
Ctrl19	APC	NM_000038	c.5465T>A	p.V1822D	0.999
Ctrl19	ATM	NM_000051	c.5948A>G	p.N1983S	1.000
Ctrl19	ATR	NM_001184	c.325C>T	p.R109W	0.480
Ctrl19	ATR	NM_001184	c.632T>C	p.M211T	0.479
Ctrl19	AXIN2	NM_004655	c.148C>T	p.P50S	1.000
Ctrl19	BARD1	NM_000465	c.70C>T	p.P24S	1.000
Ctrl19	BARD1	NM_000465	c.1519G>A	p.V507M	0.998
Ctrl19	BARD1	NM_000465	c.1134G>C	p.R378S	0.482
Ctrl19	BRCA1	NM_007294	c.4837A>G	p.S1613G	1.000
Ctrl19	BRCA1	NM_007294	c.3113A>G	p.E1038G	1.000
Ctrl19	BRCA1	NM_007294	c.2612C>T	p.P871L	1.000
Ctrl19	BRCA1	NM_007294	c.3548A>G	p.K1183R	1.000
Ctrl19	BRCA2	NM_000059	c.10234A>G	p.I3412V	0.462
Ctrl19	BRCA2	NM_000059	c.7397T>C	p.V2466A	1.000
Ctrl19	BRCA2	NM_000059	c.1114A>C	p.N372H	0.463
Ctrl19	BRCA2	NM_000059	c.8187G>T	p.K2729N	0.497
Ctrl19	BRIP1	NM_032043	c.2755T>C	p.S919P	0.472
Ctrl19	CHEK1	NM_001114121	c.1411A>G	p.I471V	1.000
Ctrl19	EPCAM	NM_002354	c.344T>C	p.M115T	0.508
Ctrl19	EPCAM	NM_002354	c.515C>T	p.T172M	0.454
Ctrl19	HNF1A	NM_000545	c.79A>C	p.I27L	0.497
Ctrl19	HNF1A	NM_000545	c.1460G>A	p.S487N	0.464
Ctrl19	HNF1A	NM_000545	c.1720A>G	p.S574G	1.000
Ctrl19	KIT	NM_000222	c.1621A>C	p.M541L	0.478
Ctrl19	MUTYH	NM_012222	c.1005G>C	p.Q335H	0.999
Ctrl19	NBN	NM_002485	c.553G>C	p.E185Q	0.458
Ctrl19	NSD1	NM_022455	c.7145C>T	p.T2382I	0.491
Ctrl19	PMS2	NM_000535	c.1621A>G	p.K541E	1.000
Ctrl19	PMS2	NM_000535	c.1454C>A	p.T485K	0.484
Ctrl19	PMS2	NM_000535	c.2570G>C	p.G857A	0.493
Ctrl19	POLD1	NM_002691	c.356G>A	p.R119H	0.502
Ctrl19	POLE	NM_006231	c.755C>T	p.A252V	0.454
Ctrl19	TP53	NM_000546	c.215C>G	p.P72R	0.489
Ctrl20	TET2	NM_001127208.2	c.3115T>C	p.S1039P	0.485
Ctrl20	CCNE1	NM_001238.3	c.931G>A	p.E311K	0.486
Ctrl20	PIK3CD	NM_005026.4	c.1394C>T	p.T465M	0.471
Ctrl20	XRCC2	NM_005431.1	c.524A>C	p.K175T	0.475
Ctrl20	ABCB9	NM_019624.3	c.500A>G	p.E167G	0.465

Ctrl20	APC	NM_000038	c.5465T>A	p.V1822D	1.000
Ctrl20	ATM	NM_000051	c.5948A>G	p.N1983S	1.000
Ctrl20	AXIN2	NM_004655	c.148C>T	p.P50S	0.503
Ctrl20	BMPR1A	NM_004329	c.4C>A	p.P2T	0.434
Ctrl20	BRCA2	NM_000059	c.7397T>C	p.V2466A	1.000
Ctrl20	BRCA2	NM_000059	c.865A>C	p.N289H	1.000
Ctrl20	BRCA2	NM_000059	c.6148G>A	p.V2050I	0.498
Ctrl20	BRCA2	NM_000059	c.2971A>G	p.N991D	1.000
Ctrl20	BRIP1	NM_032043	c.2755T>C	p.S919P	1.000
Ctrl20	CHEK1	NM_001114121	c.1411A>G	p.I471V	1.000
Ctrl20	EPCAM	NM_002354	c.344T>C	p.M115T	1.000
Ctrl20	HNF1A	NM_000545	c.1720A>G	p.S574G	1.000
Ctrl20	HNF1A	NM_000545	c.1460G>A	p.S487N	1.000
Ctrl20	MSH2	NM_000251	c.2425G>A	p.E809K	0.477
Ctrl20	NBN	NM_002485	c.553G>C	p.E185Q	0.481
Ctrl20	PMS2	NM_000535	c.1621A>G	p.K541E	1.000
Ctrl20	PMS2	NM_000535	c.59G>A	p.R20Q	0.495
Ctrl20	PMS2	NM_000535	c.2570G>C	p.G857A	0.500
Ctrl20	PMS2	NM_000535	c.1408C>T	p.P470S	0.495
Ctrl20	POLD1	NM_002691	c.356G>A	p.R119H	0.471
Ctrl20	TP53	NM_000546	c.215C>G	p.P72R	1.000
Ctrl21	EZH2	NM_004456.4	c.1544A>G	p.K515R	0.006
Ctrl21	SLIT2	NM_004787.3	c.4544A>T	p.E1515V	0.476
Ctrl21	MEN1	NM_130799.2	c.1169C>T	p.P390L	0.488
Ctrl21	APC	NM_000038	c.5465T>A	p.V1822D	0.493
Ctrl21	ATM	NM_000051	c.5948A>G	p.N1983S	1.000
Ctrl21	ATR	NM_001184	c.632T>C	p.M211T	0.536
Ctrl21	BARD1	NM_000465	c.70C>T	p.P24S	1.000
Ctrl21	BARD1	NM_000465	c.1519G>A	p.V507M	1.000
Ctrl21	BARD1	NM_000465	c.1134G>C	p.R378S	0.998
Ctrl21	BMPR1A	NM_004329	c.4C>A	p.P2T	0.432
Ctrl21	BRCA1	NM_007294	c.3548A>G	p.K1183R	0.999
Ctrl21	BRCA1	NM_007294	c.3113A>G	p.E1038G	0.999
Ctrl21	BRCA1	NM_007294	c.2612C>T	p.P871L	1.000
Ctrl21	BRCA1	NM_007294	c.4837A>G	p.S1613G	1.000
Ctrl21	BRCA2	NM_000059	c.865A>C	p.N289H	1.000
Ctrl21	BRCA2	NM_000059	c.2971A>G	p.N991D	1.000
Ctrl21	BRCA2	NM_000059	c.7397T>C	p.V2466A	1.000
Ctrl21	BRIP1	NM_032043	c.2755T>C	p.S919P	0.506
Ctrl21	CHEK1	NM_001114121	c.1411A>G	p.I471V	1.000
Ctrl21	EP300	NM_001429	c.2989A>G	p.I997V	0.488
Ctrl21	EPCAM	NM_002354	c.344T>C	p.M115T	0.999
Ctrl21	HNF1A	NM_000545	c.1720A>G	p.S574G	1.000
Ctrl21	HNF1A	NM_000545	c.1460G>A	p.S487N	0.445
Ctrl21	HNF1A	NM_000545	c.79A>C	p.I27L	0.999
Ctrl21	MUTYH	NM_012222	c.1005G>C	p.Q335H	0.460
Ctrl21	NBN	NM_002485	c.553G>C	p.E185Q	1.000
Ctrl21	PMS2	NM_000535	c.2570G>C	p.G857A	0.998
Ctrl21	PMS2	NM_000535	c.1621A>G	p.K541E	0.998
Ctrl21	PMS2	NM_000535	c.1408C>T	p.P470S	1.000
Ctrl21	TP53	NM_000546	c.215C>G	p.P72R	0.508
Ctrl22	CD48	NM_001778.3	c.698C>T	p.T233M	0.469
Ctrl22	INPP4B	NM_003866.2	c.1396G>A	p.V466I	0.473
Ctrl22	PHOX2B	NM_003924.3	c.741delC	p.A248fs	0.012
Ctrl22	RANBP2	NM_006267.4	c.1114A>G	p.I372V	0.017
Ctrl22	ERAP1	NM_016442.4	c.2200_2202delCTC	p.L734del	0.443

Ctrl22	AXL	NM_021913.4	c.874delC	p.H292fs	0.005
Ctrl22	APC	NM_000038	c.5465T>A	p.V1822D	1.000
Ctrl22	ATM	NM_000051	c.5948A>G	p.N1983S	1.000
Ctrl22	ATR	NM_001184	c.632T>C	p.M211T	0.486
Ctrl22	AXIN2	NM_004655	c.148C>T	p.P50S	0.471
Ctrl22	BARD1	NM_000465	c.1134G>C	p.R378S	0.999
Ctrl22	BARD1	NM_000465	c.70C>T	p.P24S	0.474
Ctrl22	BARD1	NM_000465	c.1519G>A	p.V507M	1.000
Ctrl22	BMPR1A	NM_004329	c.4C>A	p.P2T	0.443
Ctrl22	BRCA2	NM_000059	c.7397T>C	p.V2466A	0.997
Ctrl22	BRIP1	NM_032043	c.2440C>T	p.R814C	0.445
Ctrl22	BRIP1	NM_032043	c.2755T>C	p.S919P	0.495
Ctrl22	CHEK1	NM_001114121	c.1411A>G	p.I471V	1.000
Ctrl22	EPCAM	NM_002354	c.344T>C	p.M115T	1.000
Ctrl22	HNF1A	NM_000545	c.1720A>G	p.S574G	0.999
Ctrl22	MLH1	NM_000249	c.1151T>A	p.V384D	0.469
Ctrl22	MSH6	NM_000179	c.4068_4071dupGATT	p.K1358fs	0.407
Ctrl22	MSH6	NM_000179	c.116G>A	p.G39E	0.550
Ctrl22	NBN	NM_002485	c.553G>C	p.E185Q	0.488
Ctrl22	PALB2	NM_024675	c.1676A>G	p.Q559R	0.476
Ctrl22	PMS2	NM_000535	c.1621A>G	p.K541E	1.000
Ctrl22	PMS2	NM_000535	c.1454C>A	p.T485K	0.998
Ctrl22	POLD1	NM_002691	c.356G>A	p.R119H	0.494
Ctrl22	TP53	NM_000546	c.215C>G	p.P72R	0.999
Ctrl22	TSC2	NM_000548	c.2032G>A	p.A678T	0.500
Ctrl23	PGR	NM_000926.4	c.1570G>T	p.G524C	0.380
Ctrl23	MAP3K1	NM_005921.1	c.745C>T	p.R249C	0.425
Ctrl23	HOXB13	NM_006361.5	c.493C>T	p.P165S	0.500
Ctrl23	APC	NM_000038	c.5465T>A	p.V1822D	1.000
Ctrl23	ATM	NM_000051	c.5948A>G	p.N1983S	1.000
Ctrl23	ATR	NM_001184	c.632T>C	p.M211T	1.000
Ctrl23	ATR	NM_001184	c.7274G>A	p.R2425Q	0.571
Ctrl23	AXIN2	NM_004655	c.148C>T	p.P50S	0.980
Ctrl23	BARD1	NM_000465	c.1519G>A	p.V507M	0.519
Ctrl23	BARD1	NM_000465	c.70C>T	p.P24S	0.410
Ctrl23	BARD1	NM_000465	c.1134G>C	p.R378S	0.500
Ctrl23	BRCA1	NM_007294	c.3113A>G	p.E1038G	1.000
Ctrl23	BRCA1	NM_007294	c.2612C>T	p.P871L	0.923
Ctrl23	BRCA1	NM_007294	c.4837A>G	p.S1613G	1.000
Ctrl23	BRCA1	NM_007294	c.3548A>G	p.K1183R	1.000
Ctrl23	BRCA2	NM_000059	c.7397T>C	p.V2466A	1.000
Ctrl23	BRIP1	NM_032043	c.2755T>C	p.S919P	0.546
Ctrl23	CHEK1	NM_001114121	c.1411A>G	p.I471V	1.000
Ctrl23	EP300	NM_001429	c.2989A>G	p.I997V	0.435
Ctrl23	HNF1A	NM_000545	c.1460G>A	p.S487N	0.560
Ctrl23	HNF1A	NM_000545	c.79A>C	p.I27L	0.490
Ctrl23	HNF1A	NM_000545	c.1720A>G	p.S574G	1.000
Ctrl23	MUTYH	NM_012222	c.1005G>C	p.Q335H	0.482
Ctrl23	NBN	NM_002485	c.553G>C	p.E185Q	0.810
Ctrl23	PALB2	NM_024675	c.1676A>G	p.Q559R	0.520
Ctrl23	PMS2	NM_000535	c.1621A>G	p.K541E	1.000
Ctrl23	PMS2	NM_000535	c.2570G>C	p.G857A	0.500
Ctrl23	PMS2	NM_000535	c.1454C>A	p.T485K	1.000
Ctrl23	POLD1	NM_002691	c.356G>A	p.R119H	0.449
Ctrl23	RAD51D	NM_002878	c.494G>A	p.R165Q	0.333
Ctrl23	TP53	NM_000546	c.215C>G	p.P72R	0.418

Ctrl24	PTCH1	NM_000264.4	c.2680G>A	p.D894N	0.490
Ctrl24	RRAS	NM_006270.4	c.529C>T	p.L177F	0.506
Ctrl24	FANCE	NM_021922.2	c.1349C>T	p.T450I	0.445
Ctrl24	FANCD2	NM_033084.4	c.1195A>G	p.I399V	0.231
Ctrl24	PTPRS	NM_130854.2	c.1852C>T	p.R618C	0.536
Ctrl24	APC	NM_000038	c.5465T>A	p.V1822D	1.000
Ctrl24	ATM	NM_000051	c.5948A>G	p.N1983S	1.000
Ctrl24	ATR	NM_001184	c.632T>C	p.M211T	0.495
Ctrl24	AXIN2	NM_004655	c.148C>T	p.P50S	0.498
Ctrl24	BARD1	NM_000465	c.1519G>A	p.V507M	0.469
Ctrl24	BARD1	NM_000465	c.1134G>C	p.R378S	0.503
Ctrl24	BARD1	NM_000465	c.70C>T	p.P24S	0.519
Ctrl24	BMPR1A	NM_004329	c.4C>A	p.P2T	1.000
Ctrl24	BRCA1	NM_007294	c.3548A>G	p.K1183R	0.507
Ctrl24	BRCA1	NM_007294	c.4837A>G	p.S1613G	0.475
Ctrl24	BRCA1	NM_007294	c.2726A>T	p.N909I	0.467
Ctrl24	BRCA1	NM_007294	c.3113A>G	p.E1038G	0.500
Ctrl24	BRCA1	NM_007294	c.2612C>T	p.P871L	0.507
Ctrl24	BRCA2	NM_000059	c.865A>C	p.N289H	0.427
Ctrl24	BRCA2	NM_000059	c.2971A>G	p.N991D	0.451
Ctrl24	BRCA2	NM_000059	c.7397T>C	p.V2466A	1.000
Ctrl24	BRIP1	NM_032043	c.2755T>C	p.S919P	0.500
Ctrl24	CHEK1	NM_001114121	c.1411A>G	p.I471V	1.000
Ctrl24	EPCAM	NM_002354	c.344T>C	p.M115T	0.493
Ctrl24	HNF1A	NM_000545	c.1720A>G	p.S574G	1.000
Ctrl24	MUTYH	NM_012222	c.1005G>C	p.Q335H	0.488
Ctrl24	NBN	NM_002485	c.553G>C	p.E185Q	1.000
Ctrl24	PMS2	NM_000535	c.1621A>G	p.K541E	1.000
Ctrl24	PMS2	NM_000535	c.1454C>A	p.T485K	1.000
Ctrl24	POLE	NM_006231	c.755C>T	p.A252V	0.457
Ctrl24	STK11	NM_000455	c.1062C>G	p.F354L	0.462
Ctrl24	TSC1	NM_000368	c.965T>C	p.M322T	0.519
