# Supplementary figures and images for: Cell-Free DNA Sequencing of Intraocular Fluid as Liquid Biopsy in the Diagnosis of Vitreoretinal Lymphoma
Source: Front Oncol. 2022 Jul 19;12:932674. doi: 10.3389/fonc.2022.932674 (PMC9343589; doi:10.3389/fonc.2022.932674)

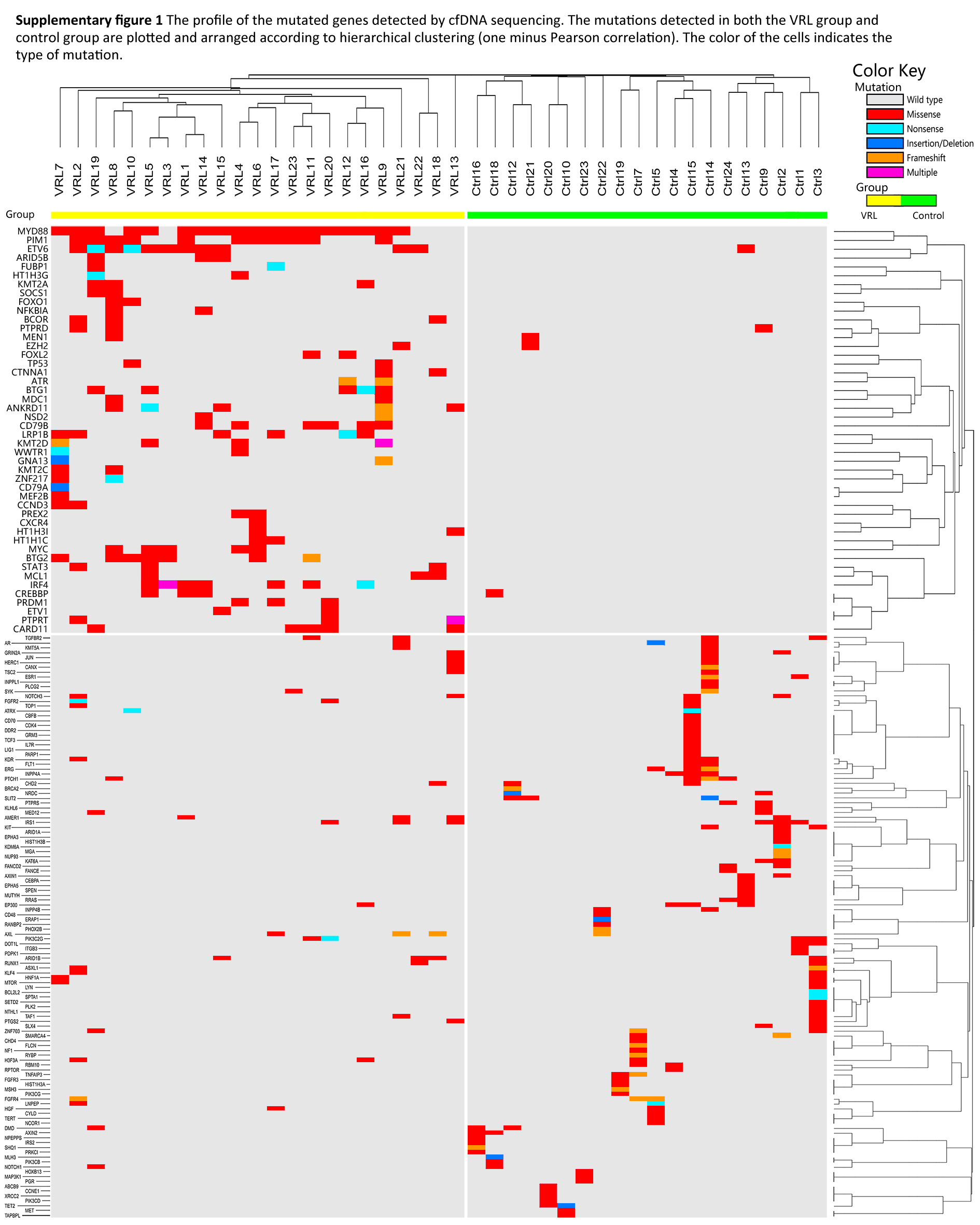

Supplement: Supplementary file 3 [file Image_1.tif]
